# A phase I dose-escalation study of Selumetinib in combination with Erlotinib or Temsirolimus in patients with advanced solid tumors

**DOI:** 10.1007/s10637-017-0459-7

**Published:** 2017-04-19

**Authors:** Jeffrey R. Infante, Roger B. Cohen, Kevin B. Kim, Howard A. Burris, Gregory Curt, Ugochi Emeribe, Delyth Clemett, Helen K. Tomkinson, Patricia M. LoRusso

**Affiliations:** 1 Sarah Cannon Research Institute, 93 Harley St, Marylebone, London, W1G 6AD UK; 2 Tennessee Oncology, PLLC, 250 25th Ave North, Nashville, TN 37203 USA; 3 Fox Chase Cancer Center, 333 Cottman Avenue, Philadelphia, PA 19111 USA; 4 California Pacific Medical Center (Sutterhealth), 475 Brannan Street, Suite 220, San Francisco, CA 94107 USA; 5 AstraZeneca, 1800 Concord Pike, Wilmington, DE 19850 USA; 6 AstraZeneca, Charter Way, Macclesfield, SK10 2NA UK; 7 Yale Cancer Center, 55 Park Street, Ste First Floor, New Haven, CT 06519 USA

**Keywords:** Selumetinib, Dose-escalation, Advanced solid tumors, Erlotinib, Temsirolimus

## Abstract

*Background* Combinations of molecularly targeted agents may provide optimal anti-tumor activity and improve clinical outcomes for patients with advanced cancers. Selumetinib (AZD6244, ARRY-142886) is an oral, potent and highly selective, allosteric inhibitor of MEK1/2, a component of the RAS/RAF/MEK/ERK pathway which is constitutively activated in many cancers. We investigated the safety, tolerability, and pharmacokinetics (PK) of selumetinib in combination with molecularly targeted drugs erlotinib or temsirolimus in patients with advanced solid tumors. *Methods* Two-part study: dose escalation, to determine the maximum tolerated dose (MTD) of selumetinib in combination with erlotinib 100 mg once daily (QD) or temsirolimus 25 mg once weekly, followed by dose expansion at the respective combination MTDs to further investigate safety and anti-tumor effects. *Results* 48 patients received selumetinib plus erlotinib and 32 patients received selumetinib plus temsirolimus. The MTD with erlotinib 100 mg QD was selumetinib 100 mg QD, with diarrhea being dose limiting. The most common all grade adverse events (AEs): diarrhea, rash, nausea, and fatigue. Four (8.3%) patients had ≥12 weeks stable disease. The MTD with temsirolimus 25 mg once weekly was selumetinib 50 mg twice daily (BID), with mucositis and neutropenia being dose limiting. The most commonly reported AEs: nausea, fatigue, diarrhea, and mucositis. Ten (31.3%) patients had ≥12 weeks stable disease. The combination PK profiles were comparable to previously observed monotherapy profiles. *Conclusions* MTDs were established for selumetinib in combination with erlotinib or temsirolimus. Overlapping toxicities prevented the escalation of selumetinib to its recommended phase II monotherapy dose of 75 mg BID. **Trial registration:** ClinicalTrials.gov NCT00600496; registered 8 July 2009.

**Electronic supplementary material:**

The online version of this article (doi:10.1007/s10637-017-0459-7) contains supplementary material, which is available to authorized users.

## Introduction

The intracellular RAS/RAF/MEK/ERK (RAS-ERK) pathway converges on MEK1/2, whose only known substrates are ERK1/2. Constitutive activation of the pathway is implicated in cell proliferation and is central to driving cancer growth and progression [1, 2]. Inhibition of MEK1/2 activity prevents transduction of the mitogenic signals from multiple pathways, resulting in effects on tumor proliferation, differentiation, and survival. These observations have been translated to the clinic and have improved outcomes for patients with advanced cancer. Indeed, the MEK inhibitor trametinib is recommended as monotherapy and in combination for patients with *BRAF* V600 mutant melanoma [3]. Several other MEK inhibitors are currently undergoing clinical investigation [4].

Selumetinib (AZD6244, ARRY-142886) is an oral, potent and highly selective, allosteric MEK1/2 inhibitor [5] with a short half-life [6, 7] currently in development for a variety of tumor types [8, 9]. In vitro cell viability experiments have demonstrated the inhibitory activity of selumetinib in a variety of human tumor cell lines [1]. In the first-in-human trial of selumetinib monotherapy [5], the maximum tolerated dose (MTD) was 75 mg twice daily (BID) and the most common adverse events (AEs) at this dose were fatigue, acneiform dermatitis, nausea, diarrhea, and peripheral edema. Since then, clinical activity of selumetinib monotherapy has been demonstrated in some patients with advanced melanoma, pancreatic cancer, non-small-cell lung cancer, and colorectal cancer [10–13].

The ability to simultaneously inhibit both the RAS-ERK pathway and other oncogenic signaling pathways, such as the PI3K/AKT/mTOR pathway or epidermal growth factor receptor (EGFR) signaling, holds significant promise; dual pathway inhibition can enhance inhibition of tumor cell growth and delay development of resistance to therapy [14, 15]. In tumor models of metastatic pancreatic and hepatocellular carcinoma, the combination of selumetinib with the mTOR inhibitor rapamycin enhanced anti-tumor activity compared with either agent alone [16, 17]. Additionally, the combination of selumetinib and gefitinib, an EGFR-tyrosine kinase inhibitor (TKI), showed synergistic effects on growth inhibition of nasopharyngeal cancer cell lines [15].

In light of these preclinical observations, the objectives of this phase I, dose-escalation study were to assess the safety, tolerability, pharmacokinetics (PK), and MTD of selumetinib in combination with four different anticancer therapies (docetaxel, dacarbazine, erlotinib, or temsirolimus) in patients with advanced solid tumors. Results for patients with advanced solid tumors who received selumetinib in combination with the targeted drugs erlotinib or temsirolimus are presented herein. An exploratory assessment of tumor response was also conducted.

## Materials and methods

This open-label, multicenter, phase I, two-part, dose-escalation study (ClinicalTrials.gov NCT00600496) was conducted in four centers in the USA between 14 December 2007 and 20 August 2010 (data cut-off occurring 6 months after the last patient began treatment). All patients provided written informed consent and the study was conducted in accordance with Good Clinical Practice guidelines and the Declaration of Helsinki. The protocol was approved by the institutional review board at each study site (**Supplementary material 1**: Supplementary Table 1; **Supplementary material 2**: study protocol).

### Patient selection

Patients eligible for the study were those with advanced solid tumors for whom erlotinib or temsirolimus would be an appropriate standard of care, or those who might benefit from erlotinib or temsirolimus combined with a novel agent such as selumetinib. Other eligibility criteria included: aged ≥18 years; measurable and/or non-measurable disease lacking curative options; World Health Organization (WHO) performance status 0–1; evidence of post-menopausal status or negative urine/serum pregnancy test for pre-menopausal female patients; and calculated creatinine clearance >50 mL/min.

Patients with any of the following were excluded from the study: prior treatment with a MEK inhibitor; received an investigational drug within 30 days of entering the study, or had not recovered from the AEs of an investigational study drug; received radiotherapy or standard chemotherapy within 21 days of study entry; use of strong cytochrome 1A2 or 3A4 inducers and/or inhibitors; brain metastases or spinal cord compression unless treated and stable (>1 month) and off steroids; factors that increased the risk of QT prolongation or arrhythmic events or QTc interval of >450 ms for males or >470 ms for females; inadequate bone marrow, hepatic, cardiac, or renal function; current smoker or user of any tobacco (erlotinib arm only).

### Study design and dosing

The study was conducted in two parts: dose escalation part A enrolled cohorts of three to six evaluable patients and assessed the safety, tolerability, PK, and MTD for selumetinib in combination with either erlotinib or temsirolimus; dose expansion part B further evaluated the safety, tolerability, and PK in a minimum of 12 additional patients at the MTD combination treatment of each regimen determined in part A. A safety review committee (SRC), comprising representatives from the study sponsor and at least one investigator, assessed the available safety and PK data. Dose-limiting toxicities (DLTs) in the study were defined as those associated with treatment occurring within the first 28 days of therapy from cycle 1/day 1. Hematologic DLTs were defined as afebrile grade 4 neutropenia for >5 days, grade 4 neutropenia associated with fever, or grade 4 thrombocytopenia. Non-hematological DLTs were defined as ≥grade 3 AEs for >7 days that could not be controlled to grade ≤ 2 with appropriate treatment.

Patients received erlotinib 100 mg orally once daily (QD) or temsirolimus 25 mg intravenously over 60 mins on days 1, 8, and 15 of each 21-day cycle. The 100 mg dose for erlotinib was chosen as this is the dose recommended for use in combination with gemcitabine for locally advanced or metastatic pancreatic cancer [18, 19]. The dose of temsirolimus could be reduced to 15 mg if the DLT(s) observed for the cohort was a known side effect of temsirolimus. The PK of selumetinib alone was assessed after a single monotherapy dose administered 3 to 8 days prior to combination therapy beginning on cycle 1/day 1 for the erlotinib and temsirolimus treatment arms. Selumetinib at a starting dose of 50 mg BID began on cycle 1/day 8.

Patients were enrolled into part A in initial cohorts of three to six patients. Subsequent dose levels were determined by the SRC, which reviewed the emerging tolerability and safety data on an ongoing basis and upon completion of each dose level cohort. In addition, the predicted exposure to selumetinib at each dose level evaluated was not to exceed the exposures previously observed at the monotherapy MTD of 75 mg BID [5]. Patients were considered evaluable if they had received at least 28 days of therapy from cycle 1/day 1, received approximately 80% of the planned doses of selumetinib, had experienced a DLT, or at the discretion of the SRC. The combination MTD in this study was defined as the highest selumetinib dose achieved at which no more than one of six evaluable patients experienced a DLT. In part B (dose expansion) of the study, an additional 12 evaluable patients for each combination received treatment at the previously defined combination MTD. Administration of selumetinib monotherapy was also allowed after erlotinib or temsirolimus had been discontinued if patients were considered to be deriving benefit.

Although selumetinib 50 mg BID plus erlotinib 100 mg QD was deemed to be tolerable in part A, the SRC determined that this combination dose was not tolerated during the part B expansion. The protocol was therefore amended to allow exploration of alternate dosing schedules of selumetinib 50 mg QD, 100 mg QD, and 150 mg QD plus erlotinib 100 mg QD.

### Assessments

#### Tolerability

Incidence and intensity of all AEs were graded using National Cancer Institute Common Terminology Criteria for Adverse Events (CTCAE) version 3.0. Other measures included vital signs (including blood pressure, pulse rate, weight, and body temperature); electrocardiogram; Multi-Gated Acquisition scan; echocardiogram; clinical chemistry; brain natriuretic peptide; troponin I; hematology; urinalysis; and ophthalmologic examinations. Incidence of DLTs was also recorded.

#### Pharmacokinetics assessments

Blood samples for PK assessments were collected pre-dose and at 0.5, 1, 1.5, 2, 4, 8, 12, 24, and 72–192 h post-dose. The following PK parameters of selumetinib, N-desmethyl selumetinib, erlotinib, and temsirolimus were determined following administration alone and in combination: maximum plasma concentration (C_max_), time to reach the C_max_ (t_max_), area under the plasma concentration-time curve from 0 to 12 h post dose (AUC_(0–12)_), and from 0 to 8 h post dose (AUC_(0–8)_).

For the erlotinib arm, selumetinib PK blood sampling was performed after a single dose of selumetinib 3 to 8 days prior to cycle 1 (alone) and on cycle 1/day 8 (combination), and erlotinib PK sampling was performed on cycle 1/day 7 (alone) and cycle 1/day 8 (combination). For the temsirolimus arm, selumetinib PK sampling was performed after a single dose of selumetinib 3 to 8 days prior to cycle 1 (alone) and on cycle 1/day 8 (combination), and temsirolimus PK sampling was performed on cycle 1/day 1 (alone) and on cycle 1/day 8 (combination).

PK parameters were derived using non-compartmental analysis. C_max_ and t_max_ were determined by visual inspection of the plasma concentration-time profiles. AUC_(0–12)_ was calculated by the linear trapezoidal rule. Where more than one maximum occurred, the reported value was assigned to the first occurrence.

#### Tumor response

Tumor response was assessed according to Response Evaluation Criteria In Solid Tumors (RECIST) (version 1.0). Baseline tumor assessments were performed up to 4 weeks before the planned first dose of selumetinib. Subsequent tumor assessments were conducted prior to the third cycle, every alternate cycle thereafter, and on withdrawal of treatment.

#### EGFR and KRAS mutation analyses

Mutation status was an exploratory endpoint and an optional part of the protocol. DNA was extracted from formalin-fixed paraffin-embedded tissue samples using the Cobas™ DNA Sample Preparation kit (Roche Molecular Systems, Inc., Pleasanton, CA, USA). Plasma DNA was extracted using a non-commercial plasma preparation kit (Roche). DNA was assayed using the Cobas™ KRAS Mutation Test and Cobas™ EGFR Mutation Test (Roche) according to the manufacturer’s protocols. The *EGFR* mutation assay covered the following mutations: exon 18 G719X (G719A, G719C, and G719S); exon 19 deletions and complex mutations; exon 20 S768I, T790 M, and insertions; exon 21 L858R. The *KRAS* mutation assay covered mutations in codons 12, 13 (exon 2), and 61 (exon 3). Data were generated and analysed using the Cobas z480. One sample from a patient receiving selumetinib in combination with erlotinib was retrospectively analyzed using Sequenom iPlex covering known *KRAS* and *EGFR* mutations at Newgene Ltd. (Newcastle-upon-Tyne, UK), following the manufacturer’s standard protocol [20].

### Statistical analysis

Data cut-off was 20 August 2010. The safety population included all patients who received one or more doses of selumetinib and partner drug treatment. Patients were considered evaluable for dose escalation if they had received approximately 80% of the planned doses of selumetinib in cycle 1, completed at least 28 days of therapy from cycle 1/day 1, provided PK data, and had all safety evaluations performed or experienced a DLT, or at the discretion of the SRC. The ‘evaluable for PK analysis’ population included all patients with concentration-time data available.

No formal statistical hypothesis testing was performed on the data.

## Results

### Patient characteristics, disposition, and duration of treatment

Between 14 December 2007 and 20 August 2010, a total of 48 patients received selumetinib plus erlotinib and 32 received selumetinib plus temsirolimus. Baseline patient and disease characteristics in the study populations were similar in the two combination arms (Table 1). The majority of patients had two to three prior therapies and the most common tumor types were colorectal, lung, pancreatic, and renal cancer.

**Table 1:** Patient and disease baseline characteristics

Characteristic, n (%)	Selumetinib + erlotinib 100 mg (*N* = 48)	Selumetinib + temsirolimus 25 mg (*N* = 32)
Age, years; mean (SD)	59.1 (9.9)	57.2 (9.9)
Male	23 (47.9)	16 (50.0)
Female	25 (52.1)	16 (50.0)
Race		
White	44 (91.7)	30 (93.8)
Black/African American	3 (6.3)	2 (6.3)
Other	1 (2.1)	0
WHO performance status		
0	29 (60.4)	20 (62.5)
1	19 (39.6)	12 (37.5)
Primary tumor site^a^		
Colorectal	21 (43.8)^b^	11 (34.4)
Lung	12 (25.0)	3 (9.4)
Pancreas	5 (10.4)	3 (9.4)
Renal	2 (4.2)	4 (12.5)
Skin/soft tissue	2 (4.2)	3 (9.4)
Liver	2 (4.2)	1 (3.1)
Thyroid	0	2 (6.3)
Other	4 (8.3)^c^	5 (15.6)^d^
Mean prior systemic treatments	3.5	3.6
Prior therapy, n (%)		
Chemotherapy	44 (91.7)	29 (90.6)
Platinum compounds	36 (75.0)	19 (59.4)
Pyrimidine analogues	27 (56.3)	13 (40.6)
Taxanes	13 (27.1)	12 (37.5)
Anthracyclines	6 (12.5)	2 (6.3)
Radiotherapy	22 (45.8)	16 (50.0)
Other systemic anticancer therapy^e^	18 (37.5)	7 (21.9)
Immunotherapy	1 (2.1)	2 (6.3)
Immuno/hormonal therapy	1 (2.1)	1 (3.1)
Hormonal therapy	1 (2.1)	1 (3.1)
Prior lines of chemotherapy, n (%)		
0 or 1	7 (14.6)	7 (21.9)
2 or 3	27 (56.3)	21 (65.6)
4+	14 (29.2)	4 (12.5)

*SD* standard deviation, *WHO* World Health Organization

^a^For erlotinib, only sites in more than one patient are listed

^b^Includes colon, rectal, and colorectal

^c^Breast, bladder, and in two patients unknown site of primary tumor

^d^Includes bone (tibia), appendix, mucosal, esophagus, and uterus in one patient each

^e^Includes monoclonal antibodies, vaccines, small molecule targeted agents, and investigational drugs

**Fig. 1:**
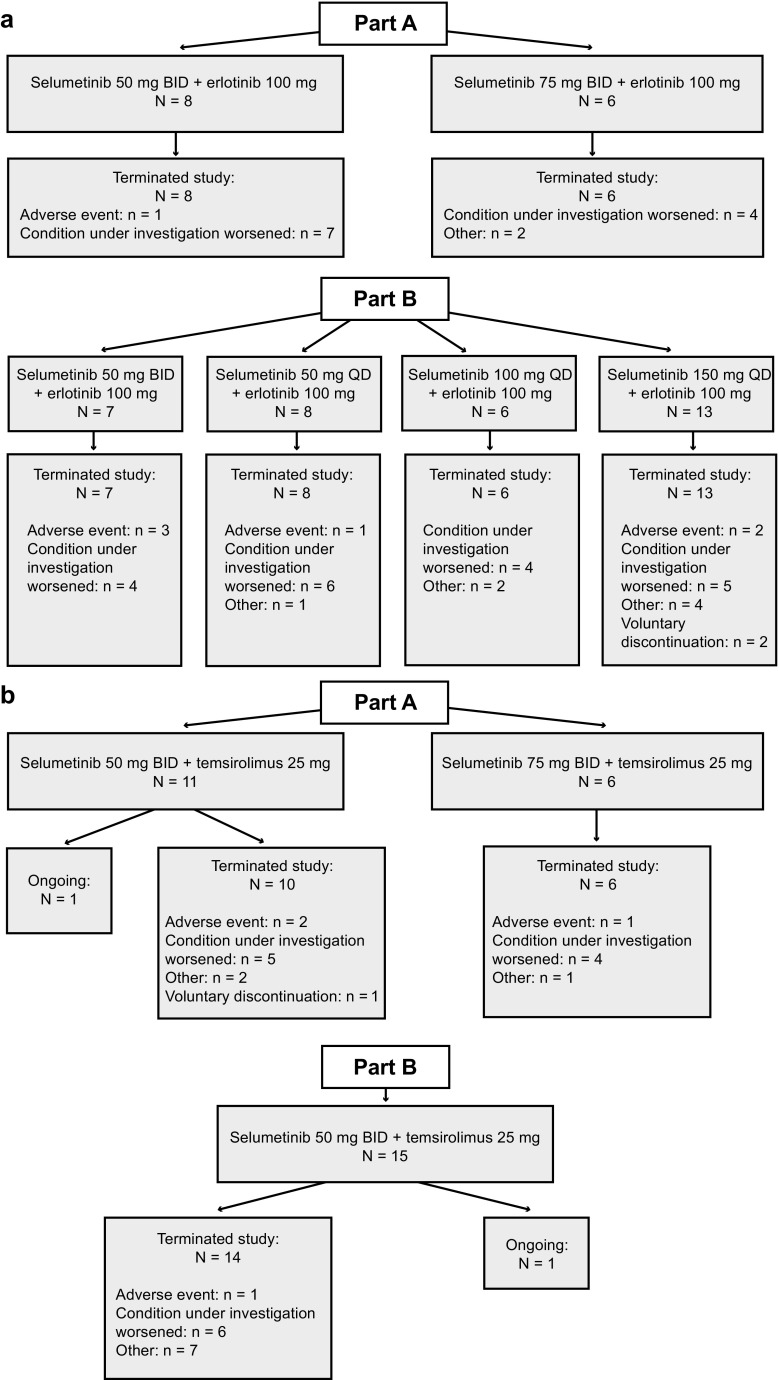
Patient disposition at the time of data cut-off in the **a** selumetinib plus erlotinib and **b** selumetinib plus temsirolimus arms. BID, twice daily; QD, once daily

The disposition of patients is summarized in Fig. 1. Among patients treated with selumetinib plus erlotinib, 14 were included in the dose-escalation phase (part A), and 34 were included in the dose-expansion phase (part B). The median duration of treatment with selumetinib (6–7 weeks) or erlotinib (5–6 weeks) was similar across dose cohorts. At the time of data cut-off (August 2010), all patients had discontinued treatment with selumetinib plus erlotinib.

Among patients treated with selumetinib plus temsirolimus, 17 were included in the dose-escalation phase (part A), and 15 were included in the dose-expansion phase (part B). Patients received selumetinib 50 mg BID for a median of ~8 weeks; 48% and 28% of patients received ≥3 cycles and ≥6 cycles, respectively, of temsirolimus in combination with selumetinib 50 mg BID. At the time of data cut-off, one patient each from part A and part B were still receiving study treatment (selumetinib 50 mg BID, *n* = 2), and have since discontinued. One patient with thyroid cancer received treatment for 1 year 9 months, followed by an additional 6 months’ treatment with selumetinib monotherapy. Another patient with renal cancer received the combination at reduced doses of selumetinib 25 mg QD, every other week, plus temsirolimus 15 mg/m^2^ every 21 days for 4 years 7 months, before discontinuing combination treatment due to disease progression.

### Selumetinib in combination with erlotinib

#### Dose-limiting toxicities

Of the 14 patients included in the dose-escalation phase (part A), eight patients received selumetinib 50 mg BID plus erlotinib and six patients received selumetinib 75 mg BID plus erlotinib. Although there were no AEs that met the formal protocol-defined DLT criteria for these doses, the overall tolerability profile of selumetinib 75 mg BID plus erlotinib led the SRC to deem this a non-tolerable dose (Table 2). DLTs of grade 3 diarrhea occurred in two patients in this cohort. Selumetinib 50 mg BID plus erlotinib was therefore determined to be the protocol-defined MTD and was used in the expansion phase of the study (part B).

**Table 2:** Summary of cohorts and dose escalation based on dose-limiting toxicity

Part	Selumetinib dose	n (evaluable for dose escalation)	Evaluable patients with a DLT	DLT information
Selumetinib in combination with erlotinib				
A	50 mg BID	8 (6)	0	-
	75 mg BID	6 (4)	2	Grade 3 diarrhea (*n* = 2)
B	50 mg BID	7 (5)	3^a^	Grade 3 rash; grade 3 diarrhea (*n* = 2)
	50 mg QD	8 (5)	0	-
	100 mg QD	6 (6)	0	-
	150 mg QD	13 (5)	0	-
Selumetinib in combination with temsirolimus				
A	50 mg BID	11 (6)	0	NA
	75 mg BID	6 (6)	2^a^	Grade 3 neutropenia and grade 1 mucosal inflammation; grade 2 mucosal inflammation
B	50 mg BID	15 (NA)	NA	NA

In the erlotinib combination arm, DLT criteria were applied to adverse events occurring in the part B dose expansion cohort, and to the subsequent QD dose exploration cohorts

*BID* twice daily, *DLT* dose-limiting toxicity, *NA* not applicable, *QD* once daily, *SRC* safety review committee

^a^Events did not meet the protocol-defined DLT criteria in these patients. The SRC agreed that these toxicities were sufficient to declare a non-tolerable dose

In part B, we observed a relatively high incidence of grade ≥ 3 diarrhea in patients receiving selumetinib 50 mg BID plus erlotinib (*n* = 3, 43%), similar to that reported in the equivalent cohort in part A (*n* = 2, 25%), and with the non-tolerated dose of selumetinib 75 mg BID plus erlotinib (*n* = 2, 33%) (Table 3). Three SRC-defined dose-limiting grade 3 AEs (diarrhea, *n* = 2 on days 14 and 20, respectively; and rash, *n* = 1 on day 12) occurred in three of the first seven patients enrolled into part B receiving selumetinib 50 mg BID plus erlotinib indicating intolerability of this combination dose and schedule.

**Table 3:** Adverse events: selumetinib in combination with erlotinib or temsirolimus

	Part A		Part B				Part A + B
	Selumetinib 50 mg BID (*N* = 8)	Selumetinib 75 mg BID (*N* = 6)	Selumetinib 50 mg BID (*N* = 7)	Selumetinib 50 mg QD (*N* = 8)	Selumetinib 100 mg QD (*N* = 6)	Selumetinib 150 mg QD (*N* = 13)	Selumetinib 50 mg BID (*N* = 15)
Selumetinib in combination with erlotinib AE category, n (%)							
Any AE	8 (100.0)	6 (100.0)	7 (100.0)	8 (100.0)	6 (100.0)	13 (100.0)	15 (100.0)
Any CTCAE grade ≥ 3	7 (87.5)	4 (66.7)	5 (71.4)	3 (37.5)	3 (50.0)	12 (92.3)	12 (80.0)
Any SAE	2 (25.0)	2 (33.3)	4 (57.1)	3 (37.5)	2 (33.3)	9 (69.2)	6 (40.0)
Any AE leading to discontinuation	1 (12.5)	1 (16.7)	1 (14.3)	2 (25.0)	0	1 (7.7)	2 (13.3)
Most frequently reported AEs (≥20% of all patients receiving selumetinib 50 mg BID + erlotinib), n (%)							
Diarrhea	5 (62.5)	5 (83.3)	6 (85.7)	5 (62.5)	6 (100.0)	11 (84.6)	11 (73.3)
Grade ≥ 3	2 (25.0)	2 (33.3)	3 (42.9)	1 (12.5)	1 (16.7)	1 (7.7)	5 (33.3)
Decreased appetite	4 (50.0)	1 (16.7)	4 (57.1)	3 (37.5)	1 (16.7)	3 (23.1)	8 (53.3)
Fatigue	2 (25.0)	4 (66.7)	3 (42.9)	1 (12.5)	2 (33.3)	4 (30.8)	5 (33.3)
Skin and subcutaneous tissue disorders	5 (62.5)	2 (33.3)	3 (42.9)	6 (75.0)	5 (83.3)	11 (84.6)	8 (53.3)
Dermatitis acneiform	3 (37.5)	2 (33.3)	2 (28.6)	5 (62.5)	5 (83.3)	8 (61.5)	5 (33.3)
Grade ≥ 3	1 (12.5)	1 (16.7)	2 (28.6)	0	1 (16.7)	2 (15.4)	3 (20.0)
Rash	2 (25.0)	0	0	1 (12.5)	0	3 (23.1)	2 (13.3)
Rash erythematous	1 (12.5)	0	0	0	0	0	1 (6.7)
Rash macular	0	1 (16.7)	0	0	0	0	0
Rash papular	0	0	0	0	0	2 (15.4)	0
Nausea	3 (37.5)	4 (66.7)	1 (14.3)	0	3 (50.0)	8 (61.5)	4 (26.7)
Edema peripheral	3 (37.5)	2 (33.3)	1 (14.3)	1 (12.5)	1 (16.7)	3 (23.1)	4 (26.7)
Pyrexia	3 (37.5)	1 (16.7)	1 (14.3)	1 (12.5)	0	3 (23.1)	4 (26.7)
Anemia	2 (25.0)	1 (16.7)	1 (14.3)	1 (12.5)	0	2 (15.4)	3 (20.0)
Dizziness	1 (12.5)	0	2 (28.6)	0	0	2 (15.4)	3 (20.0)
	Selumetinib 50 mg BID (*N* = 8)	Selumetinib 75 mg BID (*N* = 6)	Selumetinib 50 mg BID (*N* = 7)	-	-	-	Selumetinib 50 mg BID (*N* = 15)
Selumetinib in combination with temsirolimus AE category, n (%)							
Any AE	10 (90.9)	6 (100.0)	15 (100.0)	-	-	-	25 (96.2)
Any CTCAE grade ≥ 3	7 (63.6)	5 (83.3)	12 (80.0)	-	-	-	19 (73.1)
Any SAE	2 (18.2)	2 (33.3)	8 (53.3)	-	-	-	10 (38.5)
Any AE leading to discontinuation	2 (18.2)	1 (6.7)	1 (6.7)	-	-	-	3 (11.5)
Most frequently reported AEs (≥20% of all patients receiving selumetinib 50 mg BID + temsirolimus), n (%)							
Nausea	6 (54.5)	4 (66.7)	9 (60.0)	-	-	-	15 (57.7)
Fatigue	6 (54.5)	4 (66.7)	6 (40.0)	-	-	-	12 (46.2)
Mucosal inflammation	5 (45.5)	3 (50.0)	7 (46.7)	-	-	-	12 (46.2)
Decreased appetite	6 (54.5)	1 (16.7)	5 (33.3)	-	-	-	11 (42.3)
Diarrhea	5 (45.5)	5 (83.3)	6 (40.0)	-	-	-	11 (42.3)
Vomiting	5 (45.5)	2 (33.3)	6 (40.0)	-	-	-	11 (42.3)
Skin and subcutaneous disorders	6 (54.5)	1 (16.7)	12 (80.0)	-	-	-	18 (69.2)
Dermatitis acneiform	4 (36.4)	1 (16.7)	6 (40.0)	-	-	-	10 (38.5)
Rash	1 (9.1)	0	4 (26.7)	-	-	-	5 (19.2)
Rash erythematous	1 (9.1)	0	2 (13.3)	-	-	-	3 (11.5)
Rash macular	0	0	1 (6.7)	-	-	-	1 (3.8)
Rash maculo-papular	1 (9.1)	0	1 (6.7)	-	-	-	2 (7.7)
Rash papular	1 (9.1)	0	0	-	-	-	1 (3.8)
Edema peripheral	3 (27.3)	2 (33.3)	5 (33.3)	-	-	-	8 (30.8)
Thrombocytopenia	2 (18.2)	0	6 (40.0)	-	-	-	8 (30.8)
Constipation	3 (27.3)	1 (16.7)	4 (26.7)	-	-	-	7 (26.9)

Erlotinib, 100 mg orally QD; temsirolimus 25 mg intravenously over 60 mins on day 1, 8, and 15 of each 21-day cycle

*AE* adverse event, *BID* twice daily, *CTCAE* National Cancer Institute Common Terminology Criteria for Adverse Events, *QD* once daily; *SAE* serious adverse event

In order to define a more tolerable combination regimen, the SRC recommended exploration of selumetinib administered QD and the protocol was amended accordingly. No DLTs occurred in patients receiving selumetinib 50 mg QD (*n* = 8) or 100 mg QD (*n* = 6). The incidence of grade ≥ 3 diarrhea was lower in patients receiving selumetinib QD (Table 3), and we observed only one grade ≥ 3 diarrhea (17%) among those patients receiving the recommended phase II dose of selumetinib 100 mg QD plus erlotinib. AEs of diarrhea and rash occurring with selumetinib 150 mg QD were deemed non-tolerable. Thus, selumetinib 100 mg QD plus erlotinib 100 mg QD was the dose recommended for future phase II studies.

At least one patient in all but one dose group (part B selumetinib 50 mg BID plus erlotinib) had a selumetinib dose reduction or interruption. No patient had more than one dose reduction or three dose interruptions, except for one patient receiving selumetinib 50 mg BID plus erlotinib in part B who had 10 dose interruptions.

#### Tolerability

The most commonly reported AEs in patients receiving any dose of selumetinib plus erlotinib were: diarrhea (38/48 patients, 79%), dermatitis acneiform (25/48, 52%), nausea (19/48, 40%), decreased appetite (16/48, 33%), and fatigue (16/48, 33%) (Table 3). The most commonly reported grade ≥ 3 AEs were: diarrhea (10/48 patients, 21%), dermatitis acneiform (7/48, 15%), hypokalemia (5/48, 10%), and dehydration, fatigue, and hyponatremia (3/48, 6% each). Grade 4 events were reported in six patients (neutropenia and myocardial infarction, hypokalemia, fatigue, cerebrovascular accident [*n* = 2], exertional dyspnea). All but one of the grade 4 events (fatigue in a patient receiving selumetinib 75 mg BID plus erlotinib) were considered to be unrelated to study treatment. There were no AEs leading to death.

#### Pharmacokinetics analysis

PK parameters of selumetinib, N-desmethyl selumetinib, and erlotinib were similar when administered alone and in combination (selumetinib 50 mg BID and 75 mg BID summarized in Table 4 and selumetinib 50 mg QD, 100 mg QD, and 150 mg QD summarized in **Supplementary material 1**: Supplementary Table 2). Plasma concentration time profiles of selumetinib, N-desmethyl selumetinib, and erlotinib were also similar when administered alone or in combination (**Supplementary material 1**: Supplementary Fig. 1).

**Table 4:** Pharmacokinetic parameters of selumetinib following dosing alone and in combination with erlotinib or temsirolimus

Geometric mean (% co-efficient of variation) [n evaluable]								
	Selumetinib + erlotinib				Selumetinib + temsirolimus			
	Dosed alone		Dosed in combination		Dosed alone		Dosed in combination	
Parameter	Selumetinib 50 mg (*N* = 14)	Selumetinib 75 mg (*N* = 6)	Selumetinib 50 mg BID (*N* = 12)	Selumetinib 75 mg BID (*N* = 6)	Selumetinib 50 mg (*N* = 25)	Selumetinib 75 mg (*N* = 6)	Selumetinib 50 mg BID (*N* = 22)	Selumetinib 75 mg BID (*N* = 5)
Selumetinib								
C_max_, ng/mL	926.5 (69.10)	1051 (43.60)	1334 (51.16)	1657 (55.70)	666.1 (108.9)	1237 (60.02)	675.5 (63.07)	887.4 (65.74)
t_max_, h^a^	1.25 (1.0–8.0)	1.25 (1.0–2.0)	1.0 (0.5–2.0)	1.0 (1.0–1.5)	1.5 (0.5–4.0)	1.5 (1.0–4.0)	1.5 (0.5–2.0)	1.5 (1.0–4.0)
AUC_(0–8)_, ng•h/mL	2279 (41.20) [13]	2863 (39.10)	3638 (29.12) [11]	4348 (36.70)	1839 (68.29)	2989 (41.53)	1840 (49.77)	2518 (44.21)
AUC_(0–12)_, ng•h/mL	2699 (32.67) [13]	3197 (39.80)	4011 (29.56) [11]	4826 (36.70)	2147 (59.92)	3368 (38.32)	2128 (48.74) [15]	2938 (51.63) [4]
N-desmethyl selumetinib								
C_max_, ng/mL	41.69 (60.64)	66.80 (75.74)	43.37 (47.62)	72.94 (91.01)	35.28 (109.00)	75.34 (88.64)	38.51 (81.60)	48.36 (65.63)
t_max_, h^a^	1.5 (1.0–12.0)	1.45 (1.0–2.0)	1.25 (1.0–4.0)	1.5 (1.0–2.0)	1.5 (1.0–8.0)	1.5 (1.0–4.0)	2.0 (1.0–2.0)	2.0 (1.0–2.0)
AUC_(0–8)_, ng•h/mL	141.3 (41.79) [13]	195.9 (36.04)	130.4 (39.09)	227.5 (57.77)	124.3 (80.50)	231.1 (68.07)	129.4 (59.96)	167.8 (51.40)
AUC_(0–12)_, ng•h/mL	173.4 (39.75) [12]	223.9 (34.90)	153.3 (39.37)	262.7 (53.29)	168.2 (54.21) [23]	269.4 (62.60)	168.2 (57.86) [14]	190.5 (55.06) [4]
Erlotinib								
C_max_, ng/mL	1681 (27.13) [13]	1416 (64.33)	1725 (30.11)	1739 (39.86)				
t_max_, h^a^	2.0 (0.5–8.0)	3.0 (2.0–6.0)	1.5 (0.5–3.0)	2.0 (1.0–6.0)				
AUC_(0–8)_, ng•h/mL	10,080 (30.53) [13]	9594 (67.01)	10,360 (32.46)	10,370 (45.25)				
AUC_(0–12)_, ng•h/mL	14,500 (29.90) [13]	13,760 (69.54)	14,690 (33.08)	14,740 (45.19)				
Temsirolimus								
C_max_, ng/mL					668.8 (16.11) [24]	749.2 (24.61) [5]	650.7 (21.32)	694.6 (23.06)
t_max_, h^a^					0.5 (0.5–1.0)	0.5 (0.5–1.0)	1.0 (0.5–1.5)	0.5 (0.5–1.0)
AUC_(0–8)_, ng•h/mL					1578 (16.56) [24]	1736 (22.61) [5]	1460 (17.23)	1538 (18.38)
AUC_(0–12)_, ng•h/mL					1875 (18.26) [24]	1910 (19.90) [4]	1608 (10.96) [18]	1743 (20.14) [4]
Sirolimus								
C_max_, ng/mL					58.49 (56.97) [24]	49.48 (52.51) [5]	67.01 (48.91)	51.21 (42.52)
t_max_, h^a^					1.5 (1.5–24.0)	2.0 (1.5–2.0)	1.5 (1.5–4.0)	2.0 (1.5–3.0)
AUC_(0–8)_, ng•h/mL					341.1 (57.04) [24]	266.4 (36.14) [5]	393.8 (49.36)	292.4 (42.86)
AUC_(0–12)_, ng•h/mL					513.5 (54.99) [24]	381.8 (31.79) [5]	584.2 (52.72) [18]	360.2 (24.29) [4]

Erlotinib, 100 mg orally QD; temsirolimus 25 mg intravenously over 60 mins on day 1, 8, and 15 of each 21-day cycle

AUC_(0–8)_, area under the concentration-time curve from 0 to 8 h; AUC_(0–12)_, area under the concentration-time curve from 0 to 12 h; BID, twice daily; C_max_, maximum plasma concentration; QD, once daily; t_max_, time to reach the maximum plasma concentration

^a^Median value and range

#### Tumor response

**Fig. 2:**
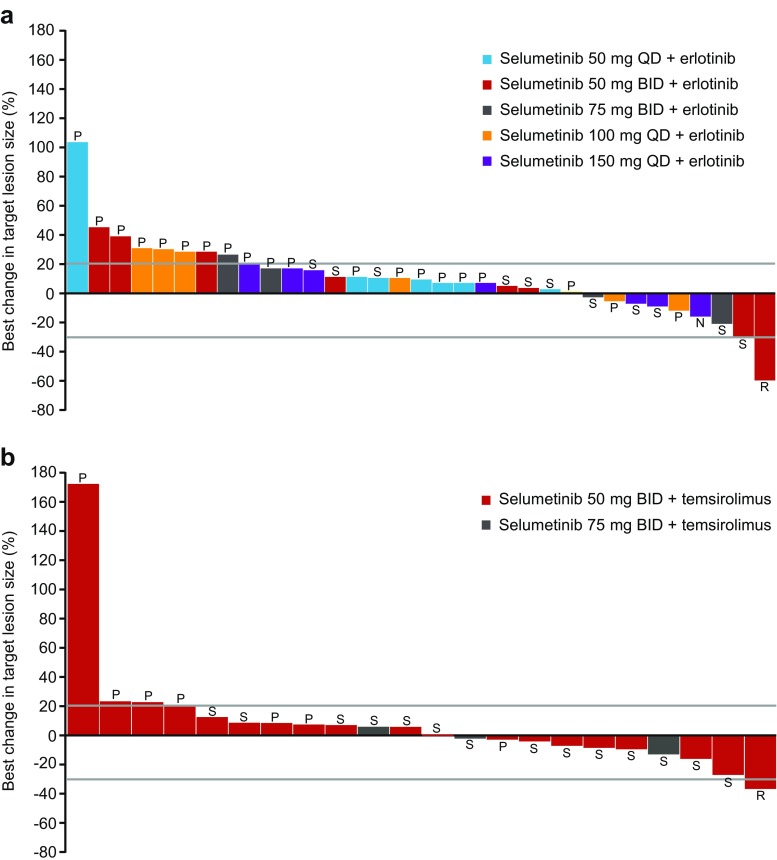
Waterfall plots for best change in target lesion size from baseline for the **a** selumetinib plus erlotinib and **b** selumetinib plus temsirolimus arms. Lower reference line indicates the point below which best response is partial response (>30% reduction). Upper reference line indicates the point above which best response is progressive disease (>20%). Response Evaluation Criteria In Solid Tumors best response: N, not evaluable; P, progressive disease; R, partial response; S, stable disease. BID, twice daily; QD, once daily. Population: Measurable disease at baseline and underwent follow-up scan (Figures created in Adobe Illustrator CC 2015)

Thirty-six patients were evaluable for response. One patient with an unknown primary tumor with lung, liver, and bone metastases receiving selumetinib 50 mg BID plus erlotinib had a confirmed partial response with a duration of 218 days; mutation analysis of a biopsied hepatic metastasis identified an *EGFR* exon 19 deletion. One patient with lung cancer receiving selumetinib 50 mg BID plus erlotinib had an unconfirmed partial response; *KRAS* and *EGFR* mutations were not detected. Eleven patients had a best response of stable disease ≥6 weeks and 24 had disease progression. At week 12, four (8.3%) patients had stable disease and one (2.1%) patient had an objective response. Two patients who received selumetinib 50 mg BID plus erlotinib had a total duration of treatment >6 months at data cut-off: 500 days for one patient with a primary lung tumor; and 267 days for one patient with primary tumor recorded as ‘other’. The best change from baseline in target lesion size is shown in Fig. 2a.

### Selumetinib in combination with temsirolimus

#### Dose-limiting toxicities

Selumetinib 75 mg BID plus temsirolimus was not tolerated. Two of three evaluable patients at this dose level had DLTs (grade 3 neutropenia and grade 1 mucositis; grade 2 mucositis). Although these events did not meet the protocol-defined DLT criteria, the SRC agreed that these toxicities were sufficient to declare a non-tolerable dose (Table 2). There were no DLTs among the 11 patients receiving selumetinib 50 mg BID plus temsirolimus and this dose was considered the MTD for the combination.

#### Tolerability

The most frequently reported AEs among patients (*n* = 32) receiving selumetinib plus temsirolimus were: nausea (19/32 patients, 59%), diarrhea (16/32, 50%), fatigue (16/32, 50%), and mucositis (15/32, 47%) (Table 3). The most commonly reported grade ≥3 AEs were mucositis (3/32 patients, 9%) and anemia, diarrhea, and neutropenia (2/32, 6% each). Grade 4 AEs of respiratory arrest, hyperglycemia, and increased blood uric acid were reported in three patients and were considered unrelated to selumetinib. The need for selumetinib dose reductions and dose interruptions was similar across cohorts, with AEs being the main reason for dose interruption (7/9, 78%). There were no AEs leading to death.

#### Pharmacokinetics analysis

PK parameters of selumetinib, N-desmethyl selumetinib, and temsirolimus (summarized in Table 4) were similar when administered alone and in combination. Plasma concentration time profiles of selumetinib, N-desmethyl selumetinib, and temsirolimus were also similar when administered alone or in combination (**Supplementary material 1**: Supplementary Fig. 2).

#### Tumor response

In total, 22 patients were evaluable for radiographic response. One patient with a skin/soft tissue tumor who was receiving selumetinib 50 mg BID plus temsirolimus had a confirmed partial response, lasting 117 days; *KRAS* or *EGFR* mutations were not detected. Fourteen patients had a best response of stable disease ≥6 weeks and seven had disease progression. At week 12, 10 (31.3%) patients had stable disease and 1 (3.1%) patient had an objective response. Eight patients had a total duration of treatment >6 months at data cut-off. Primary tumor locations (duration of treatment) of patients receiving selumetinib 50 mg BID plus temsirolimus were recorded as: colorectal (246 days), renal (2 patients: 470 and 238 days), colon (219 days), skin/soft tissue (249 days), and thyroid (615 days). For the two patients receiving selumetinib 75 mg BID plus temsirolimus: appendix (410 days) and colorectal (182 days). Best change from baseline in target lesion size is shown in Fig. 2b.

## Discussion

MEK inhibitors provide a highly specific method of inhibiting the cell survival and proliferation signals generated by the constitutively activated RAS-ERK pathway. Given the complexity of the signaling pathways involved in tumorigenesis, combinations of drugs targeting different pathways, or multiple elements in the same pathway, may provide better anti-tumor activity and ultimately improve clinical outcomes. This strategy has already proven successful with the addition of the MEK inhibitor trametinib to the BRAF inhibitor dabrafenib, improving both response rates and progression-free survival compared with dabrafenib alone in patients with BRAF V600 mutant melanoma [21].

The combination trial reported here was designed with the goal of escalating to the full recommended phase II dose of selumetinib 75 mg BID, in combination with other targeted agents [5, 22]. However, this was not achieved with the erlotinib or the temsirolimus combinations. Selumetinib 100 mg QD was the MTD in combination with erlotinib 100 mg QD (the recommended dose of erlotinib in combination with gemcitabine [18, 19]), while selumetinib 50 mg BID was the MTD in combination with temsirolimus 25 mg (days 1, 8, and 15 of each 21-day cycle; the recommended dose for single agent temsirolimus [23, 24]). These findings are similar to those reported from other studies of selumetinib in combination with targeted drugs such as the multikinase inhibitor sorafenib, the Akt inhibitor MK-2206, or the IGFR-R1 inhibitor IMC-A12, in which the tolerated combination dose of selumetinib was lower than the recommended phase II monotherapy dose [25–27].

Administration of selumetinib in combination with either erlotinib or temsirolimus did not alter the PK profiles of any of the drugs. Therefore, the accentuation of the known AEs of the targeted drugs could not be explained by an unexpected change in exposure. Furthermore, the PK profile of selumetinib alone was comparable to that observed in previous studies of selumetinib monotherapy [5, 28].

In our study the overall AE profiles were generally consistent with the known profiles of the individual drugs [12, 23, 29]. The tolerability of the combinations appears to have been limited by the accentuation of these toxicities when given in combination. With the selumetinib plus erlotinib combination, diarrhea, nausea, fatigue, and rash were common and diarrhea was the predominant DLT. Although no formal DLTs were observed with selumetinib 50 mg BID plus erlotinib in part A of the study, this dose was deemed non-tolerable after three patients in the expansion cohort (part B) experienced grade 3 AEs (diarrhea, *n* = 2; rash, *n* = 1). This experience also highlights a potential limitation of the conventional design for dose escalation trials evaluating chronically administered orally available drugs, in which formal DLTs are defined based on events in the first treatment cycle and do not take into account the occurrence of such toxicities on an ongoing basis with an oral therapy administered daily [30]. Knowing the relatively short half-life [5, 7], a QD dosing schedule of selumetinib was explored based on the hypothesis that a higher C_max_ and a lower overall AUC would achieve target inhibition in tumors, while allowing time for recovery in normal cells. Indeed, tolerability was improved with the selumetinib 100 mg QD dosing regimen.

The tolerability profile of selumetinib and erlotinib observed in our study has been borne out by subsequent phase II trials of the combination in which dose reductions were required in a considerable proportion of patients, with diarrhea and rash being the prominent grade ≥3 AEs [31–33]. Notably, in a phase II trial using the MTD of selumetinib 100 mg QD in combination with erlotinib 100 mg QD, significant toxicities were reported in 87% of patients, with grade 3 or 4 AEs occurring in >20% of patients [33]. These preliminary reports highlight the challenges of combining targeted drugs when they have overlapping AE profiles. Further reports have highlighted difficulties in combining other targeted agents with EGFR-TKIs [34]. We wait to see whether T790M directed EGFR-TKIs, such as osimertinib (AZD9291), which reportedly has less associated diarrhea and rash than erlotinib [35, 36], will be better tolerated in combination with MEK inhibitors. Preliminary results from the TATTON trial (NCT02143466) indicate that the combination of selumetinib and osimertinib at the respective recommended phase II doses is tolerated [37].

Similar to the erlotinib combination, diarrhea, nausea, and fatigue were also frequently reported with the temsirolimus combination. Mucositis was also common and dose limiting in patients receiving selumetinib plus temsirolimus and defined the MTD of selumetinib 50 mg BID for this combination. Interestingly, the combination of the targeted agents dabrafenib and trametinib is associated with more MEK-inhibitor related toxicities but fewer BRAF-inhibitor associated toxicities, and an improved health-related quality of life compared with single agent dabrafenib [38–41], demonstrating that combination of certain targeted agents is still a viable strategy.

Very limited clinical activity was observed in our study, with only one objective response noted with each selumetinib combination. The low objective response rates may be partially explained by the inability to give full monotherapy doses of each drug when they were combined. Additionally, molecular profiling was not routinely performed in clinical practice at the time this study was conducted. Therefore, patients were not selected according to tumor biomarkers and the genotype of tumors was for the most part unknown.

## Conclusions

It was not possible to combine the recommended phase II dose of selumetinib (75 mg BID) with standard doses of either erlotinib or temsirolimus. In patients with advanced solid tumors, the MTD of selumetinib was 100 mg QD in combination with erlotinib 100 mg QD, and 50 mg QD in combination with temsirolimus 25 mg once weekly. DLTs included diarrhea, mucositis, and neutropenia. Dose modifications were frequent even in patients who stayed on the study. Despite the promising preclinical work supporting dual pathway inhibition with MEK inhibitors in combination with either mTOR or EGFR inhibitors, translation to the clinic has been challenging due to overlapping AE profiles.

## Electronic supplementary material

ESM 1 (DOCX 84 kb)

ESM 2 (PDF 580 kb)
