# Erratum to: A phase I dose-escalation study of Selumetinib in combination with Erlotinib or Temsirolimus in patients with advanced solid tumors

**DOI:** 10.1007/s10637-017-0479-3

**Published:** 2017-07-05

**Authors:** Jeffrey R. Infante, Roger B. Cohen, Kevin B. Kim, Howard A. Burris, Gregory Curt, Ugochi Emeribe, Delyth Clemett, Helen K. Tomkinson, Patricia M. LoRusso

**Affiliations:** 1 Sarah Cannon Research Institute, 93 Harley St, Marylebone, London, W1G 6AD UK; 2 Tennessee Oncology, PLLC, 250 25th Ave North, Nashville, TN 37203 USA; 3 Fox Chase Cancer Center, 333 Cottman Avenue, Philadelphia, PA 19111 USA; 4 California PacificMedical Center (Sutterhealth), 475 Brannan Street, Suite 220, San Francisco, CA 94107 USA; 5 AstraZeneca, 1800 Concord Pike, Wilmington, DE 19850 USA; 6 AstraZeneca, Charter Way, Macclesfield, SK10 2NA UK; 7 Yale Cancer Center, 55 Park Street, Ste First Floor, New Haven, CT 06519 USA

**Erratum to: Invest New Drugs (2017)**

**DOI 10.1007/s10637-017-0459-7**

The article A phase I dose-escalation study of Selumetinib in combination with Erlotinib or Temsirolimus in patients with advanced solid tumors, written by Jeffrey R. Infante, Roger B. Cohen, Kevin B. Kim, Howard A. Burris III, Gregory Curt, Ugochi Emeribe, Delyth Clemett, Helen K. Tomkinson, and Patricia M. LoRusso, was originally published electronically on the publisher’s internet portal (currently SpringerLink) on April 19, 2017 without open access.

With the author(s)’ decision to opt for Open Choice the copyright of the article changed on July 03, 2017 to © The Author(s) 2017 and the article is forthwith distributed under the terms of the Creative Commons Attribution 4.0 International License (http://creativecommons.org/licenses/by/4.0/), which permits use, duplication, adaptation, distribution and reproduction in any medium or format, as long as you give appropriate credit to the original author(s) and the source, provide a link to the Creative Commons license and indicate if changes were made.

The original article has been corrected.

