# Supplementary material for: A phase I dose-escalation study of Selumetinib in combination with Erlotinib or Temsirolimus in patients with advanced solid tumors
Source: Invest New Drugs. 2017 Apr 19;35(5):576–88. doi: 10.1007/s10637-017-0459-7 (PMC5613062; doi:10.1007/s10637-017-0459-7)

A Phase I Dose-Escalation Study of Selumetinib in Combination with Erlotinib or Temsirolimus in Patients with Advanced Solid Tumours

Investigational New Drugs

Jeffrey R Infante^1,2^, Roger B Cohen^3^, Kevin B Kim^4^, Howard A Burris III^1,2^, Gregory Curt^5^^, Ugochi Emeribe^5^, Delyth Clemett^6*^, Helen K Tomkinson^6^, and Patricia M LoRusso^7^

*^1^Sarah Cannon Research Institute, London, United Kingdom*

*^2^Tennessee Oncology, PLLC, Nashville, Tennessee, United States of America*

*^3^Fox Chase Cancer Center, Philadelphia, PA, United States of America*

*^4^California Pacific Medical Center, San Francisco, California, United States of America*

*^5^AstraZeneca, Wilmington, Delaware, United States of America*

*^6^AstraZeneca, Macclesfield, United Kingdom*

*^7^Yale Cancer Center, New Haven, Connecticut, United States of America*

**Corresponding author:** Jeffrey R Infante

Phone: 615 340 2829. Email: jinfante@tnonc.com

# Supplementary material

**Supplementary table 1** Independent Ethics Committees/Institutional Review Boards consulted

| **Research site** | **Ethics committee** | **Committee number** |
| --- | --- | --- |
| Detroit, Michigan, USA | Wayne State University, Human Investigations Committee | 050508M1F |
| Austin, Texas, USA | IntegReview | IRB00001035, IRB00003657, IRB00004920, IRB00006075 |
| Rockledge, Pennsylvania, USA | Institutional Review Board, Fox Chase Cancer Canter | IRB #08-001 |
| Houston, Texas, USA | Institutional review Board, MD Anderson Cancer Center | IRB00000121 |

**Supplementary table 2** Summary of pharmacokinetic parameters of single-dose selumetinib following dosing alone and in combination with erlotinib

|  | **Geometric mean (% co-efficient of variation) [n evaluable]** | | | | | |
| --- | --- | --- | --- | --- | --- | --- |
| **Parameter** | **Selumetinib dosed alone** | | | **Selumetinib dosed in combination with erlotinib** | | |
|  | **50 mg  N = 7** | **100 mg  N = 6** | **150 mg  N = 13** | **50 mg  N = 6** | **100 mg  N = 6** | **150 mg  N = 12** |
| **Selumetinib** |  |  |  |  |  |  |
| C_max_, ng/mL | 683 | 1804 | 2447 | 942 | 2599 | 2811 |
| t_max_, h^a^ | 1.00 (1.00–2.00) | 1.50 (1.00–2.00) | 1.50 (1.00–2.00) | 1.00 (1.00–4.00) | 1.25 (1.00–1.50) | 1.00 (1.00–2.00) |
| AUC_(0–8)_, ng•h/mL | 1902 | 4403 | 6777 | 2600 | 7074 | 9189 |
| AUC_(0–12)_, ng•h/mL | 2131 | 4999 | 7741 | 3033 | 7762 | 10,530 |
| **N-desmethyl selumetinib** |  |  |  |  |  |  |
| C_max_, ng/mL | 45.6 | 109 | 79.6 | 44.5 | 129 | 68.3 |
| t_max_, h^a^ | 1.50 (1.00–4.00) | 1.50 (1.50–4.00) | 1.50 (1.00–8.00) | 1.50 (1.50–8.00) | 1.50 (1.00–2.00) | 2.00 (1.00–4.00) |
| AUC_(0–8)_, ng•h/mL | 156 | 347 | 279 | 149 | 404 | 286 |
| AUC_(0–12)_, ng•h/mL | 181 | 401 | 331 | 181 | 454 | 344 |
| **Erlotinib** |  |  |  |  |  |  |
| C_max_, ng/mL | 1620^b^ | 1180 | 1980^c^ | 1468^d^ | 1433 | 2031 |
| t_max_, h^a^ | 2.00(1.00–8.00)^b^ | 1.50 (1.00–4.00) | 2.00 (1.00–6.00)^c^ | 4.00 (1.00–8.00)^d^ | 2.00 (1.00–6.00) | 2.00 (1.00–8.00) |
| AUC_(0–8)_, ng•h/mL | 9624^b^ | 7100 | 12,710^c^ | 8600^d^ | 8496 | 13,000 |
| AUC_(0–12)_, ng•h/mL | 14,000^b^ | 10,330 | 18,320^c^ | 12,890^d^ | 11,990 | 18,580 |

Erlotinib, 100 mg orally QD.
^a^Median value and range.
^b^n = 8.
^c^n = 12.
^d^n = 7.
AUC_(0–8)_, area under the concentration-time curve from 0 to 8 hours; AUC_(0–12)_, area under the concentration-time curve from 0 to 12 hours; BID, twice daily; C_max_, maximum plasma concentration; QD, once daily; t_max_, time to reach maximum plasma concentration.

**Supplementary figure 1** Plots of geometric mean (+/- standard deviation) plasma concentrations over time of (a) selumetinib 75 mg alone and in combination with erlotinib 100 mg QD or (b) erlotinib alone 100 mg QD and in combination with selumetinib 75 mg BID. The selumetinib plasma concentration data at 12 hours from one patient were excluded from the mean result as it appeared that the blood sample was taken after the second daily dose. BID, twice daily; QD, once daily


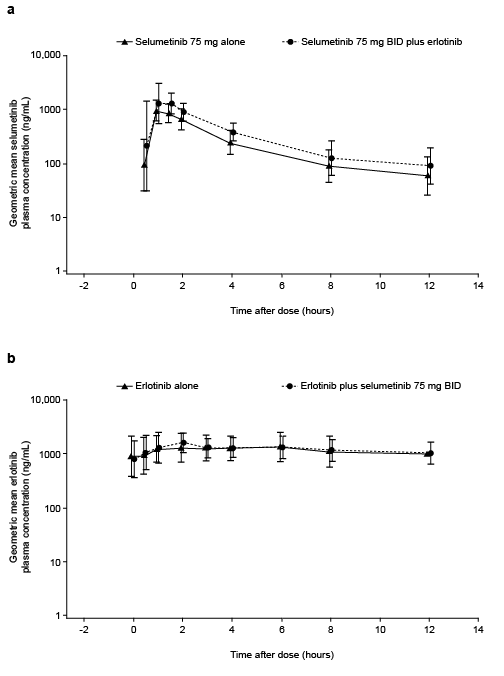


**Supplementary figure 2** Plots of geometric mean (+/- standard deviation) plasma concentrations over time of (a) selumetinib 75 mg alone and in combination with temsirolimus or (b) temsirolimus alone and in combination with selumetinib 75 mg BID. The selumetinib plasma concentration data at 12 hours from three patients were excluded from the mean result as it appeared that their blood samples were taken after the second daily dose. BID, twice daily


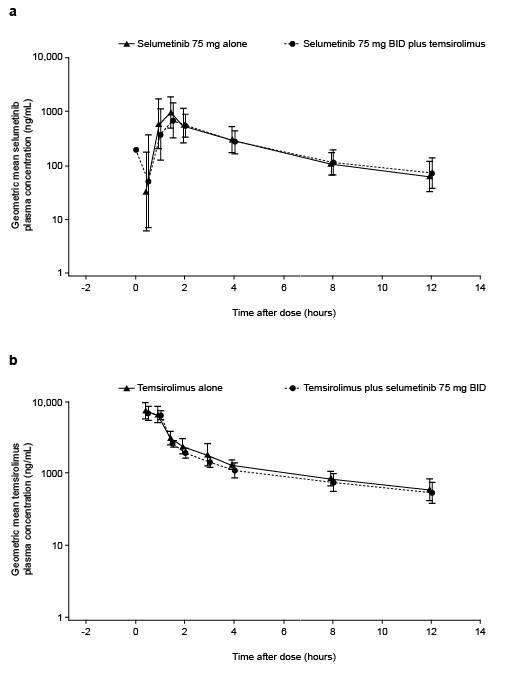

Supplement: Supplementary file 1 — (DOCX 84 kb) [file 10637_2017_459_MOESM1_ESM.docx]
