# Supplementary material for: A phase I dose-escalation study of Selumetinib in combination with Erlotinib or Temsirolimus in patients with advanced solid tumors
Source: Invest New Drugs. 2017 Apr 19;35(5):576–88. doi: 10.1007/s10637-017-0459-7 (PMC5613062; doi:10.1007/s10637-017-0459-7)
Supplement: Supplementary file 2 — (PDF 580 kb) [file 10637_2017_459_MOESM2_ESM.pdf]

AstraZeneca

Revised Clinical Pharmacology Study Protocol

Drug Substance	AZD6244 Hyd-Sulfate
Study Code	D1532C00004
Edition Number	6
Date	

A Phase I, Open-Label, Multi-center Study to Assess the Safety, Tolerability and Pharmacokinetics of AZD6244 Hyd-Sulfate When Given in Combination with Standard Doses of Selected Chemotherapies to Patients with Advanced Solid Tumors

Sponsor:

AstraZeneca Research and Development site representative ______________________ ______________________

Date

The following Amendment(s) and Administrative Changes have been made to this protocol since the date of preparation:

**Amendment No**	**Date of Amendment:**
No. 1	
No. 2	
No.3 MDACC Site Specific	
No. 4	
No. 5	
No. 6	

**Administrative Change No.**	**Date of Administrative Change**
No. 1	

This submission /document contains trade secrets and confidential commercial information, disclosure of which is prohibited without providing advance notice to AstraZeneca and opportunity to object.

ASTRAZENECA PROCEDURES IN CASE OF EMERGENCY, OVERDOSE OR PREGNANCY

In the case of a medical emergency you may contact the Study Delivery Team Leader. If the Study Delivery Team Leader is not available, contact the Study Delivery Team Physician at the AstraZeneca Research and Development site shown below.

Role in the study	Name	Address and Telephone number
Study Delivery Team Leader		
Study Delivery Team Physician		
Business hours emergency cover		8:00 AM – 7:00 PM EST<br>Tel:
After hours emergency cover		7:00 PM – 8:00 AM EST<br>Tel:

For further clarifications regarding:

- Procedures in case of medical emergency see Section 8.2
- Procedures in case of overdose see Section 8.3.
- Procedures in case of pregnancy see Section 8.4

PROTOCOL SYNOPSIS

A Phase I, Open-Label, Multi-center Study to Assess the Safety, Tolerability and Pharmacokinetics of AZD6244 Hyd-Sulfate When Given in Combination with Standard Doses of Selected Chemotherapies to Patients with Advanced Solid Tumors

Coordinating Investigator:

Other Study center(s), type and number of patients planned

The study will be conducted at 4 study centers in the United States. Approximately 140 patients will be enrolled; A minimum of 18 patients will be treated at the highest tolerated dose for each therapy combination.

**Study period**	**Phase of development**
Estimated date of first patient enrolled	I
Estimated date of last patient completed	

*The end of study is defined as 30 days after the last patient discontinues study treatment. However, final analysis will occur 6 months after the last patient recruited starts investigational product.

Objectives

Primary Objective:

To investigate the safety, tolerability and pharmacokinetics of twice daily oral doses of AZD6244 Hyd-Sulfate when administered in combination with the following chemotherapies:

- Docetaxel
- Dacarbazine
- Erlotinib
- Temsirolimus

Secondary objective:

- To define the highest tolerated dose of AZD6244 Hyd-Sulfate when administered in combination with standard doses of selected chemotherapies

Exploratory objectives:

- To make a preliminary assessment of tumor response as measured by Objective Response Rate (ORR) per investigator's assessment using Response Evaluation Criteria in Solid Tumors (RECIST) when AZD6244 Hyd-Sulfate is given in combination with standard doses of selected chemotherapies
- To analyze biological samples (e.g. archived tumor, plasma) for factors, which may influence the sensitivity to AZD6244 Hyd-Sulfate (or agents used in combination), such as genetic variability, gene expression profiling, protein expression profiling
- To collect a blood sample (optional) for DNA extraction and storage to provide data to investigate whether variability (if observed) in the PK, safety, efficacy or PD results could be explained by differences in the patient's genotype

Study design:

This is a phase I, open-label study to investigate the safety and tolerability of twice-daily oral doses of AZD6244 Hyd-Sulfate when administered in combination with standard doses of selected chemotherapies. Eligible patients are those with cancers who would be candidates for one of the following chemotherapies as a standard of care or those who may derive benefit from the combination therapies: (1) docetaxel, (2) dacarbazine(3) erlotinib, and (4) temsirolimus. In this protocol, the term chemotherapy will refer to all types of anti-cancer agents.

For each treatment arm, the study will be conducted in two parts: Part A (Dose escalation) and Part B (Dose expansion).

Part A Dose Escalation and Highest Tolerated Dose

Part A will define the highest tolerated dose of AZD6244 Hyd-Sulfate when administered twice daily with standard doses of selected chemotherapies. Treatments will be administered according to the standard dose and schedule for each and employ all prescribed ancillary and supportive care practices. Twice daily oral dosing of AZD6244 Hyd-Sulfate 50 mg will begin on Cycle 1/Day 3 of docetaxel and dacarbazine treatments.

Due to the long half lives of erlotinib (and requirement to dose to steady state) and temsirolimus (and the active metabolite sirolimus) in these arms the PK of AZD6244 Hyd-Sulfate alone will be assessed after a single dose administration prior to chemotherapy to prevent the need for a lengthy washout period. One single dose of AZD6244, the dose the patient will be receiving BD, will be given three to eight days prior to Cycle 1/Day 1 for the erlotinib and temsirolimus treatments. Twice daily oral dosing of AZD6244 will then begin on Cycle 1/Day 8 for erlotinib and temsirolimus. The starting dose of AZD6244 Hyd-Sulfate will be 50 mg BD composed of 25 mg capsules.

Patients will be enrolled into Part A in initial cohorts of 3 to 6. The dose of AZD6244 for the next cohort will depend on the tolerability of the starting dose, but predicted exposure will not exceed the exposures observed at the monotherapy MTD of 75 mg BD.

Part B Dose Expansion

Part B will enroll larger cohorts at the highest tolerated dose established in Part A for AZD6244 Hyd-Sulfate in combination with standard doses of selected chemotherapy. The highest tolerated dose will be defined in Part A as the highest investigated dose deemed to be well tolerated. Part B will confirm the dose of AZD6244 Hyd-Sulfate that can be combined with the selected standard chemotherapies.

Enrollment to the 4 chemotherapy +AZD6244 Hyd-Sulfate treatment groups will be in parallel and progress independently.

Target patient population

Male and female patients aged 18 years or older with advanced solid tumors and for whom the selected standard chemotherapy regimens represents a standard of care or those who may derive benefit from one of the combination therapies.

Investigational product, dosage and mode of administration

AZD6244 Hyd-Sulfate will be administered orally as 25 mg capsules. The AZD6244 Hyd-Sulfate formulation will be used in this study and unless otherwise specified, it is the formulation referenced throughout this document.

Duration of treatment

In all arms of the study, following at least 28 days of therapy from Cycle 1/Day 1, patients may continue to receive AZD6244 in combination with chemotherapy, or as a single agent until disease progression occurs or as long as they do not experience intolerable toxicity and

the investigator believes they are continuing to derive benefit from the therapy. If AZD6244 therapy must be permanently discontinued, patients must be withdrawn from the study.

For docetaxel and dacarbazine, AZD6244 Hyd-Sulfate will be initiated on day 3 of the first cycle and will be given continuously on a twice-daily basis thereafter. For erlotinib and temsirolimus, a single dose of AZD6244 Hyd-Sulfate will be given three to eight days prior to Cycle 1/Day 1 and then will be given continuously on a twice-daily basis starting on Cycle 1/Day 8.

- Docetaxel: 75 mg/m^2 IV infusion over 60 minutes on day 1, of each 21 day cycle.
- Dacarbazine: 1000 mg/ m^2 IV infusion over 60 minutes on day 1, of each 21 day cycle.
- Erlotinib: 100 mg oral daily continuously starting on Cycle 1/Day 1
- Temsirolimus: 25 mg IV infusion over 60 minutes on days 1, 8, and 15 of each 21 day cycle.

The temsirolimus dose may be reduced to 15mg if the DLT(s) observed for the cohort is/are a known side effect of temsirolimus. Likewise, if the DLT(s) is/are a known side effect of AZD6244, than the AZD6244 dose will be reduced accordingly. All dosing decisions will be at the discretion of the SRC who will review the emerging tolerability and safety profile on an ongoing basis, and on completion of each cohort dose level, and decide whether to continue at 25mg of temsirolimus or reduce to 15 mg for subsequent cohorts.

For the purpose of the study plans in this protocol, a cycle is defined as 21 days. A 21 day cycle was selected for consistency across all arms of the protocol.

Outcome variables

Safety

Incidence and intensity of adverse events as graded by Common Terminology Criteria for Adverse Events (CTCAE Version 3.0). Safety will also be monitored using physical examinations, vital signs (including weight, blood pressure and pulse rate), ECG parameters, MUGA scans or echocardiography, clinical chemistry (including liver function tests), Brain Natriuretic Peptide (BNP), Troponin I, hematology, urinalysis, and ophthalmologic examinations.

Pharmacokinetics

Where the data allow, the following PK parameters will be determined following administration of chemotherapy alone, following administration of AZD6244 alone and following administration of AZD6244 and chemotherapy together (the term alone refers to days when either AZD6244 or chemotherapy is administered, the term together refers to days when both AZD6244 and chemotherapy are administered on the same day). Additional parameters may be determined if deemed appropriate.

- AZD6244: C_{max}, t_{max}, $AUC_{(0-12)}$
- N-desmethyl AZD6244: C_{max}, t_{max}, $AUC_{(0-12)}$
- Chemotherapies: C_{max}, t_{max}, $AUC_{(0-12)}$

Efficacy

Preliminary assessment of tumor response by Objective Response Rate (ORR) per investigator’s assessment using RECIST when AZD6244 is given in combination with standard doses of selected chemotherapies.

Statistical methods

The study is not formally powered for efficacy but is designed to provide adequate tolerability, safety, and pharmacokinetic data and to ensure at least 18 evaluable patients are recruited at the dose deemed to be the highest tolerated dose of AZD6244 in combination with each chemotherapy regimen.

The expansion at the highest tolerated dose in Part B provides further safety data for evaluating the MTD.

TABLE OF CONTENTS PAGE

TITLE PAGE 1
PROTOCOL SYNOPSIS 3
TABLE OF CONTENTS 8
LIST OF ABBREVIATIONS AND DEFINITION OF TERMS 14
1. INTRODUCTION 18
1.1 Background 18
1.1.1 Mitogen activated protein kinase (MEK) 18
1.1.2 Pre-clinical experience with AZD6244 18
1.1.3 Clinical experience with AZD6244 21
1.2 Rationale 24
2. STUDY OBJECTIVES 24
2.1 Primary objectives 24
2.2 Secondary objective 25
2.3 Exploratory objectives 25
3. STUDY PLAN AND PROCEDURES 25
3.1 Overall study design 25
3.1.1 Part A dose escalation 26
3.1.2 Part B dose expansion 27
3.1.3 Definition of Dose Limiting Toxicity 29
3.2 Study Procedures 30
3.2.1 Management of Toxicities for AZD6244 32
3.2.1.1 Rash Management Guidance 33
3.2.2 Dose Reductions of Standard Chemotherapy 33
3.2.3 Study plans and PK tables 40
3.3 Rationale and risk/benefit assessment 58
3.3.1 Rationale for study design, doses and control groups 58
3.3.2 Risk/benefit and ethical assessment 59
3.4 Selection of study population 61
3.4.1 Study selection record 61
3.4.2 Inclusion criteria 62
3.4.3 Exclusion criteria 62
3.4.4 Protocol precautions 64
3.4.5 Restrictions 64
3.4.6 Discontinuation of patients from treatment or assessment 65
3.4.6.1 Criteria for discontinuation 65

3.4.6.2 Withdrawal of consent for optional biomarker and/or host genetics research 66
3.4.6.3 Procedures for discontinuation from treatment or assessment 66
3.4.6.4 Procedures for discontinuation from optional biomarker and/or host genetic aspects of study 67
3.4.6.5 Procedures for handling incorrectly enrolled patients 67

3.5 Treatment(s) 67
3.5.1 Investigational product(s) 67
3.5.1.1 Identity of investigational product 67
3.5.1.2 Labeling 68
3.5.1.3 Storage 68
3.5.1.4 Accountability 68
3.5.2 Doses and treatment regimens 68
3.5.2.1 Capsule formulation 68
3.5.2.2 AZD6244 Dosing 69
3.5.2.3 Dosing of standard chemotherapy either alone or in combination with AZD6244 69
3.5.3 Method of assigning patients to treatment groups 70
3.5.4 Blinding and procedures for unblinding the study 70
3.5.5 Concomitant medication 70
3.5.6 Treatment compliance 70

4. MEASUREMENT OF STUDY VARIABLES 71

4.1 Medical examination and demographic measurements 71
4.1.1 Enrollment medical examination and demographic measurements 71
4.1.2 Post-study medical examination 72

4.2 Pharmacokinetic measurements 73
4.2.1 Determination of drug concentration in biological samples 73
4.2.2 Collection of biological samples 73

4.3 Pharmacodynamic measurements 74
4.3.1 Collection of archived tumor and plasma samples for biomarker analysis 74

4.4 Safety measurements 74
4.4.1 Laboratory safety measurements 74
4.4.2 Pregnancy test 75
4.4.3 Electrocardiographic measurements 75
4.4.4 Physical examination 76
4.4.5 Vital signs 76
4.4.5.1 Blood pressure and pulse rate 76
4.4.6 Other safety measurements 76
4.4.6.1 MUGA scan/echocardiogram 76
4.4.6.2 BNP 76
4.4.6.3 Troponin I 76
4.4.6.4 O_2 saturation measurement 77
4.4.6.5 Chest X-ray 77

4.4.6.6 Ophthalmologic examination ... 77
4.4.6.7 A complete ophthalmologic examination including visual acuity, visual fields, color vision, and a slit-lamp examination must be performed ≤ 6 weeks prior to Cycle 1/Day 1 (docetaxel and dacarbazine) and six weeks prior to first dose of AZD6244 (erlotinib and temsirolimus), at week 6, if the patient experiences a visual disturbance AE and at discontinuation from AZD6244. Results must be recorded in the CRF.HRCT ... 77

4.5 Efficacy measurements and co-variables ... 77
4.5.1 Tumor assessment by imaging techniques using RECIST for patients with measurable disease at baseline ... 77
4.5.2 Objective response rate ... 79
4.5.2.1 Objective response rate and method of assessment ... 79
4.5.3 Tumor assessments for patients with non-measurable disease at baseline ... 79
4.5.3.1 Methods of assessment ... 79

4.6 Genetic measurements and co-variables ... 79
4.6.1 Collection of samples for genetic testing ... 79

4.7 Volume of blood sampling ... 80

4.8 Adverse Events ... 81
4.8.1 Adverse Events ... 81
4.8.1.1 Definitions ... 81
4.8.1.2 Recording of adverse events ... 82
4.8.1.3 Reporting of serious adverse events ... 89

5. STUDY MANAGEMENT ... 90

5.1 Monitoring ... 90
5.1.1 Study monitoring ... 90
5.1.2 Data verification ... 90

5.2 Audits and inspections ... 91

5.3 Training of staff ... 91

5.4 Changes to the protocol ... 91

5.5 Study agreements ... 92

5.6 Study timetable and end of study ... 92

5.7 Data management ... 92
5.7.1 Case report forms ... 92

6. PHARMACOKINETIC, PHARMACODYNAMIC, SAFETY, BIOMARKER AND STATISTICAL METHODOLOGY ... 93

6.1 Pharmacokinetic / pharmacodynamic evaluation ... 93
6.1.1 Calculation or derivation of pharmacokinetic variables ... 93
6.1.2 Calculation or derivation of pharmacodynamic variables ... 94
6.1.3 Calculation or derivation of pharmacokinetics/pharmacodynamics ... 94

6.2 Safety evaluation 94
6.2.1 Calculation or derivation of safety variables 94
6.3 Biomarker and Genetic markers as a co-variable 94
6.4 Statistical methods and determination of sample size 94
6.4.1 Statistical evaluation 94
6.4.2 Description of outcome variables in relation to objectives and hypotheses 94
6.4.3 Description of analyzes sets 95
6.4.4 Method of statistical analysis 95
6.4.5 Determination of sample size 95
6.5 Interim analyzes 96
6.6 Data presentation 96
6.6.1 Safety Data 96
6.6.2 PK data 97
6.6.3 Tumor response data 97
6.6.4 Pharmacogenetic data 97
6.7 Reporting of Genotypic Results (pharmacogenetics) 97
6.8 Data monitoring committee 97
6.8.1 Study Safety Review Committee 98
7. ETHICS 98
7.1 Ethics review 98
7.2 Ethical conduct of the study 99
7.3 Informed Consent 99
7.4 Patient data protection 99
8. PROCEDURES IN CASE OF EMERGENCY, OVERDOSE OR PREGNANCY 100
8.1 AstraZeneca emergency contact procedure 100
8.2 Procedures in case of medical emergency 100
8.3 Procedures in case of overdose 100
8.4 Procedures in case of pregnancy 101
9. REFERENCES 102

LIST OF TABLES PAGE

Table 1 In vivo combination effects of AZD6244..19
Table 2 Planned Dose Escalation Schedule for AZD6244....................................26
Table 3 Study plan: Docetaxel..40
Table 4 PK sample collection docetaxel and AZD6244..43
Table 5 Study plan: Dacarbazine..44
Table 6 PK sample collection dacarbazine and AZD6244....................................47
Table 7 Study plan: Erlotinib..48
Table 8 PK sample collection Erlotinib and AZD6244...51
Table 9 Study plan: Temsirolimus..53
Table 10 PK sample collection Temsirolimus and AZD6244................................56
Table 11 Identity of investigational product..67
Table 12 Clinical chemistry, hematology, and urinalysis 74
Table 13 Volume of blood to be drawn from each patient – Part A & B[a]..............80
Table 14 Description of analyzes sets 95

LIST OF FIGURES PAGE

Figure 1 Study flow chart 39
Figure 2 Dyspnea Treatment Algorithm...87
Figure 3 LVEF Algorithm 88

LIST OF APPENDICES

Appendix A Not Applicable

Appendix B Additional Safety Information

Appendix C Definitions of measurable, non-measurable, target and non-target lesions and treatment evaluation response based on the RECIST (Response Evaluation Criteria in Solid Tumors) criteria

Appendix D Optional Genetics Research

Appendix E Optional Biomarker Research

Appendix F Docetaxel label

Appendix G Dacarbazine label

Appendix H Erlotinib label

Appendix I Temsirolimus label

Appendix J Medications known or suspected to prolong QT interval

LIST OF ABBREVIATIONS AND DEFINITION OF TERMS

The following abbreviations and special terms are used in this study protocol.

Abbreviation or special term	Explanation
AE	Adverse event (see definition in Section 4.8.1.1)
AJCC	American Joint Committee on Cancer
ALT	Alanine aminotransferase
ANC	Absolute neutrophil count
AP	Alkaline phosphatase
ASCO	American Society of Clinical Oncology
AST	Aspartate aminotransferase
AUC	Area under the plasma concentration-time curve
$AUC_{(0-t)}$	Area under the plasma concentration-time curve from zero to time of the last quantifiable concentration
$AUC_{(0-12)}$	Area under the plasma concentration-time curve from zero to 12 hours post dose
AUC_{ss}	Area under the plasma profiles at steady-state
AUMC	Area under the first moment curve
BCRP	Breast cancer resistance protein
BD	Twice daily dosing
BNP	Brain natriuretic peptide
BOR	Best overall response
BP	Blood pressure
BRAF	v-raf murine sarcoma viral oncogene homolog B1
BSA	Body surface area
BSR	Blasticidin S-resistance gene
CBC	Complete blood count
CI	Confidence interval
C_{max}	Maximum plasma concentration
$C_{max\ ss}$	Maximum plasma concentration at steady state
$C_{min\ ss}$	Minimum plasma concentration at steady state
CNS	Central nervous system
CR	Complete response

**Abbreviation or special term**	**Explanation**
CRF/pCRF	(paper) Case Report Form
CT	Computed Tomography
CTCAE	Common Terminology Criteria for Adverse Events
CL/F	Apparent oral clearance
CV	Coefficient of variation
DBL	Database lock
DLT	Dose Limiting Toxicity
DMPK	Drug Metabolism and Pharmacokinetics
DOB	Date of birth
E code	Enrollment code
ECG	Electrocardiogram
ECOG	Eastern Cooperative Oncology Group
EF	Ejection fraction
ERK	Extracellular signal-regulated kinase
Ethics Committee	Synonymous to Institutional Review Board and Independent Ethics Committee
gmean	Geometric Mean
GCP	Good Clinical Practice
GI	Gastrointestinal
GMP	Good manufacturing practice
HDPE	High density polyethylene
Hgb	Hemoglobin
HIV	Human immunodeficiency virus
HRCT	High resolution CT
IB	Investigator Brochure
ICH	International Conference on Harmonisation
IC_{50}	Inhibitory concentration ie, concentration of compound required to give 50% inhibition of biological effect
INR	International normalized ratio
IPS	Investigational Product Supplies
IRB	Institutional Review Board
IUD	Intrauterine device

Abbreviation or special term	Explanation
IV	Intravenous
Ki-67	Proliferative index
LOQ	Limit of quantification
LV	Leucovorin
MAP	Mitogen-Acitvated Protein
MAPKK	Mitogen-Acitvated Protein Kinase Kinase
MedDRA	Medical dictionary for regulatory activities
MEK	Mitogen-Activated Protein Kinase Kinase (also known as MAPKK)
mg	Milligrams
MIE	Micronucleated immature erythrocytes
MRI	Magnetic Resonance Imaging
MRT	Mean residence time
MUGA	Multiple gated acquisition scan
MTD	Maximum Tolerated Dose
nM	Nanomolar
NQ	Non-quantifiable
NRAS	Neuroblastoma RAS viral (v-ras) oncogene homolog
NSAID	Non-steroidal anti-inflammary drug
NT-proBNP	N-terminal prohormone b-type natriuretic peptide
O_2	Oxygen
OAE	Other Significant Adverse Event
od	Once daily dosing
ORR	Objective Response Rate
pERK	Phosphorylated Extracellular signal-regulated kinase
PD	Pharmacodynamic
PFT	Pulmonary function test
PgP	p-glycoprotein
PI	Package insert
PK	Pharmacokinetics
PR	Partial response
PT	Preferred term
QT	Time-interval between start of Q-wave and end of T-wave in an ECG

**Abbreviation or special term**	**Explanation**
QTc	QT corrected for pulse rate
RECIST	Response evaluation criteria in solid tumors
SAE	Serious adverse event (see definition in Section 4.8.1).
SAP	Statistical Analysis Plan
SBE-CD	sulphobutylether β-cyclodextrin (Captisol®)
SD	Stable Disease
SOC	System organ class
SOP	Standard operating procedure
SRC	Safety Review Committee
t_{max}	Time to reach maximum plasma concentration
$t_{max\ ss}$	Time to reach maximum plasma concentration at steady state
tx	Treatment
$t_{1/2}$	Half life
$t_{½}\lambda_z$	Terminal elimination rate constant
ULN	Upper limit of normal
UV	Ultra Violet
γGT	Gamma glutamyltranserase
VEGF	Vascular endothelial growth factor
V_{ss}	Volume of distribution at steady state
WHO	World Health Organization

1. INTRODUCTION

For more detailed pre-clinical experience and clinical experience with AZD6244, please refer to the Investigator Brochure (IB).

1.1 Background

1.1.1 Mitogen activated protein kinase (MEK)

Human cancers often arise as a result of mutations in cellular signalling pathways that coordinate and regulate cell proliferation and survival. The intracellular Ras regulated RAF/MEK/ERK protein kinase signal cascade is a key pathway involved in cellular proliferation and there is a strong link between deregulation of this pathway and uncontrolled cell proliferation and survival Kolch 2000). The RAF/MEK/ERK kinase cascade holds a central coordinating role in mediating signal transduction from extra cellular growth factors, cytokines and proto-oncogenes. Some tumors contain mutations that result in the continuous activation of the pathway due to continuous production of growth factors, or express mutated or increased levels of growth factor receptors. Within this central cascade, mitogen activated protein kinase kinase (MEK) has been identified as an attractive therapeutic target because the only known substrates for MEK phosphorylation are the mitogen activated protein (MAP) kinases, extracellular signal regulated kinase ERK1 and ERK2.

It is anticipated that inhibition of MEK activity should inhibit transduction of the mitogenic signals from multiple pathways, resulting in an effect on tumor proliferation, differentiation and survival. Given the widespread distribution of constitutive ERK activation, a MEK inhibitor could be anticipated to have a broad spectrum of clinical utility in both solid tumors and hematological malignancies.

AZD6244 is a potent, selective, uncompetitive inhibitor of MEK, licensed for development by AstraZeneca Pharmaceuticals from Array BioPharma. Array BioPharma was responsible for the first administration into man study, performed at three centers in the United States. The remainder of the clinical development program for oncology indications is the responsibility of AstraZeneca. Four monotherapy Phase II studies commenced in 2006.

AZD6244 pre-clinical information is summarized in Section 1.1.2 of this protocol, and further information on pre-clinical toxicology and pharmacology may be found in the IB.

1.1.2 Pre-clinical experience with AZD6244

In vitro studies with enzyme assays have demonstrated that AZD6244 inhibits the activity of isolated MEK with an IC_{50} of approximately 10 nM to 14 nM. In contrast to its activity against MEK, AZD6244 was inactive, or only minimally active, against a panel of other kinases.

AZD6244 was particularly potent in inhibiting the cell viability of cell lines with the BRAF V600E gene mutation that leads to a constitutively active BRAF and, consequently, an

overactive MEK. AZD6244 was also a similarly potent inhibitor of some cell lines with activating KRAS gene mutations. In vivo pharmacology studies demonstrated activity of AZD6244 in tumor-bearing animals including tumor growth inhibition and regression of established tumors. Inhibition of ERK phosphorylation was found in tumors in which growth was inhibited by AZD6244. N-desmethyl AZD6244 (a pharmacologically active metabolite) was identified to be approximately 3-fold more active than the AZD6244 parent compound with respect to inhibition of ERK phosphorylation in cultured cells, and approximately 5-fold more active with respect to inhibition of cell viability. In vivo studies in human cancer xenografts have demonstrated the potential for AZD6244 to be used in combination with a number of cytotoxic and targeted agents, including docetaxel, irinotecan, gemcitabine, Iressa (gefitinib) and AZD2171 (cediranib).

AZD6244 has been combined with a number of therapeutic agents, including standard cytotoxic drugs, and novel targeted therapies using human xenograft models.

Table 1 In vivo combination effects of AZD6244

Xenograft Model	Treatment[a]	Result (Tumor growth inhibition as a % of control)
SW620 human colorectal cancer xenograft	AZD6244 25 mg/kg BD	74
	Irinotecan 25 mg/kg (once weekly)	67
	AZD6244 + irinotecan	93
	Docetaxel 15 mg/kg (once weekly)	12
	AZD6244 + docetaxel	98
HCT-116 human colorectal cancer xenograft	AZD6244 25 m/kg BD	64.9
	Docetaxel 15 mg/kg (once weekly)	93.4
	AZD6244 + docetaxel	123.2
Calu-6 human non-small cell lung cancer xenograft	AZD6244 3 mg/kg BD	48.8
	AZD2171 1.5 mg/kg QD	49.6
	AZD6244 + AZD2171	76.9
Lovo colorectal cancer xenograft	AZD6244 25 mg/kg BD	40.2
	Iressa 75 mg/kg QD	34.5
	AZD6244 + Iressa	75.1
SW620 colorectal cancer xenograft	Temozolamide (metabolized to the same active component as dacarbazine)	15
	AZD6244	58
	AZD6244 + Temozolamide	104
Lovo colorectal cancer xenograft	Rapamycin	38
	AZD6244	39
	AZ6244 + Rapamycin	63

[a]AZD6244 and the other therapeutic agents were administered according to their usual monotherapy schedules.

In vitro, AZD6244 underwent Phase I metabolic reactions including N-demethylation, oxidative defluorination, and loss of the side chain, to form amide and acid metabolites. Glucuronides of AZD6244 were detected in human hepatocyte incubations and plasma, indicating that direct conjugation is a significant route of elimination for AZD6244.

Preliminary in vitro data in human hepatocytes indicate thatAZD6244 was a weak inducer of cytochrome P450 (CYP)3A, 1A and 2C. AZD6244 was metabolized by CYP1A2 to produce N-desmethyl-AZD6244. Using expressed CYP isoforms, it was evident that AZD6244 was also metabolized by CYP 2C19 and CYP 3A4. AZD6244 showed no inhibition of the CYP isoforms 1A2, 2C8, 2C19, 2D6 or 3A4 at concentrations up to 50uM. It was a weak direct inhibitor of CYP2C9, with an IC_{50} of 44.7uM. N-desmethyl AZD6244 showed weak inhibition of CYP1A2, with an IC_{50} of 18.9uM.

AZD6244 free-base has limited solubility and exhibited a less-than-proportional increase in exposure and decreasing bioavailability with increasing dose in rat and monkey. However, enhanced and dose-related increases in exposure were seen in the mouse and monkey with AZD6244. There was no/minimal accumulation of AZD6244 (whether dosed as free-base or Hyd-Sulfate) on multiple dosing in mouse, rat and monkey.

AZD6244 absorbs in the Ultra Violet (UV) range for phototoxicity and shows enhanced cytotoxicity in the presence of UV light in an in-vitro 3T3 Neutral Red Uptake phototoxicity test. In rat, AZD6244 related material was widely distributed throughout tissues, including skin, eye and uveal tract, but did not show evidence of melanin binding. In rat and mouse AZD6244-related material was widely distributed, although tissue concentrations tended to be lower than blood concentrations. There was no evidence of binding to melanin, and penetration into the CNS was minimal. Protein binding was high: 99.7% in rat, 98.9% in mouse, 98.4% in human, 97.7% in monkey, 94.6% in dog, and 93.7% in Minipig. AZD6244 is a substrate but not an inhibitor of p-glycoprotein (PgP) and breast cancer resistance protein (BCRP).

Daily oral administration of AZD6244 free-base in SBE-CD for 1 month was well tolerated in rats, but produced soft stools and gastrointestinal mucosal mineralization. Tissue mineralization was not apparent in cynomolgus monkeys dosed for up to one month with AZD6244 free-base or AZD6244 Hyd-Sulfate. However, mineralization was seen in multiple tissues including cornea, kidney, liver, myocardium, skeletal muscle and glandular stomach in mice dosed with AZD6244 Hyd-Sulfate for up to 1 month. In mice and rats, tissue mineralization was associated with changes in plasma inorganic phosphate and albumin, and in mice, with changes in calcium. Twice-daily oral dosing of AZD6244 Hyd-Sulfate in SBE-CD produced fluid and/or red colored feces in monkeys, but with no notable gastrointestinal tract or renal pathology. In mice, BD oral dosing with AZD6244 Hyd-Sulfate at non-tolerated dose levels was associated with gastrointestinal tract toxicity and hematopoietic atrophy. Anemia, with an associated reticulocytosis, was also apparent in mice dosed with AZD6244 Hyd-Sulfate. In the one-month studies with AZD6244 (free-base and Hyd-Sulfate), there was

evidence of reversibility of most changes. However, in rats and mice, tissue mineralization was observed in several animals at the end of the recovery period.

Six month oral toxicity studies with AZD6244 Hyd-Sulfate in mice and monkeys are currently ongoing. In the mouse study, dosing of AZD6244 Hyd-Sulfate to male mice for 8–15 weeks at the high dose level produced dilation of the corpus spongiosum, leading to urethral compression and backward flow blockage resulting in dilation of seminal vesicles and bladder, but with no histopathological changes in either organ. The mechanism underlying these changes in the mice is unknown at this stage. There have been no indications of similar effects following dosing of AZD6244 Hyd-Sulfate for 6 months in cynomolgus monkeys. Other changes in the mouse 6-month study, including changes suggestive of gastrointestinal tract toxicity, tissue mineralization and hematological effects, are consistent with those seen previously in mice dosed for up to 1 month.

AZD6244 showed no evidence of mutagenic or clastogenic potential in vitro, but produced an increase in micronucleated immature erythrocytes (MIE) in mouse micronucleus studies, predominantly via an aneugenic mode of action. Preliminary reproductive toxicology data indicate that AZD6244 can have adverse effects on embryofetal development and survival at dose levels that do not induce maternal toxicity in mice.

1.1.3 Clinical experience with AZD6244

One Phase I study (Study # ARRY-0401) of the mix and drink formulation containing AZD6244 freebase has now completed. Two Phase II studies are complete and two are currently ongoing with AZD6244 free-base and an additional Phase I study is ongoing with AZD6244 Hyd-Sulfate (capsule formulation).

Study # ARRY-0401 is a Phase I, open-label, multi-center study sponsored by , to assess the safety and tolerability of AZD6244 free-base. Patients in this study had advanced solid malignant tumors and had either failed standard therapy, or no standard therapy exists. A total of 57 patients received at least 1 dose of AZD6244 in the dose-escalation (Part A) or cohort-expansion (Part B) parts of this study.

Key findings for this study are as follows:

- 100 mg BD of AZD6244 free-base was determined to be the MTD for the free-base mix and drink formulation, and this dose was selected for all ongoing Phase II studies with this formulation.

- AZD6244 was absorbed relatively quickly, with maximum AZD6244 concentrations (C_{max}) attained approximately 1 hour post-dose (median). Plasma N-desmethyl AZD6244 concentrations/exposure followed a similar pattern to AZD6244 over time, but appeared to be approximately 10% or less of AZD6244 concentrations/exposure in Part B of the study.

- Following C_{max}, AZD6244 concentrations appeared to decrease multi-exponentially. The terminal elimination phase appeared to start at approximately 4 to 8 hours post-dose and the half-life ($t_{½}$) is estimated to be approximately 11 hours (median).

- There is a large overlap in AZD6244 concentrations (C_{max}) and systemic exposure (area under the plasma concentration-time curve [AUC] on Day 1 or AUC from time 0 to 12 hours [$AUC_{(0-12)}$] on Day 15) across the 50 to 300 mg dose range. AZD6244 exposures appear to be of similar magnitude on Day 1 and Day 15, which suggests that AZD6244 might display time-independent kinetics.

- Target knockdown of tumor pERK was seen with 100 mg BD dosing of AZD6244 free-base, with a geometric mean (gmean) inhibition of 81% (90% confidence interval [CI] 50% to 93%) at Day 15, compared with pre-dose. In addition, a reduction in Ki67 (a nuclear antigen associated with cell proliferation) was seen in 9/20 patients dosed at 100 mg BD, with a reduction ≥50% of pre-dose seen in 5 patients.

- Objective tumor response was evaluated using the Response Evaluation Criteria In Solid Tumors (RECIST). Data indicate that the best overall response in this study was stable disease (SD). Of the patients who had at least 1 pre- and post-dose measurement of target lesions recorded as of 30 September 2006, SD was observed in 17 patients. Long term (≥5 months) SD was observed in 9 patients.

The program of Phase II AZD6244 monotherapy studies is partly complete; 2 studies completed, 2 ongoing. These four Phase II studies used the mix and drink formulation containing AZD6244 freebase.

- American Joint Committee on Cancer (AJCC) unresectable Stage 3 or 4 malignant melanoma (AZD6244 versus temozolomide; Study D1532C00003)

- Advanced pancreatic cancer (AZD6244 versus capecitabine; Study D1532C00008)

- Colorectal cancer (AZD6244 versus capecitabine; Study D1532C00011)

- NSCLC (AZD6244 versus pemetrexed; Study D1532C00012).

In study D1532C00003 (melanoma) there was no apparent difference in efficacy between AZD6244 and temozolomide for the primary endpoint (Progression Free Survival), although anti-tumor activity was detected. Neither study D1532C00011 (CRC) or study D1532C00012 (NSCLC) reached their primary endpoint of delaying disease progression versus the randomized comparators in the overall population, however, there was evidence of anti-tumor activity in the NSCLC study. Study D1532C00008 is currently ongoing and results are not available.

The key safety findings for 100 mg BD AZD6244 free-base, based on preliminary data from all clinical studies described above, and are as follows:

- The most frequently reported specific adverse events with 100 mg BD AZD6244 free-base (irrespective of assessed causality) by Medical Dictionary for Regulatory Activities (MedDRA) preferred term were diarrhea, dermatitis acneiform, nausea, vomiting, fatigue and peripheral edema.

- Other adverse events seen in the clinical program for which clinical monitoring is currently advised are: dyspnea, hypoxia, visual function disturbance and hypertension.

- There have been no reports of clinical changes suggestive of the corpus spongiosum changes, such as priapism (persistent penile erection), in any Phase I or II study with patients receiving AZD6244.

- There is a trend towards elevated liver transaminases in patients who received BD dosing of AZD6244 free-base. The majority of transaminase elevations reported either remained within normal limits or increased by no more than a single CTCAE grade. No similar increases are observed with bilirubin, indicating no obstructive process ongoing in the liver. There were no other reports of changes in laboratory parameters that were considered to be of clinical relevance.

- There is currently no signal for QTc prolongation with either formulation of AZD6244.

Study D1532C00005 is a Phase I, open-label study to assess the safety, tolerability and pharmacokinetics of AZD6244 Hyd-Sulfate in patients with advanced solid tumors. This study will also compare the relative bioavailability of the 2 formulations of AZD6244. Cohorts of patients have completed assessment at doses of up to 100 mg BD AZD6244 Hyd-Sulfate (capsule formulation), and recruitment to both the dose escalation phase and the relative bioavailability phase of the study are now complete. The key findings for this study are as follows:

- One DLT was reported in the first 22 days of dosing in the 75 mg BD cohort, CTCAE Grade 3 fatigue which resolved on discontinuation of AZD6244.

- Two DLTs were reported in the first 22 days of dosing in the 100mg BD cohort, pleural effusion and rash (both CTC AE Grade 3).

- The 75 mg BD dose has been identified as the maximum tolerated dose in study D1532C00005.

- The expansion phase of part B (relative bioavailability) is ongoing with 28 patients recruited to receive continuous dosing with 75 mg BD AZD6244 Hyd-Sulfate.

- Preliminary assessment of the emerging safety and tolerability profile of the patients receiving AZD6244 Hyd-Sulfate at all doses indicates that it is broadly

consistent with observations for the mix and drink formulation containing AZD624 freebase.

- No efficacy data are currently available from this study.

Further information on the pre-clinical and clinical findings to date can be found in the IB.

1.2 Rationale

AZD6244 has been combined with a number of therapeutic agents, including standard cytotoxic drugs and novel targeted therapies using human xenograft models of a number of different tumor types. In some cases, as shown in Table 1, a beneficial effect of the combination was observed when compared with the monotherapy.

The combination of AZD6244 with each of docetaxel, erlotinib and temsirolimus is being investigated in this study as data from preclinical studies into anti-tumor effects of the combination of AZD6244 with the same agent (docetaxel) or same pharmacological targets (gefitinib and rapamycin respectively) suggests an increased anti-tumor activity with the combination when compared to either agent alone.

The dacarbazine combination has been selected to investigate the potential additive effect of dacarbazine, a standard of care chemotherapy for advanced melanoma and AZD6244 which, as monotherapy, has also demonstrated some anti-tumor activity in advanced melanoma. AZD6244 is a targeted agent, a MEK inhibitor, and the hypothesis is that the efficacy of the two agents in combination will be at least additive.

Based on current information, it is reasonable to believe that AZD6244 in combination with docetaxel, dacarbazine, erlotinib, and temsirolimus will have toxicities that are tolerable/acceptable, have minimal anticipated PK interaction, and have sufficient clinical benefit to patients to support the continued development of the compound.

This study is therefore designed to investigate the BD dosing of AZD6244, a MEK inhibitor, in combination with standard chemotherapies in patients with advanced solid tumors. This study will evaluate the safety profile, tolerability, and PK of AZD6244 in combination with 4 standard chemotherapies and support subsequent testing in Phase II.

2. STUDY OBJECTIVES

2.1 Primary objectives

To investigate the safety , tolerability and pharmacokinetics of twice daily oral doses of AZD6244 when administered in combination with the following chemotherapies:

- Docetaxel

- Dacarbazine

- Erlotinib
- Temsirolimus

The primary outcome variables to be collected are safety variables: Incidence and intensity of adverse events as graded by CTCAE (version 3.0), physical examinations, vital signs (including weight, blood pressure and pulse rate), ECG parameters, MUGA scan and echocardiography, clinical chemistry (including liver function tests), Brain Natriuretic Peptide (BNP), Troponin I, hematology, urinalysis, and ophthalmologic examinations.

2.2 Secondary objective

- To define the highest tolerated dose of AZD6244 when administered in combination with standard doses of selected chemotherapies

2.3 Exploratory objectives

- To make a preliminary assessment of tumor response as measured by Objective Response Rate (ORR) per investigators assessment using RECIST when AZD6244 is given in combination with standard doses of selected chemotherapies
- To analyze biological samples (e.g. archived tumor, plasma) for factors, which may influence the sensitivity to AZD6244 (or agents used in combination), such as genetic variability, gene expression profiling, protein expression profiling
- To collect a blood sample (optional) for DNA extraction and storage to provide data to investigate whether variability (if observed) in the PK, safety, efficacy or PD results could be explained by differences in the patient’s genotype

3. STUDY PLAN AND PROCEDURES

3.1 Overall study design

This Clinical Study Protocol has been subjected to a peer review according to AstraZeneca standard procedures.

This is a Phase I, open-label study to investigate the safety, tolerability and pharmacokinetics of twice-daily oral doses of AZD6244 when administered in combination with standard doses of selected chemotherapies.

Approximately 140 patients with advanced solid tumors will be recruited from 4 sites in the United States. Following written informed consent, patients will be screened for eligibility to receive study treatment.

The investigator is responsible for ensuring that initiation of AZD6244 treatment in combination with chemotherapy is done in strict accordance with local prescribing information for selected chemotherapies.

3.1.1 Part A dose escalation

Patients with confirmed eligibility will be enrolled to one of 4 treatment arms: (1) docetaxel, (2) dacarbazine, (3) erlotinib or (4) temsirolimus according to the standard clinical management of their disease. In both Parts A and B, patients with diseases other than those indicated in the drug label may be treated with AZD6244 Hyd-Sulfate in order to study the potential treatment combination in an expanded range of cancer diagnoses. AstraZeneca will reimburse study sites for study drugs used for patients where the drug given in combination does not represent the standard of care.

Sites are expected to enroll a patient in an open cohort within 2 weeks of cohort opening.

The starting dose of AZD6244 in each arm will be 50mg. Continuous dosing (commencing Cycle 1/Day 3 for docetaxel and dacarbazine and Cycle 1/Day 8 for erlotinib and temsirolimus) will be BD. The AZD6244 dose may be maintained or reduced as described in Section 3.2.1.

Table 2 Planned Dose Escalation Schedule for AZD6244

Dose Level	AZD6244 mg/day
1	50mg BD= 66% of monotherapy MTD
2	75mg BD (or 25 mg BD if reduction necessary)

Patients will be considered evaluable following at least 28 days of therapy from Cycle 1, Day 1, have experienced DLT, or at the discretion of the SRC. In addition, patients must have received approximately 80% of the planned doses of AZD6244 or at the discretion of the SRC. All dose escalation decisions will therefore be based on evaluable patients as determined by the SRC.

Patients withdrawing from treatment for reasons other than treatment-related toxicity before completing at least 28 days of therapy from Cycle 1 Day 1 or patients who complete cycle 1 but do not receive approximately 80% of the planned doses of AZD6244 will be replaced.

If DLT is observed in 1 of the first 3 patients, in a given cohort, at least 3 additional patients will be enrolled to that dose level, to treat a minimum of 6 patients.

AZD6244 + dose of standard chemotherapy will proceed according to the following principles:

A minimum of 3 evaluable patients will be enrolled for each cohort level.

· If 0/3 evaluable patients experience a DLT within 28 days of therapy from Cycle 1/Day 1, enroll patients at the next highest dose level

· If 1/3 evaluable patients experience a DLT within 28 days of therapy from Cycle 1/Day1, additional patients will be recruited at this dose level to treat a maximum of 6 evaluable patients before further escalation is considered

Then if;

· 1/6 evaluable patients experience a DLT, then dose will be escalated to the highest tolerated dose of the combination (AZD6244+chemotherapy)

· At any stage if ≥2 evaluable patients experience a DLT, recruitment to the cohort will cease and that dose will be defined as a non-tolerated dose. For more detailed information, see Figure 1 for the dose escalation decision flow chart

· The highest tolerated dose for each combination will not be defined until 6 evaluable patients have been evaluated at that dose level

All study arms will enroll in parallel and progress independently.

Dose escalation/dose reduction decisions will be made jointly, between investigators and AstraZeneca, meeting as a SRC prior to the opening of each cohort. Decisions will follow medical review of available clinical and laboratory data.

There will be no intra-patient dose escalation of AZD6244 this study. If patient experiences an AZD6244 related toxicity, their individual dose may be reduced or withheld at the investigators discretion in consultation with the AstraZeneca study physician. All cohort dose escalation/reductions will occur in consultation with the SRC. Once a patient has received an AZD6244 dose reduction, there is no provision for re-escalation or re-challenge with a higher dose in that patient.

There is currently no plan for dose escalation beyond the exposures observed at the monotherapy MTD. Once the highest tolerated dose of AZD6244 in combination with each regimen has been defined, Part B will commence.

3.1.2 Part B dose expansion

Dose expansion will commence when the highest tolerated dose for AZD6244 in combination with each chemotherapy has been determined. This is defined as the highest dose of the AZD6244 + chemotherapy combination with six evaluable patients and ≤1 DLT within 28 days of therapy from Cycle 1/Day 1.

All arms will recruit an additional 12 patients that are evaluable to give a minimum of 18 patients at the AZD6244 MTD in combination therapy as defined in Part A to further evaluate the safety, tolerability and pharmacokinetics of the dose. If in the expansion phase, the dose

identified in the dose-escalation phase is subsequently found to be non-tolerated, a lower dose may be explored. This will be determined by the SRC.

Part B Dose Exploration Plan for the Erlotinib arm

Rationale

The SRC determined AZD6244 50mg BID + erlotinib 100mg daily was not well tolerated during the Part B expansion. As a result, different dosing schedule for AZD6244 will be explored. AZD6244 will be administered once daily (50 mg QD - that is, 2 tablets of 25 mg of AZD6244 administrated in the morning) instead of 50 mg twice daily (BD). Based on pulsatile versus continuous dosing of targeted agents the inhibition of p-ERK levels appear to be critically influenced by Cmax, and p-ERK with levels in normal tissues return to normal in ~24 hours. Tumors do not recover as rapidly as normal tissues. With a short half-life (~6 hours) of AZD6244, this more pulsatile dosing may be more tolerable to normal tissues, while maximizing MEK inhibition overall with a higher peak dose. Below is the new dosing schedule and AZD6244 doses for each new cohort:

Cohort 3: AZD6244 50mg QD + erlotinib 100mg daily, 6 evaluable patients

- If 0 DLT in 6 evaluable patients occurs, escalate to 100mg QD (4 tablets of 25mg taken daily in the morning)

- If 1 DLT in 6 evaluable patients occurs, escalate to 75mg QD (3 tablets of 25 mg taken daily in the morning)

- If at least 2 DLTs in 6 evaluable patients occurs, no escalation will occur. The SRC and AstraZeneca Clinical Study Team will review all safety data to decide how to conclude this arm.

Cohort 4: AZD6244 will either be administered at 100mg QD or 75 mg QD, as determined from Cohort 3, + erlotinib 100mg daily, by 3-6 evaluable patients.

- If 0 DLTs / 3 patients or 1 DLT / 6 patients, dose escalate by no more than 50% increments as determined by SRC. Continue until MTD is declared.

- If 2 or more DLTs in 6 evaluable patients occur, de-escalate as per SRC decision. Once the MTD is established, expand to have minimum 12 and maximum 18 evaluable patients at the MTD.

Rationale for increasing AZD6244 dose from 50mg QD to 100mg QD (detailed above Cohort 3 to Cohort 4): PK Data from AZD6244 Study 5 indicates at a single dose of 75mg and a single dose of 100 mg, there is an overlap in drug exposure and target inhibition. The AUC is identical for both doses. Therefore, there may not be a significant difference between the two doses of AZD6244 for tolerability and efficacy.

The end of study is defined as 30 days after the last patient discontinues study treatment. However, final analysis will occur 6 months after the last patient recruited starts

investigational product.

Duration of treatment with AZD6244

Patients may continue to receive AZD6244 and the prescribed chemotherapy until progression or as long as they are do not experience dose-limiting toxicity, and are in the opinion of the investigator, continuing to derive benefit. Patients may also continue to receive AZD6244 alone after the standard chemotherapy has been completed if the investigator believes they are continuing to derive benefit from AZD6244 treatment. However, patients will not be allowed to begin new chemotherapy or other combinations of chemotherapy while continuing to receive AZD6244 therapy.

3.1.3 Definition of Dose Limiting Toxicity

Adverse events and other symptoms will be graded according to the NCI CTCAE (version http://ctep.cancer.gov/reporting/ctc.html).

Dose-limiting toxicity is defined as any of the following occurrences, when considered related to study treatments, during the first 28 days of therapy from Day 1/Cycle 1 despite best supportive care according to the standard of care at the study site:

Hematological Toxicities

- Afebrile Grade 4 neutropenia >5 days or Grade 4 neutropenia associated with fever (reading of body temperature >38.5C or 3 readings of body temperature >38.0C in a 24-hour period)
- Grade 4 thrombocytopenia
- Inability to resume AZD6244 dosing within 14 days of stopping due to treatment-related toxicity
- Inability to resume background chemotherapy within 14 days of a scheduled administration due to treatment-related toxicity (if the inability to resume chemotherapy is felt to be independent of AZD6244 therapy, the adverse event will not constitute a DLT)

Non-Hematological Toxicities:

- ≥Grade 3 non-hematological toxicities for >7 days. Grade 3 non-hematological toxicities that can be controlled to Grade 2 or less with appropriate treatment will not be considered dose limiting.

If a drug-related DLT should occur following at least 28 days of therapy from Cycle 1/Day 1, the course of action to be taken will be decided by the SRC.

For dose escalation purposes, only DLT occurring during the first 28 days after the start of therapy from Cycle 1/Day 1 will be considered. However, the incidence and type of DLTs that occur after 28 days of therapy from Cycle 1/Day 1 will be taken into account in determining dose escalation steps. SRC will make decisions regarding dose escalation steps.

Dose escalation will stop if the defined number of patients in any cohort experiences Dose Limiting Toxicity (DLT). See Section 3.1.3 for definition of DLT.

3.2 Study Procedures

Screening assessments to be performed can be found in the Study Plans for each treatment arm in Section 3.2.3. All protocol required screening assessments must be completed after obtaining written informed consent and prior to receiving first dose of study treatment. A complete ophthalmologic exam that includes visual acuity, visual fields, color vision, and slit lamp examination is required within 6 weeks of Day –8 to –3 or Cycle 1/ Day 1. Tumor assessments, high resolution thoracic CT scan (HRCT) in erlotinib and temsirolimus treatment arms only, and MUGA/echocardiogram for assessment of cardiac function will be conducted within 4 weeks of Day –8 to –3 or Cycle 1/ Day 1. Where available, archival tumor specimens should be obtained from all consented patients. (Further detail provided in laboratory manual).

Patients will also undergo a physical exam at screening and at the beginning of each chemotherapy cycle. Pre-menopausal female patients will have a urine or serum pregnancy test at screening and at the beginning of every new cycle, as long as they remain in the study.

For the purpose of the study plans in this protocol, a cycle is defined as 21 days. A 21 day cycle was selected for consistency across all arms of the protocol.

Patients will subsequently attend clinic on the following days:

- Docetaxel/dacarbazine arms: Days 1, 3, 8, and 15 of each 21-day cycle
- Erlotinib arm: Day –8 to –3 (cycle 1 only), 1, 7 (cycle 1 only), 8, and 15 of each 21-day cycle
- Temsirolimus arm: Day –8 to –3 (cycle 1 only), 1, 8, and 15 of each 21 day cycle

Minimum visit timing is every 4 weeks while on treatment. Patients free from DLT will be allowed repeat treatment cycles at the same dose of AZD6244 as their assigned cohort. Safety assessments will be performed as per study plan. Frequency of visits may change according to emerging safety profile.

Subsequent tumor assessments will be evaluated before Cycle 3 and every other cycle thereafter. In addition, patients whose disease progresses between scheduled visits should be confirmed by tumor assessments.

Twelve lead, resting ECGs will be performed in triplicate (one immediately after the other) during screening within 21 days of Cycle1/Day 1 for all treatment arms. Subsequent ECGs will be performed as follows:

- Cycle 1/Day 3- pre-dose, and 2 hours post dose of AZD6244 and cycle 2/Day 1 pre-infusion and 2 hours post dose of AZD6244 (docetaxel and dacarbazine arms)
- Cycle 1/Day –8 to –3-pre-dose, and 2 hours post-dose of AZD6244 and Cycle 1/Day 8-pre-dose and 2 hours post dose of AZD6244 (erlotinib and temsirolimus arms)
- Thereafter, ECGs will be performed as clinically indicated

A follow-up MUGA scan or echocardiogram will be performed at week 6, at 3 months and every 3 months while on study and as clinically warranted by the investigator.

All cardiorespiratory adverse events should be assessed with a chest X-ray, triplicate ECGs and O_2 saturation assessments. In the case of any events possibly with a cardiac etiology (e.g., congestive cardiac failure), a measure of cardiac ejection fraction (EF) and blood sample for BNP or NT-proBNP should be taken at the time of the event. Any events with a possible respiratory etiology (e.g., hypoxia demonstrated by a clinically significant drop in O_2 saturations) should be followed up by full PFTs and HRCT.

A complete ophthalmologic exam that includes visual acuity, visual fields, color vision, and slit lamp examination will be performed at screening (within 6 weeks of Day –8 to –3 or Cycle 1/Day 1) and at week 6 (± 2 weeks for patient convenience). In addition, if a patient receiving AZD6244 experiences an AE of visual disturbance a complete ophthalmologic examination must be performed. **If a patient receiving AZD6244 experiences blurred vision (CTCAE grade ≥2) concurrent with neurological symptoms (e.g. seizure, altered mental status, headaches) suggestive of possible Reversible Posterior Leucoencephalopathy syndrome (RPLS), a MRI scan of the brain should be performed, if this is not possible a CT scan can be used. Scans should be repeated upon resolution of neurological symptoms or upon clinically significant neurological deterioration.**

Patients may continue to receive AZD6244 and chemotherapy until disease progression, provided in the opinion of the investigator they are deriving clinical benefit, without unacceptable toxicity. Following discontinuation from AZD6244 for any reason, patients will have a Withdrawal from Treatment Visit, to include: PE, ECG, ophthalmologic exam, clinical chemistry/hematology, urinalysis, vital signs and AE assessments.

Following discontinuation from AZD6244 treatment for any reason, patients may receive any subsequent therapy for their disease at the discretion of the investigator. If new therapy received within 30 days of discontinuation of AZD6244 therapy, details of such treatment are to be recorded in the CRF.

3.2.1 Management of Toxicities for AZD6244

Treatment with AZD6244 should be withheld if one of the following toxicities is observed, despite supportive care:

- Any adverse events ≥CTCAE Grade 3 related to AZD6244
- DLT related to AZD6244

The management of any adverse event CTCAE Grade ≥3 or a DLT attributable to AZD6244 should be according to standard clinical practice for that event, e.g., diarrhea and rash should be treated with appropriate supportive care. Likewise, AEs attributable to standard therapies should be managed accordingly (See Section 3.2.2), such as administration of granulocyte-colony stimulating factor (G-CSF) as clinically indicated.

If AZD6244 related DLT or CTCAE ≥Grade 3 attributable to AZD6244 occurs within 28 days of therapy from Cycle 1/Day 1, the administration of AZD6244 will stop for this patient

If toxicity resolves to CTCAE Grade 1 or baseline, and the patient is able to resume therapy within 14 days, the patient may continue with the original AZD6244 dose. However, if therapy is withheld for >14 days, i.e. if recovery requires more than 14 days, and the patient continues in the study, the AZD6244 dose should be reduced, and the patient must wait until the start of a new (chemotherapy) cycle to resume AZD6244 therapy; i.e. Day 3 of next cycle.

If a patient experiences recurrence of the toxicity ≥CTCAE Grade 3, study medication should again be withheld until the toxicity improves to CTCAE Grade 1 or baseline. Upon recovery (if <14 days), treatment may resume at the previous dose level or at a permanently reduced dose. Note: If the dose was not reduced following the first occurrence of the toxicity, the dose must be reduced upon a second occurrence (i.e., treatment may only resume at the original dose after the first occurrence of the toxicity). Dose re-escalation is not permitted in this study.

If a CTCAE Grade ≥3 recurs after a second dose reduction, the patient must permanently discontinue study treatment.

No dose modification for CTCAE Grade 1 or 2 events unless clinically warranted by Investigator/SRC.

All dose reductions will be recorded in the appropriate CRF. Dose reductions or delays are not required for CTCAE Grade 1 or 2 toxicity.

In the event of a dose delay/reduction, with the exception of the collection of PK samples, patients should continue all other assessments as scheduled according to standard chemotherapy regimen.

3.2.1.1 Rash Management Guidance

The etiology of skin toxicities associated with the use of AZD6244 or anti-EGFR agents is uncertain and there are no established algorithms for rash management. In addition, it is not known whether the combination of erlotinib and AZD6244 in this protocol will increase the risk of rash. An algorithm based on best practices (Perez-Soler et al 2005) is offered as guidance to managing skin toxicities seen in patients being treated on this protocol.

The algorithm suggests a step-wise approach to rash management. If the rash is CTCAE (Grade 1), consider starting with topical steroids, clindamycin gel, or no treatment if the patient is asymptomatic. A high potency topical steroid, such as clobetasol propionate, may be considered early in patients with mild rash and may be used on the face. If the rash is CTCAE (Grade 2), consider adding an oral tetracycline and/or pimecrolimus cream. If the rash worsens beyond CTCAE Grade 3, dose reduction coupled with the addition of topical steroids is the recommended. Pruritis of any Grade may be treated with an antihistamine, such as diphenhydramine or hydroxyzine hydrochloride.

Secondary infection also appears to worsen rash. To reduce the likelihood of nasal infection, intranasal mupirocin may be considered. Infected rash may be treated with a short course of an oral tetracycline, such as minocycline hydrochloride. If there is a clinical diagnosis of impetigo, or an infection with S. aureus is confirmed, topical mupirocin might be used. Infected lesions suspected to be treatment resistant should be cultured. If there is no improvement after two weeks of treatment, therapy for the rash should be considered ineffective and discontinued.

All required treatment information and adverse event information for rash will be recorded on the appropriate case report form (CRF).

3.2.2 Dose Reductions of Standard Chemotherapy

If patients experience toxicity attributable to the standard chemotherapy, de-escalations/dose delays are permitted as per product label and will be recorded on the appropriate CRF. All dose reductions, changes, etc must be recorded in the appropriate CRF. The toxicity profiles of the chemotherapies docetaxel and dacarbazine and that of AZD6244 are not expected to be overlapping and therefore, patients experiencing toxicity attributable to docetaxel or dacarbazine will be allowed to continue AZD6244 therapy if chemotherapy is interrupted. If the investigator believes that AZD6244 has contributed to specific toxicities, AZD6244 treatment should be stopped or delayed. Unless DLT, AZD6244 treatment may be resumed after discussion between investigator, AZ medical science director and drug safety physician.

The temsirolimus dose may be reduced to 15 mg if the DLT(s) observed for the cohort is/are a known side effect of temsirolimus. Likewise, if the DLT(s) is/are a known side effect of AZD6244, than the AZD6244 dose will be reduced accordingly. All dosing decisions will be at the discretion of the SRC who will review the emerging tolerability and safety profile on an ongoing basis, and on completion of each cohort dose level, and decide whether to continue at 25 mg of temsirolimus or reduce to 15 mg for subsequent cohorts.

All patients may be pre-medicated according to the standard treatment regimens at each site. Medication, or other therapy to provide adequate supportive care, may be given as clinically necessary, including leuprolide acetate for prostate cancer, bisphosphonates for breast cancer, ESAs, etc. All such concomitant therapies starting or changing during the course of the study are allowed but should be recorded in the CRF as indicated in Section 3.5.5.

Patients experiencing DLT related to administration of standard chemotherapy will have dose reductions according to the specific, toxicity-directed management guidelines in the product label. All chemotherapy administration details will be recorded in the CRF. Details include start and stop date and time, dose reductions, modifications and delays. The following information/instruction is taken from the most current product labels.

Product labels for the standard chemotherapies used in this study are provided in the Appendices of the protocol (See Appendix F for docetaxel, Appendix G for dacarbazine, Appendix H for erlotinib and Appendix I for temsirolimus).

It is important that the individual product label be referred to for complete dosing instructions, lists of adverse reactions/toxicities and dose reduction/dose modifications needed because of these toxicities.

Docetaxel (DOC)

Hypersensitivity Reactions: Severe hypersensitivity reactions characterized by generalized rash/erythema, hypotension and/or bronchospasm, or very rarely fatal anaphylaxis, have been reported in patients pre-medicated with 3 days of corticosteroids. Hypersensitivity reactions require immediate discontinuation of the infusion. Patients with a history of severe hypersensitivity reactions should not be re-challenged.

Minor reactions such as flushing or localized skin reactions will not require interruption of therapy. More severe reactions, however, require the immediate discontinuation of infusion and aggressive therapy.

Cutaneous: Localized erythema of the extremities with edema followed by desquamation has been observed. In case of severe skin toxicity, an adjustment in dosage is recommended.

Fluid Retention: Severe fluid retention has been reported; patients developing peripheral edema may be treated with standard measures, e.g., salt restriction, oral diuretic(s).

Neurologic: Severe neurosensory symptoms (paresthesia, dysesthesia, pain) were observed in 5.5% (53/965) of metastatic breast cancer patients, and resulted in treatment discontinuation in 6.1%. When these symptoms occur, dosage must be adjusted. If symptoms persist, treatment should be discontinued.

Liver dysfunction: In case of AST/ALT >2.5 to <5 x ULN and AP <2.5 x ULN, or AST/ALT >1.5 to <5 x ULN and AP >2.5 to <5 x ULN, docetaxel should be reduced by 20%. In case of AST/ALT >5 x ULN and/or AP >5 x ULN therapy should be stopped.

If the patient experiences toxicity, the dosage should either be decreased according to label schedule or standard clinic practice, or the treatment should be discontinued. Patients who develop >Grade 3 peripheral neuropathy should have treatment discontinued entirely.

Dacarbazine

Hemopoietic depression is the most common toxicity with dacarbazine and involves primarily the leukocytes and platelets, although, anemia may sometimes occur. Leukopenia and thrombocytopenia may be severe enough to cause death. The possible bone marrow depression requires careful monitoring of white blood cells, red blood cells, and platelet levels. Hemopioetic toxicity may warrant temporary suspension or cessation of therapy with dacarbazine. Hepatic toxicity accompanied by hepatic vein thrombosis and hepatocellular necrosis resulting in death, has been reported. The incidence of such reactions has been low; approximately 0.01% of patients treated. This toxicity has been observed mostly when dacarbazine has been administered concomitantly with other anti-neoplastic drugs; however, it has also been reported in some patients treated with dacarbazine alone. Anaphylaxis can occur following the administration of dacarbazine.

Symptoms of anorexia, nausea, and vomiting are the most frequently noted of all toxic reactions. Over 90% of patients are affected with the initial few doses. The vomiting lasts 1-12 hours and is incompletely and unpredictably palliated with phenobarbital and/or prochlorperazine.

Patients have also experienced alopecia, facial flushing, facial paresthesia, an influenza-like syndrome of fever to 39°C, myalgias, malaise, erythematous and urticarial rashes and photosensitivity. There have also been a few reports of significant liver or renal function test abnormalities.

If marked leukopenia (particularly granulocytopenia) or thrombocytopenia occurs, dacarbazine should be discontinued until leukocyte and platelet counts return to satisfactory levels, usually within a week after the nadir.

Erlotinib

Per the updated product label issued September 2008, there have been cases of hepatic failure and hepatorenal syndrome (including fatalities) reported. There have also been cases of acute renal failure (including fatalities) and renal insufficiency.

Treatment with erlotinib should be used with extra caution in patients with total bilirubin > 3X ULN. Patients with hepatic impairment (total bilirubin > ULN or Child-Pugh A, B and C) should be clearly monitored during therapy with erlotinib. Dosing should be interrupted or discontinued if changes in liver function are severe such as doubling of total bilirubin and/or tripling of transaminases in the setting of pre-treatment values outside of normal range (please refer to current/recently updated erlotinib label for more details on this and on the Warnings section regarding 'hepatorenal syndrome'.

There have been infrequent reports of serious Interstitial Lung Disease (ILD)-like events, including fatalities, in patients receiving erlotinib for treatment of NSCLC, pancreatic cancer or other advanced solid tumors.

Reported diagnoses in patients suspected of having ILD-like events included pneumonitis, radiation pneumonitis, hypersensitivity pneumonitis, interstitial pneumonia, interstitial lung disease, obliterative bronchiolitis, pulmonary fibrosis, Acute Respiratory Distress Syndrome and lung infiltration. Symptoms started from 5 days to more than 9 months (median 39 days) after initiating erlotinib therapy. In the lung cancer trials most of the cases were associated with confounding or contributing factors such as concomitant/prior chemotherapy, prior radiotherapy, pre-existing parenchymal lung disease, metastatic lung disease, or pulmonary infections.

In the event of acute onset of new or progressive, unexplained pulmonary symptoms such as dyspnea, cough, and fever, erlotinib therapy should be interrupted pending diagnostic evaluation. If ILD is diagnosed, erlotinib should be discontinued and appropriate treatment instituted as necessary.

In the pancreatic cancer trial, reports of MI/ischemis, CVA, and microangiopathic haemolytic anemia with thrombocytopenia have been reported

Women of childbearing potential should be advised to avoid pregnancy while on erlotinib. Adequate contraceptive methods should be used during therapy, and for at least 2 weeks after completing therapy. Treatment should only be continued in pregnant women if the potential benefit to the mother outweighs the risk to the fetus. If erlotinib is used during pregnancy, the patient should be apprised of the potential hazard to the fetus or potential risk for loss of the pregnancy.

The most common adverse reactions in non-small cell lung cancer patients receiving single-agent erlotinib 150 mg were rash and diarrhea. Grade 3/4 rash and diarrhea occurred in 9% and 6%, respectively, in erlotinib treated patients. Rash and diarrhea each resulted in study discontinuation in 1% of ertolinib-treated patients. Six percent and 1% of patients needed dose reduction for rash and diarrhea, respectively. The median time to onset of rash was 8 days, and the median time to onset of diarrhea was 12 days.

The most common adverse reactions in pancreatic cancer patients receiving erlotinib 100 mg plus gemcitabine were fatigue, rash, nausea, anorexia and diarrhea. In the erlotinib plus gemcitabine arm, Grade 3/4 rash and diarrhea were each reported in 5% of erlotinib plus gemcitabine-treated patients.

NCI-CTC Grade 3 conjunctivitis and keratitis have been reported infrequently in patients receiving erlotinib therapy in the NSCLC and pancreatic cancer clinical trials. Corneal ulcerations may also occur

Dose Modifications in patients who develop an acute onset of new or progressive pulmonary symptoms, such as dyspnea, cough or fever, treatment with erlotinib should be interrupted

pending diagnostic evaluation. If ILD is diagnosed, erlotinib should be discontinued and appropriate treatment instituted as necessary.

Diarrhea can usually be managed with loperamide. Patients with severe diarrhea who are unresponsive to loperamide or who become dehydrated may require dose reduction or temporary interruption of therapy. Patients with severe skin reactions may also require dose reduction or temporary interruption of therapy.

When dose reduction is necessary, the erlotinib dose should be reduced at the discretion of the SRC.

Temsirolimus

- Hypersensitivity reactions manifested by symptoms, including, but not limited to anaphylaxis, dyspnea, flushing, and chest pain have been observed with temsirolimus.
- Serum glucose, serum cholesterol, and triglycerides should be tested before and during treatment with temsirolimus.
- The use of temsirolimus may result in immunosuppression.
- Cases of interstitial lung disease, some resulting in death, have occurred.
- Cases of fatal bowel perforation occurred with temsirolimus.
- Cases of rapidly progressive and sometimes fatal acute renal failure
- Abnormal wound healing
- Patients with central nervous system tumors (primary CNS tumor or metastases) and/or receiving anticoagulation therapy may be at an increased risk of developing intracerebral bleeding (including fatal outcomes) while receiving temsirolimus.
- Live vaccinations and close contact with those who received live vaccines should be avoided.
- Patients and their partners should be advised to avoid pregnancy throughout treatment and for 3 months after temsirolimus therapy has stopped.
- The most common (incidence ≥30%) adverse reactions observed with temsirolimus are: rash, asthenia, mucositis, nausea, edema, and anorexia.
- Most common grades 3/4 adverse events included asthenia, dyspnea, hemoglobin decreased, lymphocytes decreased, glucose increased, phosphorus decreased, and triglycerides increased.

- Women of childbearing potential should be advised to avoid becoming pregnant throughout treatment and for 3 months after temsirolimus therapy has stopped.

- Men should be counseled regarding the effects of temsirolimus on the fetus and sperm prior to starting treatment. Men with partners of childbearing potential should use reliable contraception throughout treatment and are recommended to continue this for 3 months after the last dose of temsirolimus.

- When temsirolimus dose reduction is needed, dose will be reduced at the discretion of the SRC.

Figure 1 Study flow chart

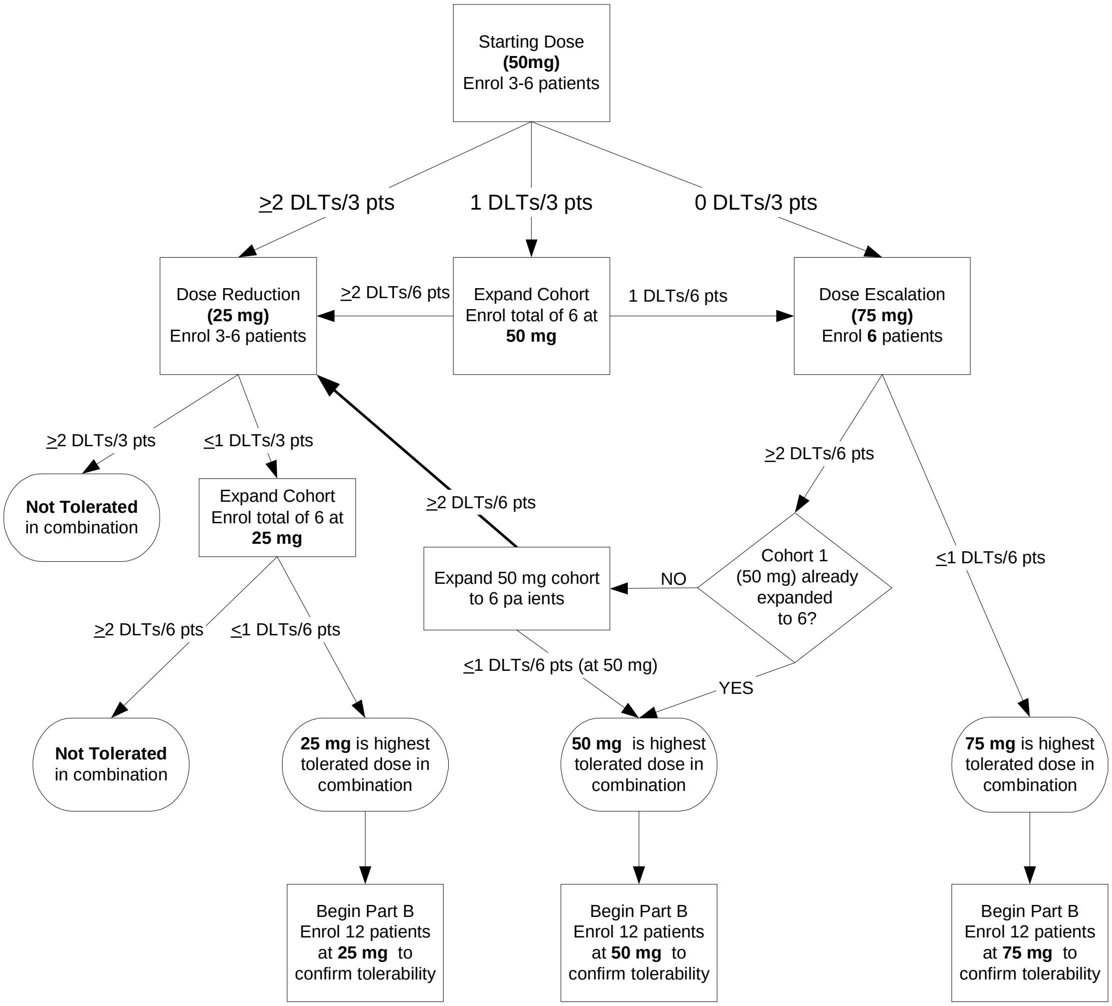

3.2.3 Study plans and PK tables

Docetaxel will be given at 75 mg/m^2 IV infusion over 60 min Day 1 q 21

Table 3 Study plan: Docetaxel

**Visit**	**1**	**2**	**3**	**4**	**5**	**6**	**7**	**8**	**9**	**10**	**11, 12**	**WD[m]**	**30 FU**
**Cycle**				**1**		**2**		**2**		**3**	**4, 5**		
**Day**	**Scr**	**1**	**3**	**8**	**15**	**1**	**3**	**8**	**15**	**1**	**1**		
Informed consent	X[a]												
Archived tumor [r]	X												
Demography	X												
Medical history/Surgical history/smoking status	X[a]												
Extent of disease	X[a]												
Inclusion/exclusion criteria	X[a]	X											
Physical examination[c]	X[a]					X				X	X	X	
Vital signs[d]	X[a]	X	X	X	X	X	X	X	X	X	X	X	
Height	X[a]												
Weight	X[a]	X		X	X	X		X	X	X	X	X	
Electrocardiogram[e]	X[a]		X			X					X[o]	X	
Hematology[f]	X[a]	X		X	X	X		X	X	X	X	X	X
Clinical chemistry[g]	X[a]	X		X	X	X		X	X	X	X	X	X
Urinalysis[h]	X[a]	X	X			X				X	X	X	
Pregnancy test[i]	X					X				X	X		
WHO Performance Status	X[a]					X				X	X	X	
Disease evaluation[n]	X									X	X	X	
Previous cancer tx	X												
MUGA/Echo [p]	X									X	X		
O_2 saturation measurement[u]	X						As required						
BNP & Troponin[t]	X[a]					X				X	X		
Chest X-ray	X[a]						As required						
Ophthalmologic Exam [q]	X									X		X	
AZD6244 daily BD dosing			X					Daily BD dosing					
Docetaxel infusion		X				X				X	X		

Table 3 Study plan: Docetaxel

**Visit**	**1**	**2**	**3**	**4**	**5**	**6**	**7**	**8**	**9**	**10**	**11, 12**	**WD[m]**	**30 FU**
**Cycle**				**1**		**2**		**2**		**3**	**4, 5**		
**Day**	**Scr**	**1**	**3**	**8**	**15**	**1**	**3**	**8**	**15**	**1**	**1**		
PK blood sampling docetaxel[j]		X	X			X							
PK blood sampling for AZD6244[k]			X			X							
Optional blood sample for genetic research[b]		X											
Adverse event review	X	X	X	X	X	X	X	X	X	X	X	X	X
Concomitant medication	X[a]	X	X	X	X	X	X	X	X	X	X	X	
Post WD cancer trt												X	

Assessments and treatment should be carried out as specified in the study plan. After the PK phase is completed. if the scheduled study day falls on a weekend or holiday, the treatment or assessment may be delayed or advanced by up to 3 days relative to Cycle 1/Day 1

a To be performed within 21 days before planned Cycle 1/Day 1

b Consenting patients only (separate consent required for genetic analysis).

c Physical exam and BSA calculation will be performed at the beginning of each cycle.

d Vital signs include blood pressure (BP), Pulse rate, and temperature. BP and pulse rate should be taken after patient has been at rest in supine position for 5 minutes and before any non-PK blood sampling.

e 12-lead ECG to be performed Cycle 1/ Day 3 pre-dose and 2 hours post-dose of AZD6244, Cycle 2/Day 1 pre-infusion and 2 hours post dose.

f Hematology to be performed weekly during Cycle 1 and Cycle 2 and once per cycle for subsequent cycles. Hematology samples need only be collected on Cycle 1/Day 1 if the screening assessments were taken more than 2 days before.

g Chemistry to be performed weekly during Cycle 1 and Cycle 2 and once per cycle for subsequent cycles. Chemistry samples need only be collected on Cycle 1/Day 1 if the screening assessments were taken more than 2 days before.

h Urinalysis to include pH, blood, protein, glucose, ketones, bilirubin. For patients who develop proteinuria >2+ (or experience worsening of pre-existing condition), a repeat urinalysis should be performed. If this second test confirms the laboratory abnormality the full 24-hour urine collection should be performed

I Females of child bearing potential must have a negative urine or serum pregnancy test within 72h before planned Cycle 1/Day 1 and at the start of each new cycle.

j Blood samples for docetaxel PK will be collected on Cycle 1/Day 1 and Cycle 2/Day 1. See Table 4 for complete schedule.

k Blood samples for AZD6244 PK to be collected on Cycle 1/Day 3 and Cycle 2/Day 1. See Table 4 for complete schedule.

l Patients receiving benefit from AZD6244 in combination with anti-cancer therapy can continue provided they are receiving benefit and are free from intolerable toxicity. Safety visits will be performed every 3 weeks when patients return for their next cycle of anti-cancer therapy until discontinuation. Tumor assessment will be performed at 6-week intervals.

m Adverse events will be followed up for 30 days following last dose of AZD6244

n Baseline tumor (RECIST)assessment can be performed up to 4 weeks before planned first dosing with AZD6244; Subsequent tumor assessments will be conducted prior to the 3rd cycle and every other cycle thereafter. Tumor assessments should also be performed at the withdrawal visit unless performed within the last 4 weeks. All tumor assessments may be performed ± 1 week relative to the specified visit date.

[o] ECG to be performed only as clinically indicated.

[p] MUGA/echocardiogram to be performed at screening (within 4 weeks before planned Cycle 1/Day 1), at week 6, 3 months, and every 3 months while on study (and as clinically warranted)

[q] Ophthalmologic exam must be performed within 6 weeks prior to planned Cycle 1/Day 1, at week 6 (+/- 2 weeks for convenience), as required and at withdrawal.

[r] Archival tumor sample (in consenting patients separate consent required for all biomarker research).

t Unscheduled samples should be taken as clinically warranted

u If there is a clinically significant drop in O_2 saturation, this event should be followed up with full pulmonary function tests and HRCT scan of the chest.

Table 4 PK sample collection docetaxel and AZD6244

Study Day	Time	Docetaxel	Time	AZD6244
C1/ D1	Pre-infusion	X		
	30 min[a]	X		
End of infusion	End/1 h[a]	X		
	1 h and 30 min[a]	X		
	2 h[a]	X		
	4 h[a]	X		
	8 h[a]	X		
	12 h[a]	X		
C1/ D2	24 h[a]	X		
	36 h[a]	X		
C1/ D3	48 h[a]	X	Pre-dose	X
			30 min	X[b]
			1 h	X[b]
			1 h and 30 min	X[b]
			2 h	X[b]
			4 h	X[b]
			8 h	X[b]
			12 h	X[b]
C2/ D1	Pre-infusion	X	Pre-dose (within 10 minutes prior to dosing)	X
	30 min[a]	X	30 min	X[b]
End of infusion	End/1 h[a]	X	1 h	X[b]
	1 h and 30 min[a]	X	1 h and 30 min	X[b]
	2 h[a]	X	2 h	X[b]
	4 h[a]	X	4 h	X[b]
	8 h[a]	X	8 h	X[b]
	12 h[a]	X	12 h	X[b]

a Relative to start of infusion
b Relative to time of dose of AZD6244
*PK does not need to be collected for Part B dose expansion

Dacarbazine will be given at 1000 mg/m^2 iv infusion over 60 minutes Day 1 q21

Table 5 Study plan: Dacarbazine

**Visit**	**1**	**2**	**3**	**4**	**5**	**6**	**7**	**8**	**9**	**10**	**11, 12**	**WD[m]**	**30 FU**
**Cycle**		**1**				**2**		**2**		**3**	**4, 5**		
**Day**	**Scr**	**1**	**3**	**8**	**15**	**1**	**3**	**8**	**15**	**1**	**1**		
Informed consent	X[a]												
Archived tumor [r]	X												
Demography	X												
Medical history/Surgical history/smoking status	X[a]												
Extent of disease	X[a]												
Inclusion/exclusion criteria	X[a]	X											
Physical examination[c]	X[a]					X				X	X	X	
Vital signs[d]	X[a]	X	X	X	X	X	X	X	X	X	X	X	
Height	X[a]												
Weight	X[a]	X		X	X	X		X		X	X	X	X
Electrocardiogram[e]	X[a]		X			X					X[o]	X	
Hematology[f]	X[a]	X		X	X	X		X	X	X	X	X	X
Clinical chemistry[g]	X[a]	X		X	X	X		X	X	X	X	X	X
Urinalysis[h]	X[a]	X	X			X				X	X	X	
Pregnancy test[i]	X					X				X	X		
WHO Performance Status	X[a]					X				X	X	X	
Disease evaluation[n]	X									X	X	X	
Previous cancer tx	X												
MUGA/Echo [p]	X									X	X		
O_2 saturation measurement[u]	X						As required						
BNP & Troponin[t]	X[a]					X				X	X		
Chest X-ray	X[a]						As required						
Ophthalmologic Exam [q]	X									X		X	
AZD6244 daily BD dosing			X					Daily BD dosing					
Dacarbazine infusion		X				X				X	X		
PK blood sampling dacarbazine[j]		X	X			X							

Table 5 Study plan: Dacarbazine

**Visit**	**1**	**2**	**3**	**4**	**5**	**6**	**7**	**8**	**9**	**10**	**11, 12**	**WD**[m]	**30 FU**
**Cycle**				**1**				**2**			**4, 5**		
						**2**				**3**			
**Day**	**Scr**	**1**	**3**	**8**	**15**	**1**	**3**	**8**	**15**	**1**	**1**		
PK blood sampling for AZD6244[k]			X			X							
Optional blood sample for genetic research[b]		X											
Adverse event review	X	X	X	X	X	X	X	X	X	X	X	X	X
Concomitant medication	X[a]	X	X	X	X	X	X	X	X	X	X	X	
Post WD cancer trt												X	

Assessments and treatment should be carried out as specified in the study plan. After the PK phase has been completed, if the scheduled study day falls on a weekend or holiday, the treatment or assessment may be delayed or advanced by up to 3 days relative to Cycle 1/Day 1

a To be performed within 21 days before planned Cycle 1/Day 1

b Consenting patients only (separate consent required for genetic analysis).

c Physical exam and BSA calculation will be performed at the beginning of each cycle.

d Vital signs include blood pressure (BP), Pulse rate, and temperature. BP and pulse rate should be taken after patient has been at rest in supine position for 5 minutes and before any non-PK blood sampling.

e 12-lead ECG to be performed Cycle 1/ Day 3 pre-dose and 2 hours post-dose of AZD6244, Cycle 2/Day 1 pre-infusion and 2 hours post dose.

f Hematology to be performed weekly during Cycle 1 and Cycle 2 and once per cycle for subsequent cycles. Hematology samples need only be collected on Cycle 1/Day 1 if the screening assessments were taken more than 2 days before.

g Chemistry to be performed weekly during Cycle 1 and Cycle 2 and once per cycle for subsequent cycles. Chemistry samples need only be collected on Cycle 1/Day 1 if the screening assessments were taken more than 2 days before.

h Urinalysis to include pH, blood, protein, glucose, ketones, bilirubin. For patients who develop proteinuria >2+ (or experience worsening of pre-existing condition), a repeat urinalysis should be performed. If this second test confirms the laboratory abnormality the full 24-hour urine collection should be performed

I Females of child bearing potential must have a negative urine or serum pregnancy test within 72h before planned Cycle 1/Day 1 and at the start of each new cycle.

j Blood samples for dacarbazine PK will be collected on Cycle 1/Day 1 and Cycle 2/Day 1. See Table 6 for complete schedule.

k Blood samples for AZD6244 PK to be collected on Cycle 1/Day 3 and Cycle 2/Day 1. See Table 6 for complete schedule.

l Patients receiving benefit from AZD6244 in combination with anti-cancer therapy can continue provided they are receiving benefit and are free from intolerable toxicity. Safety visits will be performed every 3 weeks when patients return for their next cycle of anti-cancer therapy until discontinuation. Tumor assessment will be performed at 6-week intervals.

m Adverse events will be followed up for 30 days following last dose of AZD6244

n Baseline tumor (RECIST)assessment can be performed up to 4 weeks before planned first dosing with AZD6244; Subsequent tumor assessments will be conducted prior to the 3rd cycle and every other cycle thereafter. Tumor assessments should also be performed at the withdrawal visit unless performed within the last 4 weeks. All tumor assessments may be performed ± 1 week relative to the specified visit date.

o ECG to be performed only as clinically indicated.

[p] MUGA/echocardiogram to be performed at screening (within 4 weeks before planned Cycle 1/Day 1), at week 6, 3 months, and every 3 months while on study (and as clinically warranted)

[q] Ophthalmologic exam must be performed within 6 weeks prior to planned Cycle 1/Day 1, at week 6 (+/- 2 weeks for convenience), as required and at withdrawal.

[r] Archival tumor sample (in consenting patients separate consent required for all biomarker research).

[s] Optional pre-dose biomarker sample; separate biomarker consent required prior to taking sample

t Unscheduled samples should be taken as clinically warranted

u If there is a clinically significant drop in O_2 saturation, this event should be followed up with full pulmonary function tests and HRCT scan of the chest.

Table 6 PK sample collection dacarbazine and AZD6244

Study Day	Time	Dacarbazine	Time	AZD6244
C1/ D1	Pre-infusion	X		
End of infusion	End/1 h[a]	X		
	1 h and 30 min[a]	X		
	3 h[a]	X		
	6 h[a]	X		
	12 h[a]	X		
C1/ D3	48 h[a]	X	Pre-dose	X
			30 min	X[b]
			1 h	X[b]
			1 h and 30 min	X[b]
			2 h	X[b]
			4 h	X[b]
			8 h	X[b]
			12 h	X[b]
C2/ D1	Pre-infusion[a]	X	Pre-dose (within 10 minutes prior to dosing)	X
End of infusion	End/1 h[a]	X	30 min	X[b]
	1 h and 30 min[a]	X	1 h	X[b]
	3 h[a]	X	1 h and 30 min	X[b]
	6 h[a]	X	2 h	X[b]
	12 h[a]	X	4 h	X[b]
			8 h	X[b]
			12 h	X[b]

a Relative to start of infusion
b Relative to time of dose of AZD6244

Erlotinib is given 100 mg po daily starting Cycle 1/Day 1 q 21

Table 7 Study plan: Erlotinib

Visit	1		2	3	4	5	6	7	8	9	10, 11	WD[m]	30 FU
**Cycle**			1				2	2		3	4, 5		
**Day**	Scr	-8 to -3	1	7	8	15	1	8	15	1	1		
Informed consent	X[a]												
Archived tumor [r]	X												
Demography	X												
Medical history/Surgical history/smoking status	X[a]												
Extent of disease	X[a]												
Inclusion/exclusion criteria	X[a]	X											
Physical examination[c]	X[a]						X			X	X	X	
Vital signs[d]	X[a]	X	X	X	X	X	X	X	X	X	X	X	
Height	X[a]												
Weight	X[a]		X		X	X	X	X	X	X	X	X	X
Electrocardiogram[e]	X[a]	X			X						X[o]	X	
Hematology[f]	X[a]		X		X	X	X	X	X	X	X	X	X
Clinical chemistry[g]	X[a]		X		X	X	X	X	X	X	X	X	X
Urinalysis[h]	X[a]		X				X			X	X	X	
Pregnancy test[i]	X	X					X			X	X		
WHO Performance Status	X[a]						X			X	X	X	
Disease evaluation[n]	X									X	X	X	
Previous cancer tx	X												
MUGA/Echo[p]	X									X	X		
O_2 saturation measurement[w]	X							As required					
BNP & Troponin[v]	X[a]						X			X	X		
Chest X-ray	X[a]							As required					
Ophthalmologic Exam[q]	X									X		X	
High Resolution thoracic (CT) scan[t]	X								As Required				
Single dose of AZD6244[u]		X											

Table 7 Study plan: Erlotinib

**Visit**	1		2	3	4	5	6	7	8	9	10, 11	WD[m]	30 FU
**Cycle**			1				2	2		3	4, 5		
**Day**	Scr	-8 to -3	1	7	8	15	1	8	15	1	1		
AZD6244 daily BD dosing					X			Daily BD dosing					
Erlotinib Dosing			X				Daily erlotinib dosing						
PK blood sampling erlotinib[j]				X	X								
PK blood sampling for AZD6244[k]		X			X								
Optional blood sample for genetic research[b]		X											
Optional plasma sample for biomarker analysis[s]		X											
Adverse event review	X	X	X	X	X	X	X	X	X	X	X	X	X
Concomitant medication	X	X	X	X	X	X	X	X	X	X	X	X	
Post WD cancer trt												X	

Assessments and treatment should be carried out as specified in the study plan. See Table 8 for PK sample flexibility. After the PK phase has been completed, if the scheduled study day falls on a weekend or holiday, the treatment or assessment may be delayed or advanced by up to 3 days relative to Cycle 1/Day

a To be performed within 21 days before planned Cycle 1/Day 1

b Consenting patients only (separate consent required for genetic analysis).

c Physical exam will be performed at the beginning of each cycle.

d Vital signs include blood pressure (BP), Pulse rate, and temperature. BP and pulse rate should be taken after patient has been at rest in supine position for 5 minutes and before any non-PK blood sampling.

e 12-lead ECG to be performed Cycle 1/ Day-8 to day -3 pre-dose and 2 hours post-dose of AZD6244 and Cycle 1/Day 8 pre-dose and 2 hours post dose.

f Hematology to be performed weekly during Cycle 1 and Cycle 2 and once per cycle for subsequent cycles.

g Chemistry to be performed weekly during Cycle 1 and Cycle 2 and once per cycle for subsequent cycles.

h Urinalysis to include pH, blood, protein, glucose, ketones, bilirubin. For patients who develop proteinuria >2+ (or experience worsening of pre-existing condition), a repeat urinalysis should be performed. If this second test confirms the laboratory abnormality the full 24-hour urine collection should be performed.

I Females of child bearing potential must have a negative urine or serum pregnancy test within 72h before first dose of AZD6244 (Day –8 to –3) and at the start of each new cycle.

j Blood samples for erlotinib PK will be collected on Cycle 1/Day 7 and Cycle 1/Day 8. See Table 8 for complete schedule.

k Blood samples for AZD6244 PK to be collected three to eight days prior to Cycle 1/Day 1 and Cycle 1/Day 8. See Table 8 for complete schedule.

l Patients receiving benefit from AZD6244 in combination with anti-cancer therapy can continue provided they are receiving benefit and are free from intolerable toxicity. Safety visits will be performed every 3 weeks when patients return for their next cycle of anti-cancer therapy until discontinuation. Tumor assessment will be performed at 6-week intervals.

m Adverse events will be followed up for 30 days following last dose of AZD6244

[n] Baseline tumor (RECIST)assessment can be performed up to 4 weeks before planned first dosing with AZD6244; Subsequent tumor assessments will be conducted prior to the 3rd cycle and every other cycle thereafter. Tumor assessments should also be performed at the withdrawal visit unless performed within the last 4 weeks. All tumor assessments may be performed ± 1 week relative to the specified visit date.

[o] ECG to be performed only as clinically indicated.

[p] MUGA/echocardiogram to be performed at screening (within 4 weeks before planned first dose of AZD6244), at week 6, 3 months, and every 3 months while on study (and as clinically warranted)

[q] Ophthalmologic exam must be performed within 6 weeks prior to planned first dose of AZD6244, at week 6 (+/- 2 weeks for convenience), as required and at withdrawal.

[r] Archival tumor sample (in consenting patients separate consent required for all biomarker research).

[s] Optional pre-dose biomarker sample; separate biomarker consent required prior to taking sample

[t] High resolution Thoracic CT scan should be performed as screening (within 4 weeks before planned first dose of AZD6244) and as clinically indicated or if any adverse event with a possible respiratory etiology occurs

u Single dose of AZD6244 Hyd-Sulfate will be given three to eight days prior to Cycle1/Day 1. The dose will be what the patient is scheduled to receive BD.

v Unscheduled samples should be taken as clinically warranted

w If there is a clinically significant drop in O_2 saturation, this event should be followed up with full pulmonary function tests and HRCT scan of the chest.

Table 8 PK sample collection Erlotinib and AZD6244

Study Day	Time	Erlotinib	Time	AZD6244
C1/D –3 (flexible to be Day –3 to -8 but must not be later than Day –3)			Pre-dose	X
(AZD6244 alone)			30 min	X[b]
			1 h	X[b]
			1 h and 30 min	X[b]
			2 h	X[b]
			4 h	X[b]
			8 h	X[b]
			12 h	X[b]
			24 h	X[b]
C1/D1			72-192 h[d]	X[b]
C1/D7 (flexible to be C1/D7 or later but must not be before C1/D7)	Pre-dose[c]	X		
(Erlotinib alone)	30 min[a]	X		
	1 h[a]	X		
	2 h[a]	X		
	3 h[a]	X		
	4 h[a]	X		
	6 h[a]	X		
	8 h[a]	X		
	12 h[a]	X		
C1/D8-(must be whatever day the first AZD6244 + erlotinib combo dose is given)	Pre-dose[c]	X	Pre-dose[c]	X
(AZD6244 + erlotinib)	30 min[a]	X	30 min	X[b]
	1 h[a]	X	1 h	X[b]
	2 h[a]	X	1 h and 30 min	X[b]
	3 h[a]	X	2 h	X[b]

Table 8 PK sample collection Erlotinib and AZD6244

Study Day	Time	Erlotinib	Time	AZD6244
	4 h[a]	X	4 h	X[b]
	6 h[a]	X	8 h	X[b]
	8 h[a]	X	12 h	X[b]
	12 h[a]	X		

a Relative to time of dose of erlotinib
b Relative to time of dose of AZD6244
c Within 10 minutes prior to dosing
d This sample should be taken within 10 minutes prior to dosing with chemotherapy (assuming AZD6244 was administered on Day –3, this should correspond to 72h post AZD6244 dose)

Temsirolimus is given 25 mg IV over 60 minutes on days 1, 8, and 15 of each 21 day

Table 9 Study plan: Temsirolimus

**Visit**	1		2	3	4	5	6	7	8	9, 10	WD[m]	30 FU
**Cycle**			1			2			3	4, 5		
**Day**	Scr	-8 to -3	1	8	15	1	8	15	1	1		
Informed consent	X[a]											
Archived tumor [r]	X											
Demography	X											
Medical history/Surgical history/smoking status	X[a]											
Extent of disease	X[a]											
Inclusion/exclusion criteria	X[a]	X										
Physical examination[c]	X[a]					X			X	X	X	
Vital signs[d]	X[a]	X	X	X	X	X	X	X	X	X	X	
Height	X[a]											
Weight	X[a]		X	X	X	X	X	X	X	X	X	X
Electrocardiogram[e]	X[a]	X		X						X[o]	X	
Hematology[f]	X[a]		X	X	X	X	X	X	X	X	X	X
Clinical chemistry[g]	X[a]		X	X	X	X	X	X	X	X	X	X
Urinalysis[h]	X[a]		X			X			X	X	X	
Pregnancy test[i]	X	X				X			X	X		
24 hour urine for total protein[x]	X											
WHO Performance Status	X[a]					X			X	X	X	
Disease evaluation[n]	X								X	X	X	
Previous cancer tx	X											
MUGA/Echo[p]	X								X	X		
O_2 saturation measurement[w]	X						As required					
BNP & Troponin[v]	X[a]					X			X	X		
Chest X-ray	X[a]						As required					
Ophthalmologic Exam[q]	X								X		X	
High Resolution thoracic (CT) scan[t]	X							As Required				

Table 9 Study plan: Temsirolimus

**Visit**		1	2	3	4	5	6	7	8	9, 10	WD[m]	30 FU
**Cycle**			1			2			3	4, 5		
**Day**	Scr	-8 to -3	1	8	15	1	8	15	1	1		
Single dose of AZD6244[u]		X										
AZD6244 daily BD dosing				X			Daily BD Dosing					
Temsirolimus infusion			X	X	X	X	X	X	X	X		
PK blood sampling temsirolimus[j]			X	X								
PK blood sampling for AZD6244[k]		X		X								
Optional blood sample for genetic research[b]		X										
Optional plasma sample for biomarker analysis[s]		X										
Adverse event review	X	X	X	X	X	X	X	X	X	X	X	X
Concomitant medication	X	X	X	X	X	X	X	X	X	X	X	
Post WD cancer trt											X	

Assessments and treatment should be carried out as specified in the study plan. See Table 10 for PK sample flexibility. After the PK phase has been completes, if the scheduled study day falls on a weekend or holiday, the treatment or assessment may be delayed or advanced by up to 3 days relative to Cycle 1/Day 1

a To be performed within 21 days before planned Cycle 1/Day 1

b Consenting patients only (separate consent required for genetic analysis).

c Physical exam will be performed at the beginning of each cycle.

d Vital signs include blood pressure (BP), Pulse rate, and temperature. BP and pulse rate should be taken after patient has been at rest in supine position for 5 minutes and before any non-PK blood sampling.

e 12-lead ECG to be performed Cycle 1/ Day-8 to -3 pre-dose and 2 hours post-dose of AZD6244, Cycle 1/Day 8 pre-infusion and 2 hours post dose.

f Hematology to be performed weekly during Cycle 1 and Cycle 2 and once per cycle for subsequent cycles.

g Chemistry to be performed weekly during Cycle 1 and Cycle 2 and once per cycle for subsequent cycles. Screening will include fasting glucose, triglycerides, and cholesterol.

h Urinalysis to include pH, blood, protein, glucose, ketones, bilirubin. For patients who develop proteinuria >2+ (or experience worsening of pre-existing condition), a repeat urinalysis should be performed. If this second test confirms the laboratory abnormality the full 24-hour urine collection should be performed.

I Females of child bearing potential must have a negative urine or serum pregnancy test within 72h before first dose of AZD6244 (Day –8 to –3) and at the start of each new cycle.

j Blood samples for temsirolimus PK will be collected on Cycle 1/Day 1 and Cycle 1/Day 8. See Table 10 for complete schedule.

k Blood samples for AZD6244 PK to be collected three to eight days before Cycle 1/Day 1 and Cycle 1/Day 8. See Table 10 for complete schedule.

l Patients receiving benefit from AZD6244 in combination with anti-cancer therapy can continue provided they are receiving benefit and are free from intolerable toxicity. Safety visits will be performed every 3

weeks when patients return for their next cycle of anti-cancer therapy until discontinuation. Tumor assessment will be performed at 6-week intervals.

[m] Adverse events will be followed up for 30 days following last dose of AZD6244

[n] Baseline tumor (RECIST)assessment can be performed up to 4 weeks before planned first dosing with AZD6244; Subsequent tumor assessments will be conducted prior to the 3rd cycle and every other cycle thereafter. Tumor assessments should also be performed at the withdrawal visit unless performed within the last 4 weeks. All tumor assessments may be performed ± 1 week relative to the specified visit date.

[o] ECG to be performed only as clinically indicated.

[p] MUGA/echocardiogram to be performed at screening (within 4 weeks before planned first dose of AZD6244), at week 6, 3 months, and every 3 months while on study (and as clinically warranted)

[q] Ophthalmologic exam must be performed within 6 weeks prior to planned first dose of AZD6244, at week 6 (+/- 2 weeks for convenience), as required and at withdrawal.

[r] Archival tumor sample (in consenting patients separate consent required for all biomarker research).

[s] Optional pre-dose biomarker sample; separate biomarker consent required prior to taking sample

[t] High resolution Thoracic CT scan should be performed as screening (within 4 weeks before planned first dose of AZD6244) and as clinically indicated or if any adverse event with a possible respiratory etiology occurs

u Single dose of AZD6244 Hyd-Sulfate will be given three to eight days prior to Cycle1/Day 1. The dose will be what the patient is scheduled to receive BD.

v Unscheduled samples should be collected as clinically warranted

w If there is a clinically significant drop in O_2 saturation, this event should be followed up with full pulmonary function tests and HRCT scan of the chest.

x 24 hour urine for total protein at screening plus follow-up with regular dipsticks at the investigator’s discretion. Repeat 24 hour collection if clinically indicated.

Table 10 PK sample collection Temsirolimus and AZD6244

Study Day	Time	Temsirolimus	Time	AZD6244
C1/D –3 (flexible to be Day –3 to -8 but must not be later than Day -3)			Pre-dose	X
(AZD6244 alone)			30 min	X[b]
			1 h	X[b]
			1 h and 30 min	X[b]
			2 h	X[b]
			4 h	X[b]
			8 h	X[b]
			12 h	X[b]
			24 h	X[b]
C1/D1	Pre-infusion	X	72-192h[d]	X[b]
(Temsirolimus alone)	30 min[a]	X		
End of infusion	End/1 h[a]	X		
	1 h and 30 min[a]	X		
	2 h[a]	X		
	3 h[a]	X		
	4 h[a]	X		
	8 h[a]	X		
	12 h[a]	X		
C1/D2	24 h[a]	X		
C1/D5 (flexible for ± 24 hours)	96 h[a]	X		
C1/D8 (must be whatever day the first AZD6244 + temsirolimus combo dose is given)	Pre-infusion[c]	X	Pre-dose[c]	X
(AZD6244 + temsirolimus)	30 min[a]	X	30 min	X[b]

Table 10 PK sample collection Temsirolimus and AZD6244

Study Day	Time	Temsirolimus	Time	AZD6244
End of infusion	End/1 h[a]	X	1 h	X[b]
	1 h and 30 min[a]	X	1 h and 30 min	X[b]
	2 h[a]	X	2 h	X[b]
	3 h[a]	X		
	4 h[a]	X	4 h	X[b]
	8 h[a]	X	8 h	X[b]
	12 h[a]	X	12 h	X[b]

a Relative to start of infusion
b Relative to time of dose of AZD6244
c Within 10 minutes prior to dosing
d This sample should be taken within 10 minutes prior to dosing with chemotherapy (assuming AZD6244 was administered on Day –3, this should correspond to 72h post AZD6244 dose)

3.3 Rationale and risk/benefit assessment

3.3.1 Rationale for study design, doses and control groups

This study is a Phase I, open-label, dose-finding study. It is designed to explore twice-daily dosing with AZD6244, a MEK inhibitor, in combination with select chemotherapies in patients with advanced solid tumors. The study will evaluate the safety profile, tolerability and PK of AZD6244 in combination with docetaxel, dacarbazine, erlotinib, and temsirolimus. The PK of the chemotherapies in combination with AZD6244 will also be investigated.

AZD6244 has demonstrated a good safety profile in preclinical toxicology studies. Based on these preclinical studies and preliminary clinical trial safety results, along with the expectation of minimal PK interaction, it is believed that AZD6244 will have an acceptable safety and tolerability profile for administration with established chemotherapies (Section 1.1.2).

This study has been designed using a conservative dose escalation approach taking into account the information already available from the mix and drink formulation (Study ARRY-401) and the declared MTD of the capsule formulation of AZD6244 study 5 (D1532C00005), which is 75 mg BD. The starting dose of this study is 50 mg BD, or 66% of the MTD of Study 5.

Cohorts of 3 to 6 patients will be recruited during the escalation phase to allow for early discontinuations and the observation of a minimum of 3 evaluable patients per cohort, as the underlying DLT of this advanced population in the combination setting is unknown.

This approach is justified by the fact that the dose escalation will commence in the region of pharmacological activity based on the clinical information to date.

The combination of AZD6244 with each of docetaxel, erlotinib and temsirolimus is being investigated in this study as data from preclinical studies into anti-tumor effects of the combination of AZD6244 with the same agent (docetaxel) or same pharmacological targets (gefitinib and rapamycin respectively) suggests an increased anti-tumor activity with the combination when compared to either agent alone.

The dacarbazine combination has been selected to investigate the potential additive effect of dacarbazine, a standard of care chemotherapy for advanced melanoma and AZD6244 which, as monotherapy, has also demonstrated some anti-tumor activity in advanced melanoma. AZD6244 is a targeted agent, a MEK inhibitor, and the hypothesis is that the efficacy of the two agents in combination will be at least additive.

In study D1532C00003 (melanoma) there was no apparent difference in efficacy between AZD6244 and temozolomide for the primary endpoint (Progression Free Survival), although anti-tumor activity was detected. Neither study D1532C00011 (CRC) or study D1532C00012 (NSCLC) reached their primary endpoint of delaying disease progression versus the randomized comparators in the overall population, however, there was evidence of anti-tumor activity in the NSCLC study. Study D1532C00008 is currently ongoing and results are not available. The information from this study will be used to determine the dose(s) of BD

AZD6244 that can be combined with standard doses of docetaxel, dacarbazine, erlotinib, and temsirolimus for various solid tumors.

The 100mg dose of erlotinib was chosen because it is the dose licensed for use in combination therapy. While temsirolimus has no licensed combination dose and, as theoretically overlapping toxicities are not expected, the standard monotherapy dose of 25mg/week will be utilized in the first instance and adjusted if clinically indicated to 15mg/week.

In order to investigate whether there is any effect on PK, blood samples will be collected after administration of AZD6244 alone, chemotherapy alone, and AZD6244 and chemotherapy together. Initially the docetaxel, dacarbazine, erlotinib and temsirolimus arms will differ, with the aim of maximizing the chance of sufficient washout between treatments (to minimize the risk of carry over between PK sampling days) whilst minimizing study duration. Due to the long half-lives of erlotinib (and requirement to dose to steady state) and temsirolimus (and the active metabolite sirolimus), in these arms the PK of AZD6244 alone will be assessed after a single dose administration prior to chemotherapy to prevent the need for a lengthy washout period. As temsirolimus (and the active metabolite sirolimus) is preferentially distributed into red blood cells, whole blood samples rather than plasma samples will be collected and analysed for concentration determination of temsirolimus/sirolimus.

3.3.2 Risk/benefit and ethical assessment

There is a great need for new regimens improving the efficacy of conventional cytotoxic anti-cancer regimens. Cell signalling through growth factor receptors and protein kinases plays an essential role in cell survival, proliferation, and differentiation. MEK is a critical kinase in the MAP signal transduction pathway that provides growth signals to cancer cells. Targeted antibodies and small molecule inhibitors of cell signalling through either HER1 or HER2 or VEGF-related pathways have been shown to be efficacious when combined with standard chemotherapy therapy.

Preliminary reproductive toxicology data indicate that AZD6244 can have adverse effects on embryofetal development and survival at dose levels that do not induce maternal toxicity in mice. Subsequently, AZD6244 should not be administered to pregnant or breast-feeding women and conception while on treatment must be avoided. Male patients with sexual partners who are pregnant or who could become pregnant (i.e., women of child-bearing potential) should use acceptable methods of contraception (See Section 3.4.5) for 16 weeks after completing the study to avoid pregnancy and/or potential adverse effects on the developing embryo.

In pre-clinical studies, rats receiving multiple doses of AZD6244 showed evidence of gastric mucosal mineralization that appeared to increase with dose. Mineralization was also seen in multiple tissues (cornea, kidney, liver, myocardium, skeletal muscle, glandular stomach) in mice dosed with AZD6244 Hyd-Sulfate for up to one month. Tissue mineralization was not apparent in cynomolgus monkeys dosed for up to one month with either formulation of AZD6244. In mice and rats, tissue mineralization was associated with changes in plasma inorganic phosphate and albumin, and in mice, with changes in calcium. Monitoring of serum

calcium and phosphorus levels in patients is included in the study plan. Although toxicology studies in rodents have shown evidence of distension of the urinary bladder and swelling in the bulbocavernous muscle, to date there has only been only one adverse event of urinary retention reported and this was not suspected to be an adverse drug reaction. It is anticipated that adverse events involving the bladder or penis would be reported rapidly by the patients to the Investigators, before it became clinically apparent on the regular clinical examinations already factored into the study design.

There are preclinical xenograft data to suggest that some tumors may be responsive to treatment with AZD6244. In addition there are data that suggest that AZD6244 given with standard anti-cancer therapy may be superior to either agent given alone. Amongst the combinations with standard cytotoxic drugs, one of the most striking effects was seen with AZD6244 and docetaxel (See Table 1).

In a AZD6244 Phase I study (ARRY-0401) involving patients with a variety of advanced cancers who had failed all previous treatments or had no standard treatment available for their cancer, of the patients who had at least 1 pre-and post-dose measurement of target lesions recorded, SD was observed in 17 patients. Long term (≥5 months) SD was observed in 9 patients and 2 patients (now progressed) had SD with duration of 16 and 20 months, respectively, as of January 2007.

The study population for the current protocol consists of those patients who would be eligible for each treatment as a standard of care or those who may derive benefit from the combination therapies (advanced disease or for which no recognized standard therapy exists). Since patients with potentially curative options will be excluded, patients may not achieve a clinical benefit from participating in the research. In order to enhance the safety of participating patients, we are incorporating into the study design close monitoring of all safety parameters and ongoing PK analysis in order to guide the dose escalation decisions. Further details of particular safety monitoring are given in the remainder of this section.

Dermatological adverse events have been the most frequently reported adverse event occurring in the ongoing clinical program. Rash was also reported by 49% of patients in a Phase I-II trial of another MEK inhibitor PD-0325901, reported at the American Society of Clinical Oncology in 2005 (LoRusso et al 2005). See rash management guidance Section 3.2.1.1

In the ongoing clinical program with AZD6244 free-base and Hyd-Sulfate, diarrhea, nausea and vomiting have been frequently reported AEs. The majority of episodes have been self-limiting or easily managed with simple anti-emetic and anti-diarrhea medication such as prochlorperazine and loperamide. Investigators are advised to monitor for signs of such GI side effects in patients receiving AZD6244.

In a preliminary assessment of laboratory data in the ongoing clinical program, there appeared to be a trend towards elevated liver transaminase values over the first 28 days of BD dosing with AZD6244. The majority of transaminase elevations reported either remained within

normal limits or did not increase by more than a single CTCAE grade after the first month on treatment. Regular measurements of liver function tests are included in the study plan.

Other AEs that have been reported frequently in the Phase I study (ARRY-0401) include fatigue, peripheral edema, and dyspnea. The underlying cause of the edema adverse events is uncertain at present. All cardiorespiratory adverse events will be followed up with chest X-ray, triplicate ECGs and oxygen saturation assessments. Within this study baseline b-type (Brain) natriuretic peptide (BNP) or N-terminal prohormone b-type natriuretic peptide (NT-proBNP) and echocardiogram/MUGA scans, with follow up assessments for echocardiogram/MUGA at 6 weeks, after 3 months, then every 3 months while receiving of treatment with AZD6244. Blood samples for BNP or NT-proBNP will be collected at screening and at the start of each new cycle. Additional assessments for echocardiogram/MUGA and BNP or NT-proBNP at the occurrence of cardiac adverse events are requested in order to assist the evaluation of potential underlying mechanisms for edema symptoms. O_2 saturations will be measured as part of the vital sign assessments. Respiratory adverse events such as hypoxia (a drop in O_2 saturation considered clinically significant) will be investigated by full PFT and HRCT scans.

Visual disturbance AEs have been reported in two ongoing clinical studies (D1532C00003 and D1532C00008). There were no specific clinical findings reported from those patients that underwent an ophthamologic evaluation after reporting a visual disturbance adverse event. Full ophthalmologic examinations will be performed at baseline, once between 4 and 6 weeks of dosing, and ad hoc on the occurrence of any visual disturbance AE in order to document any effects that may be linked to the administration of AZD6244.

The AZD6244 Hyd-Sulfate capsule formulation contains Vitamin E as an excipient. High doses of Vitamin E have been reported to potentiate the anticoagulant activity of coumarins such as warfarin. Therefore, patients who are taking coumarin anticoagulants during the study will be advised to increase the frequency of INR measurements upon starting on AZD6244. In addition the intake of excessive doses of Vitamin E is also restricted during this study.

Given the extensive safety monitoring included in this study, and inclusion of a full review of available safety, tolerability and pharmacokinetic data prior to each dose escalation decision, AstraZeneca believes that the overall risk for the patients who participate in this study would be acceptable.

3.4 Selection of study population

3.4.1 Study selection record

Patients with advanced solid tumors for whom the selected standard chemotherapy regimens represents a standard of care or those who may derive benefit from the combination therapies would be considered appropriate therapy and who also satisfy the inclusion and exclusion criteria for the selected chemotherapy treatments. Please see product labels for each agent. The Investigator must ensure that patients meet ALL eligibility requirements.

Patients MUST meet the following criteria prior to dosing on Day 1 for docetaxel and dacarbazine and day –8 to –3 for erlotinib and temsirolimus. Exceptions will NOT be granted.

Investigator(s) must keep a record of patients who were considered for enrollment but never enrolled e.g., patient screening log, according to local procedures. This information is necessary to establish that the patient population was selected without bias.

3.4.2 Inclusion criteria

For inclusion in the study patients must fulfill all of the following criteria:

1. Provision of written informed consent
2. Male or female, aged 18 years or older
3. Patients will have measurable and/ or non-measurable disease, lacking curative options for whom the selected chemotherapy represents a standard of care or those who may derive benefit from the combination therapies
4. WHO performance status 0-1
5. Evidence of post-menopausal status or negative urine/serum pregnancy test for pre-menopausal female patients
6. Patients must have calculated serum creatinine >50ml/min (using Cockcroft-Gault formula or by 24 hour urine collection
7. Patients must be able to swallow AZD6244 capsules

Optional biomarker and/or host genetics research:

For inclusion in the biomarker and/or genetic research component of the study, patients must provide separate written informed consent. If a patient declines to participate in biomarker and/or host genetics research, there will be no penalty or loss of benefit to the patient. A patient who declines biomarker and/or host genetic research participation will not be excluded form any other aspect of the main study as described in this clinical study protocol.

3.4.3 Exclusion criteria

In addition to those given below, all exclusion criteria as stated in the individual product labels for each chemotherapy will apply. Patients must be excluded from this study according to local prescribing instructions. See Appendices for product labels of standard chemotherapies used in this study.

Any of the following is regarded as a criterion for exclusion from the study:

1. Prior treatment with a MEK inhibitor

2. Previous enrollment or assignment to treatment in the present study

3. Involvement in the planning and conduct of the study (applies to both AstraZeneca staff or staff at the study site)

4. Having received an investigational drug within the 30 days prior to entry or patients who have not recovered from side effects of an investigational study drug

5. Recent major surgery within 4 weeks prior to consent (excluding the placement of vascular access) which would prevent administration of standard chemotherapy

6. Radiotherapy or standard chemotherapy within 21 days prior to entry into the study (not including palliative radiotherapy at focal sites)

7. Brain metastases or spinal cord compression unless treated and stable (for at least 1 month) off steroids

8. Evidence of active infection or active bleeding diatheses

9. Refractory nausea and vomiting, chronic gastrointestinal diseases (e.g., inflammatory bowel disease), or significant bowel resection that would preclude adequate absorption

10. Patients with factors that increase the risk of QT prolongation or arrhythmic events (e.g., heart failure, hypokalemia, family history of long QT interval syndrome) or QTc interval of > 450ms for males or > 470ms for females on screening

11. Evidence of severe or uncontrolled systemic disease (e.g., severe hepatic impairment, severe renal impairment uncontrolled diabetes, acute uncontrolled infection) or current unstable or uncompensated respiratory or cardiac conditions or peripheral vascular disease including diabetic vasculopathy, or renal transplant

12. Patients with documented cases of human immunodeficiency virus (HIV) or active hepatitis B or C infection

13. Laboratory values as listed below:

    - Absolute Neutrophil Count (ANC) <1500 per mm^3
    - Platelets <100,000 per mm^3
    - Hemoglobin (Hgb) <9.0 g/dL
    - Serum bilirubin ≥1.5x Upper Limit of Normal (ULN) (known Gilbert's disease is allowed)

- Aspartate aminotransferase (AST/SGOT) or alanine aminotransferase (ALT/SGPT) ≥2.5 x ULN or 5 x ULN if liver metastases
- Patients with proteinuria > 2gr/24hr urine collection are excluded (See Section 4.8.1.2

14. Clinical judgment by the Investigator that the patient should not participate in the study
15. Known hypersensitivity to the standard chemotherapy proposed for the patient.
16. Cardiac conditions as follows:
    - Uncontrolled hypertension (BP150/95 despite optimal therapy)
    - Heart failure NYHA Class II or above
    - Prior or current cardiomyopathy
    - Baseline LVEF ≤ 50%
    - Atrial fibrillation with heart rate >100bpm
    - Unstable ischemic heart disease (MI within last 6 months, or angina requiring use of nitrates more than once weekly)
17. Use of strong CYP1A2 or 3A4 inducers and/or inhibitors (for example, but not limited to, ketoconazole, rifampacin, atazanavir, clarithromycin, indinavir, itraconazole, nefazodone, nelfinavir, ritonavir, saquinavir, telithromycin, troleandomycin (TAO), voriconazole, grapefruit or grapefruit juice, ifabutin, rifapentine, phenytoin, carbamazepine, phenobarbital and St. John's Wort)
18. Current smokers or use of any tobacco (erlotinib arm only)

For exclusion criteria related to participation in the a) optional host pharmacogenetics, see Appendix D and /or b) biomarker research please refer to Appendix E

3.4.4 Protocol precautions

Concomitant medication that is known to prolong QT interval should be used with caution.

3.4.5 Restrictions

1. Reliable methods of contraception should be used consistently and correctly. Acceptable methods include implants, injectables, combined oral contraceptives, (which must all be combined with barrier methods of contraception), some IUDs, sexual abstinence and vasectomized partner.

Preliminary reproductive toxicology data indicate that AZD6244 can have adverse effects on embryo fetal development and survival at dose levels that do not induce maternal toxicity in mice. Patients who are pregnant or actively breast feeding are not eligible to participate in this study. Female patients of child bearing potential will be required to use reliable methods of contraception for the duration of the study and until 4 weeks after the last dose of study treatment.

Male patients will be required to use reliable methods of contraception for the duration of the study and until 16 weeks after the last dose of study treatment.

2. Patients should not take Vitamin E supplements or multivitamin supplements that provide a total daily dose in excess of 100% of the recommended daily dose of Vitamin E.

3. Patients who are taking coumadin anticoagulants (e.g. warfarin) should have their anticoagulation tested more frequently while taking AZD6244.

4. Throughout the study, patients should avoid changes to or the addition of all concomitant medications, in particular any that are likely to affect the metabolism of AZD6244 (e.g., CYP1A2 or 3A4 inducers), or the chemotherapy, unless considered clinically essential for management of concurrent conditions.

5. Grapefruit juice and Seville orange juice must not be consumed while participating in the study.

3.4.6 Discontinuation of patients from treatment or assessment

3.4.6.1 Criteria for discontinuation

Patients may be discontinued from study treatment and assessments at any time. Specific reasons for discontinuing a patient from this study are:

- Voluntary discontinuation by the patient, who is at any time free to discontinue his/her participation in the study without prejudice to further treatment
- Safety reasons as judged by the investigator and/or AstraZeneca
- Adverse Events
- Severe non-compliance to protocol as judged by the investigator and/or AstraZeneca
- Pregnancy in a female patient
- Incorrect enrollment i.e., the patient does not to meet the required inclusion/exclusion criteria for the study. See Section 3.4.6.5 details regarding incorrect enrollment.

- Patient lost to follow-up
- Disease progression (per objective progression radiographically per RECIST or clinical/subjective progression per investigator)

3.4.6.2 Withdrawal of consent for optional biomarker and/or host genetics research

A patient may withdraw from the optional biomarker and/or host genetic research at any time, independent of any decision concerning participation in other aspects of the main study described in this protocol. Voluntary discontinuation by the patient will not prejudice further treatment. For further details see Appendix D and E.

3.4.6.3 Procedures for discontinuation from treatment or assessment

Per Section 3.1.3, patients may continue to receive AZD6244 in combination with prescribed chemotherapy until progression or as long as they do not experience dose-limiting toxicity and are in the opinion of the investigator, continuing to derive benefit. Patients may also continue AZD6244 alone after prescribed chemotherapy has been completed if the investigator believes they are continuing to derive benefit from AZD6244 treatment.

Once the prescribed chemotherapy has been stopped, the reason for discontinuation from prescribed chemotherapy will be recorded on the CRF; if the patient continues on AZD6244 alone, study assessments will continue per study plan until discontinuation of AZD6244

Once AZD6244 is permanently discontinued, regardless of whether the prescribed chemotherapy is completed, the patient should be asked about the reason(s). and asked about any adverse events. If possible, they should be seen and assessed by an investigator(s). Adverse events should be followed up and the patient should return any investigational products.

At discontinuation from AZD6244, all on-going study-related toxicities and SAEs must be followed until resolution, unless in the investigator’s opinion, the condition is unlikely to resolve due to the patient’s underlying disease.

All patients who have new or worsening CTCAE grade 3 or 4 laboratory values at the time of discontinuation from AZD6244 must have further tests performed and the results recorded on the appropriate Case Report Form (CRF) until the laboratory values have returned to CTCAE grade 1 or 2; unless these values are not likely to improve because of the underlying disease. In such cases, the investigators must record their opinions in patient’s medical records. Laboratory abnormalities should not be reported as adverse events unless any criterion for a serious adverse event (SAE) is fulfilled, the laboratory abnormality causes the patient to discontinue from AZD6244 or the investigator insists the abnormality should be reported as an AE.

After discontinuation from AZD6244, patients must be followed up for all existing and new AEs for 30 calendar days after the last dose of study drug. All new AEs occurring during that period must be recorded (if SAEs they must be reported to AstraZeneca within 24 hours) and

followed up for resolution as above. After the 30 day follow-up period, no further assessments will be required and patient participation in the study will end.

3.4.6.4 Procedures for discontinuation from optional biomarker and/or host genetic aspects of study

Procedure for discontinuing participation in the optional biomarker and/or host genetic aspects of this study are outlined in Appendix D and E.

3.4.6.5 Procedures for handling incorrectly enrolled patients

Patients not meeting the inclusion/exclusion criteria for a study should, under no circumstances, be enrolled into the study - there can be no exceptions to this rule.

Where patients not meeting the study criteria are enrolled in error, or where patients subsequently fail to meet the criteria for the study post enrollment, the investigator, will in conjunction with AstraZeneca, discuss whether such a patient should withdraw from study drug taking into consideration ethical and safety factors. The discussion and rationale for each decision will be documented. Procedures for handling incorrectly enrolled patients will be documented in the Statistical Analysis Plan.

3.5 Treatment(s)

3.5.1 Investigational product(s)

3.5.1.1 Identity of investigational product

AZD6244 drug product is supplied as a capsule and will be provided the AstraZeneca Investigational Products Supply department.

AZD6244 capsule formulation drug product is supplied as 25 mg capsules in high-density polyethylene (HDPE) bottles.

The standard chemotherapies will be supplied and prepared by the clinical study sites according to standard practice. AstraZeneca will reimburse study sites for study drugs used for patients where the drug given in combination does not represent the standard of care.

Table 11 Identity of investigational product

Investigational product	Formulation	Dosage form and strength	Manufacturer	Formulation number	Batch number
AZD6244	Hyd-Sulfate[b]	25 mg	AstraZeneca	F013484	Tbc[a]

[a] Batch numbers for all investigational products will be identified in the clinical study report
[b] Hyd-Sulfate capsule formulation

3.5.1.2 Labeling

Each bottle of AZD6244 Capsules will be labeled by investigational product supplies (IPS), AstraZeneca.

All labels will comply with good manufacturing practice (GMP) regulations, and will state that the drug is for clinical use only and should be kept out of reach of children. Information regarding the patient, for example enrollment number (E code), contents of the bottle, expiry date, dosing instructions as well as a space for the date of dispensing will be included on the labels.

The AZD6244 bottle will have a tear-off portion on the label which will be removed at the time of dispensing and attached to the case report form (CRF). Patient accountability CRFs will be monitored by the AstraZeneca representative.

3.5.1.3 Storage

All investigational products must be kept in a secure place under appropriate storage conditions. A description of the appropriate storage and shipment conditions are specified on the investigational product bottle label. The site staff will also insure that patients are instructed regarding proper storage and handling of the capsules.

3.5.1.4 Accountability

The investigational medication provided by AstraZeneca for this study is for use only as directed in the protocol. The investigator or delegate must maintain accurate records accounting for the receipt of the investigational material as well as for the dispensation of the material. AZD6244 must be accounted for and reconciled at the patient level and he lot/batch number must be recorded on the appropriate CRF. Any unused AZD6244 capsules (expired, returned, etc.) will be monitored by AstraZeneca or an agent of AstraZeneca and will be returned to as per AZ standard procedures. All commercially available chemotherapies used for the study must be accounted for per the institutions' standards and as per ICH guidelines and GCPs.

3.5.2 Doses and treatment regimens

3.5.2.1 Capsule formulation

AZD6244 capsule formulation drug product is supplied as 25 mg capsules in high-density polyethylene (HDPE) bottles.

If patients are not able to tolerate AZD6244, dose reductions are permitted as described in Section 3.2.1. Reduced doses should be in 25mg decrements since that is the only strength available.

Patients will be instructed as to when and how many capsules to use each day.

Study sites will ensure that patients receiving AZD6244 are compliant with treatment.

3.5.2.2 AZD6244 Dosing

On clinic days, dosing and breakfast should be delayed until arrival at the clinic. Dosing should not occur until pre-dose blood samples and other study procedures have been completed. The morning dose should be taken in the fasted state. Breakfast can be given from 1 hour following dosing.

For BD dosing at home (out of clinic):

- The morning dose should be taken in the fasted state. Breakfast can be taken from 1 hour following dosing.

- Evening doses should not be taken in the 1 hour preceding a meal or in the 2 hours after having finished a meal.

The doses should be taken approximately 12 hours apart for example 08:00h and 20:00h or 09:00h and 21:00h.

3.5.2.3 Dosing of standard chemotherapy either alone or in combination with AZD6244

For docetaxel and dacarbazine, AZD6244 will be initiated on day 3 of the first cycle and will be given continuously on a twice-daily basis thereafter. For erlotinib and temsirolimus, patients will receive a single dose of AZD6244 three to eight days prior to Cycle 1/Day 1 and then AZD6244 will be given continuously on a twice daily basis beginning on Day 8 of the first cycle. The single dose will be the dose of the cohort.

When patients are scheduled to receive AZD6244 and chemotherapy in the clinic, they should be given at the same time.

- Docetaxel: 75mg/m^2 IV infusion over 60 minutes on day 1 of each 21 day cycle

- Dacarbazine: 1000mg/ m^2 IV infusion over 60 minutes on day 1, of each 21 day cycle.

- Erlotinib: 100mg oral daily continuously starting on Cycle 1/Day 1Temsirolimus: 25 mg IV infusion over 60 minutes on days 1, 8, and 15 of each 21 day cycle

The temsirolimus dose may be reduced to 15mg if the DLT(s) observed for the cohort is/are a known side effect of temsirolimus. Likewise, if the DLT(s) is/are a known side effect of AZD6244, than the AZD6244 dose will be reduced accordingly. All dosing decisions will be at the discretion of the SRC who will review the emerging tolerability and safety profile on an ongoing basis, and on completion of each cohort dose level, and decide whether to continue at 25mg of temsirolimus or reduce to 15 mg for subsequent cohorts.

Please refer to the applicable Product labels in the protocol appendices for product details and complete instructions regarding administration of selected chemotherapies. (See Appendix F

for docetaxel, Appendix G for dacarbazine, Appendix H for erlotinib and Appendix I for temsirolimus). For the first cycle of treatment, patients should remain in the treatment area for a minimum of 4 hours following completion of their chemotherapy dosing in the event that acute symptoms, e.g., respiratory distress, severe skin reactions, abdominal cramping or diarrhea develop.

The product label instructions regarding administration of erlotinib at least one hour before or two hours after the ingestion of food must be adhered to.

3.5.3 Method of assigning patients to treatment groups

After a patient has given written informed consent, the site will assign an enrollment code (E-code) and contact the AstraZeneca representative. AstraZeneca will confirm patient registration. The E-code is a unique 7-digit number made up of a 4-digit center number and a 3-digit patient number (E00NNXXX: NN being the center number, XXX being the patient enrollment number at the center). Enrollment numbers will start at 001 in each center and increase in numerical order (e.g., at center 0201, patients will be assigned E-codes E0201001, E0201002, E0201003, etc). This number is the patient's unique identifier and is used to identify the patient on the Case Report Forms (CRFs). If a patient discontinues from study treatment, the patient number will not be reused. Patient will not be allowed to re-enter the study.

3.5.4 Blinding and procedures for unblinding the study

This is an open-label study.

3.5.5 Concomitant medication

Other medication, which is considered necessary for the patient's safety and well-being, may be given at the discretion of the investigator(s). The administration of all medication (including investigational products) must be recorded in the appropriate sections of the CRF. Trade name, generic name, indication, route, and dates of administration should be properly documented.

No other standard cancer agents, or investigational drugs should be administered while patients are receiving study medication. Supportive treatments including hormonal and bisphosphonate therapies are allowed. Refer to exclusion criteria in Section 3.4.3 and restrictions in Section 3.4.5.

3.5.6 Treatment compliance

Patients should be carefully instructed regarding their study treatment. Instructions should include proper storage and handling of the AZD6244 capsules as well as instructions for use of any standard cancer treatments and supplies, and the return of any unused medication. All capsules should be counted to check for compliance. Discrepancies between the number of capsules returned and the expected number of capsules returned should be discussed with the patients and the reasons for non-compliance documented.

4. MEASUREMENT OF STUDY VARIABLES

The primary study variable is safety. The following study measurements will be obtained at various time points throughout the study: vital signs (including weight), safety assessments: hematology, urinalysis, ECG, ophthalmologic exam, and other safety labs including: BNP and Troponin I, MUGA scans/ echocardiograms, and incidence and CTCAE grade. The times of these measurements are detailed in the study plans (Section3.2.3). The following 'priority order' will be in effect when more than one assessment is required at a particular time point:

- ECG
- Vital signs (including weight and body temp ^{o}C)
- PK collection (s)
- O_2 saturation
- Physical examination
- Safety assessments (clinical chemistry, hematology, urinalysis, etc)
- MUGA/echocardiogram
- Ophthalmologic examination

4.1 Medical examination and demographic measurements

4.1.1 Enrollment medical examination and demographic measurements

Each patient will undergo an enrollment medical examination within the 21 days prior to the treatment unless otherwise specified in the study plan. This will consist of:

- Provision of written informed consent
- Consent for optional tumor and plasma samples for biomarker analysis and blood sample for pharmacogenetics
- Recording of demographic data – date of birth, sex, height, weight, race
- Standard medical and surgical history and a physical examination including full ophthalmologic exam.
- WHO performance status
- Blood sample for standard clinical chemistry and hematology assessments (temsirolimus arm only will have fasting glucose, cholesterol, and triglycerides)

- Sample for urinalysis
- 24 hour urine for total protein (temsirolimus arm only)
- Resting supine blood pressure (BP) and pulse rate
- ECG
- MUGA Scan or echocardiogram
- Smoking status
- Recording of any concomitant medications.
- Date of diagnosis of tumor, site of primary lesion, date of most recent recurrence, locally advanced or metastatic site(s)
- Previous cancer treatments
- Urine or Serum pregnancy test for pre menopausal female patients
- Chest X-ray
- O_2 saturation
- Baseline HRCT (erlotinib and temsirolimus treatments only)
- Provision of archival tumor sample
- Adverse event assessment
- Baseline tumor assessment according to the Response Evaluation Criteria In Solid Tumors (RECIST) (see Appendix C) for patients with measurable disease or other assessment for patients with non-measurable disease.

4.1.2 Post-study medical examination

At the time of AZD6244 discontinuation the following assessments will be made:

- Physical examination
- Vital signs (including weight, blood pressure, pulse rate, and body temperature)
- Ophthalmologic examination
- Clinical Chemistry/Hematology/Urinalysis

- Tumor Assessment (unless performed within the last 4 weeks)
- Adverse Events
- Concomitant Medications

AEs/SAEs/abnormal labs that are not recovered should be followed until resolution or baseline, or in cases of persistence, in the opinion of the investigator, further resolution is unlikely. For further information see Section 4.8.1.2

4.2 Pharmacokinetic measurements

For the complete PK sampling for individual arms see tables in Section 3.2.3.

4.2.1 Determination of drug concentration in biological samples

Blood samples for determination of plasma concentrations of AZD6244, N-desmethyl AZD6244 and/or docetaxel/dacarbazine (and the major circulating metabolite AIC)/erlotinib and/or determination of concentrations in whole blood for temsirolimus (and the active metabolite sirolimus) will be taken at the times specified in Section 3.2.3. If warranted, the blood samples may also be used for analysis of additional metabolites. Depending on emerging data/information, the timings and number of the PK samples may be altered, but the maximum total blood volume will not be exceeded (Section 4.7). The actual sample time and date of all PK samples must be recorded in the CRF. Further details for sample collected and shipment will be provided in the Laboratory Manual for Investigators.

4.2.2 Collection of biological samples

Analysis of plasma/blood samples for the determination of AZD6244, N-desmethyl AZD6244 and chemotherapy concentrations will be the responsibility of the

Venous blood samples will be collected to provide plasma for analysis, except for temsirolimus (and the active metabolite sirolimus) where analysis in whole blood will be required.

Labels for tubes are unique, enabling identification of the study, compound to be analyzed, patient/center number, visit number and nominal time point.

Samples will be shipped initially to the coordinating laboratory, and subsequently sent for analysis to a referral laboratory. A sample inventory must accompany each shipment of samples.

Details of sample collection, storage and shipment will be provided in the Laboratory Manual for Investigators.

4.3 Pharmacodynamic measurements

4.3.1 Collection of archived tumor and plasma samples for biomarker analysis

It is preferred to enroll patients with archived tumor tissue available for biomarker analysis. However, inability to provide such tissue is not an exclusion criterion.

In consenting patients, an archived tumor specimen and plasma sample will be obtained pre-dose from all patients for assessment of RAS and RAF mutation status. The Principal Investigator will be asked to provide archival tissue. Other biomarkers may be assessed as driven by emerging biological understanding. Such biomarkers may include analysis of genetic variability of response related genes, gene expression profiling, protein expression profiling etc. See Appendix E for further details

Information regarding the collection, storage and shipping of samples will also be provided in the laboratory manual.

4.4 Safety measurements

4.4.1 Laboratory safety measurements

Blood and urine samples for determination of clinical chemistry and hematology parameters will be taken at the times given in the study plans (Section 3). The date of collection will be recorded on the appropriate CRF. Additional laboratory or safety parameters may be added based upon Investigator’s discretion/clinical judgment or based on individual product label requirements for docetaxel, dacarbazine, temsirolimus or erlotinib).

The following clinical chemistry, urinalysis and hematology tests will be performed:

Table 12 Clinical chemistry, hematology, and urinalysis

**Clinical chemistry**	**Hematology**
s - Albumin	b - Erythrocyte count
s - Alanine aminotransferase (ALT)	b - Hemoglobin
s - Aspartate aminotransferase (AST)	b - Hematocrit
s - Alkaline phosphatase (ALP)	b - Platelet count
s - Total Calcium	b - Leukocyte cell count
s - Creatinine	b - Leukocyte differential count (absolute count)
s – Creatinine clearance, calculated	b - Basophils
s - Gamma glutamyltranserase (γGT)	b - Eosinophils
s - Glucose[a]	b - Lymphocytes
s - Magnesium	b - monocytes
s - Phosphate	b - Absolute Neutrophil Count (ANC)

Table 12 Clinical chemistry, hematology, and urinalysis

Clinical chemistry	Hematology
s - Potassium	
s - Sodium	
s - Total protein	
s - Total bilirubin	
s - Urea nitrogen	
s/p- Troponin I	
s/p - BNP or NT-proBNP	
s - Cholesterol[a]	
s - Triglycerides[a]	
Urinalysis	u-Ph
u-Glucose	u-Blood
u-Ketones	u-Bilirubin
u-Protein (qualitative)	u-Microscopy (red blood cells and white blood cells, bacteria, casts and crystals) only perform as clinically indicated

a Temsirolimus arm will have fasting glucose, cholesterol and triglycerides at screening. Cholesterol and triglycerides will be repeated only as clinically indicated

4.4.2 Pregnancy test

Pregnancy tests for pre-menopausal female patients will be preformed at screening and at the start of each new cycle. Females must give a sample of the first urine passed or a blood sample within 72 h of Cycle 1/Day 1(docetaxel and dacarbazine) or 72 hours before first receiving AZD6244 (erlotinib and temsirolimus) for a pregnancy test to be performed. The date of collection will be recorded on the appropriate CRF. See study plans for schedule details (Section 3.2.3).

4.4.3 Electrocardiographic measurements

All ECGs will be analyzed locally. Twelve-lead ECGs will be obtained after the patient has rested in the supine position for 10 minutes in each case. All study participants will have screening ECGs performed in triplicate (one immediately after the other) according to standard practice. Subsequent ECGs should be preformed according to the study plan of the individual therapy arm. Triplicate 12-lead ECGs are also required at discontinuation from AZD6244 and at any cardiorespiratory adverse event.

The Investigator should review the paper copy of the ECGs on each study day and may refer to a local cardiologist if appropriate. Any symptoms from the patient should be registered as a comment and if AE criteria are met, recorded as an AE.

4.4.4 Physical examination

A physical examination (PE) including weight and calculation of BSA (erlotinib and temsirolimus patients do not need a BSA) will be performed during screening and pre-dose of each new cycle, according to study plans (Section 3.2.3). Only findings considered abnormal on PE should be detailed on the appropriate CRF, e.g. medical history, AE, etc. BSA must be confirmed prior to administration of chemotherapy, details will be recorded in CRF.

4.4.5 Vital signs

See study plans for times of vital sign assessments. Applicable symptoms should be registered as AEs.

4.4.5.1 Blood pressure and pulse rate

On each visit, resting, supine blood pressure and pulse rate will be measured using a semi-automatic blood pressure recording device with an appropriate cuff size. For timing of individual measurements refer to study plans.

4.4.6 Other safety measurements

4.4.6.1 MUGA scan/echocardiogram

A MUGA or echocardiogram will be conducted pre-dose (within 4 weeks prior to first dose) and after 6 weeks at 3 months, and every 3 months thereafter while on study therapy. Further scans are required should adverse events suggestive of cardiac dysfunction occur at any other time while on treatment and up to 30 days after discontinuation.

The modality of the cardiac function assessments must be consistent within patient, i.e., if echocardiogram is used for the screening assessment then echocardiogram should also be used for all subsequent scans as required.

Please refer to Figure 3, Left ventricular ejection fraction (LVEF) algorithm for asymptomatic changes. If the patient becomes symptomatic, refer for a cardiology consult.

4.4.6.2 BNP

Either form of BNP will be tested locally according to local practice at the same time as safety labs. In addition an unscheduled sample should be taken as clinically warranted, e.g., if there are adverse events with a possible cardiac etiology such as dyspnea or edema.

The modality of these blood assessments must be consistent within patient, i.e., if BNP is used for the screening assessment then BNP should also be used for subsequent assessments.

4.4.6.3 Troponin I

Tested locally according to local practice at the same time as safety labs. In addition an unscheduled sample should be taken as clinically warranted, e.g., if there are adverse events with a possible cardiac etiology such as dyspnea or edema.

4.4.6.4 O_2 saturation measurement

A baseline measurement of oxygen saturation, performed by pulse oximetry, will be recorded at screening or at any time prior to dosing on Day –3 to –8 or Cycle 1/Day 1; and then as clinically required. Results must be recorded in the CRF.

O_2 measurements will be repeated as clinically indicated as part of the routine management of the patient on the occurrence of any cardiorespiratory AEs. If there is a clinically significant drop in O_2 saturation, this event should be followed up with full pulmonary function tests and HRCT scan of the chest.

4.4.6.5 Chest X-ray

A chest X-ray will be performed at screening and on the occurrence of any cardiorespiratory AEs in addition to other assessments as per local standard practice. Results must be recorded in the CRF.

Chest X-rays will be repeated as clinically indicated as part of the routine management of the patient on the occurrence of AEs.

4.4.6.6 Ophthalmologic examination

4.4.6.7 A complete ophthalmologic examination including visual acuity, visual fields, color vision, and a slit-lamp examination must be performed $\leq$ 6 weeks prior to Cycle 1/Day 1 (docetaxel and dacarbazine) and six weeks prior to first dose of AZD6244 (erlotinib and temsirolimus), at week 6, if the patient experiences a visual disturbance AE and at discontinuation from AZD6244. Results must be recorded in the CRF.HRCT

An HRCT will be conducted pre-dose (within 4 weeks prior to first dose) for patients receiving erlotinib and temsirolimus. Further scans are required as clinically indicated or should there be adverse events suggestive of respiratory etiology occur at any other time during treatment and up to 30 days after discontinuation.

4.5 Efficacy measurements and co-variables

4.5.1 Tumor assessment by imaging techniques using RECIST for patients with measurable disease at baseline

The RECIST guidelines for measurable, non-measurable, target and non-target lesions, and the objective tumor response criteria (complete response (CR), PR, SD or progression of disease) are presented in Appendix C. The RECIST criteria will be used to programmatically determine ORR.

Baseline radiological tumor assessments should be performed no more than 4 weeks before the start of study treatment, but should be as close as possible to the start of study treatment.

Following first dose of study treatment on Day 1, efficacy for all patients will be assessed by objective tumor response as detailed in the study plans in Section 3.2.3. Following treatment

on Day 1, efficacy for all patients will be assessed by objective tumor response at baseline, before Cycle 3 and then every other cycle thereafter. Assessments may be performed +/-1 week relative to the specified visit date. In addition, patients who progress between scheduled visits should have a RECIST scan for confirmation.

Baseline CT examination will be performed on anatomical coverage to adequately define all areas of disease. Post-baseline imaging should follow and evaluate all previous identified lesions. MRI should only be used where CT is not feasible or it is medically contra-indicated.

All measurable lesions confirmed and assessed by radiological methods (CT or MRI scans) up to a maximum of 5 lesions per organ and 10 lesions in total, representative of all involved organs, should be identified as target lesions, recorded and measured at baseline, and at the time points specified above.

Localized post-radiation changes may occur and measurable lesions that are in previous irradiation field will not be selected as target lesion.

Non-target lesions will also be monitored throughout the study, and an assessment of non-target lesions will be made and recorded as “present”, “present with progression” or “absent”. Details of any new lesions will also be collected.

If an unscheduled radiological and clinical tumor assessment is performed, and the patient has not progressed, the next scheduled tumor assessment should still be performed at the planned time as detailed in the study plans, Section3.2.3.

Tumor assessment will be performed in accordance with the protocol schedule until evidence of one of the following:

- Progression of disease
- Death without evidence of progression
- Withdrawal of consent
- Discontinuation from AZD6244

A patient will be determined to have progressed if they have progression of target lesions, clear progression of existing non-target lesions, or the appearance of one or more new lesions.

Unequivocal malignant disease not identified prior to starting study treatment on additional anatomical imaging (e.g., computed tomography (CT), magnetic resonance imaging (MRI) or plain X-ray), prompted by symptoms is considered disease progression and should be recorded as new lesions. If progression is uncertain, patients may continue on treatment until the next scheduled assessment or may have an unscheduled assessment earlier than this if considered appropriate by the investigator.

Lesions must be assessed using the same method and technique on each occasion. Lesions will be recorded on the CRF page in the same order as they were recorded at screening. Details of any new lesions will also be collected. If a subject has received palliative radiotherapy to a lesion, that lesion should not be included in assessment of response, but should be assessed for progression.

Categorization of objective tumor response assessment will be based on the RECIST criteria of response: CR (complete response), PR (partial response), SD (stable disease) and PD (progression disease). Response will be calculated in comparison to the baseline tumor measurements obtained before starting treatment. Progression will be calculated in comparison to when the tumor burden was at a minimum.

The sponsor will determine overall best response using the lesion assessments recorded on the CRF.

4.5.2 Objective response rate

4.5.2.1 Objective response rate and method of assessment

Categorization of overall visit response will be based on RECIST using the following response categories: CR, PR, SD, and PD (see Appendix C). In the case of stable disease, measurements must have met the stable disease criteria at least once after start of study treatment for a minimum interval of 6 weeks.

To be assigned a status of PR or CR, changes in tumor assessments must be confirmed no less than 4 weeks after the criteria for response were met.

4.5.3 Tumor assessments for patients with non-measurable disease at baseline

Patients with non-measurable disease should be followed up with the same assessment schedule as those with measurable disease at baseline and as outlined in Section 4.5.

4.5.3.1 Methods of assessment

Assessments of these patients will be under investigators discretion in line with their institutional policies.

4.6 Genetic measurements and co-variables

4.6.1 Collection of samples for genetic testing

Patients will provide a blood sample as per the inclusion criteria and visit schedule. See Appendix D for further details

Genotype is a stable parameter, therefore if for any reason the blood sample is not drawn at Visit 2, it may be taken at any visit until the last study visit. The genetic blood sample should ideally be drawn through the same cannula used to draw blood samples required for the main study.

4.7 Volume of blood sampling

The total volume of blood that will be drawn from each patient in this study is as follows:

Table 13 Volume of blood to be drawn from each patient – Part A & B[a]

Assessment		Sample volume [b] (mL)	No. of samples	Total volume (mL)
**Docetaxel arm[c]**				
PK	AZD6244	2	16	32
	Docetaxel	2	19	38
Safety	Clinical chemistry	7	8	56
	Hematology	3	8	24
	BNP & Troponin	2	2	4
**Dacarbazine arm**				
PK	AZD6244	2	16	32
	Dacarbazine	5	13	65
Safety	Clinical chemistry	7	8	56
	Hematology	3	8	24
	BNP & Troponin	2	2	4
**Erlotinib arm**				
PK	AZD6244	2	18	36
	Erlotinib	4	18	72
Safety	Clinical chemistry	7	6	42
	Hematology	3	6	18
	BNP & Troponin	2	2	4
**Temsirolimus arm**				
PK	AZD6244	2	18	36
	Temsirolimus	3	20	60
Safety	Clinical chemistry	7	6	42
	Hematology	3	6	18
	BNP & Troponin	2	2	4
**All arms**				
Optional genetic sample		9	1	9
Optional plasma sample for biomarker analysis		10	1	10

Table 13 Volume of blood to be drawn from each patient – Part A & B[a]

Assessment	Sample volume [b] (mL)	No. of samples	Total volume (mL)
**Total**			**173-200**

[a] Represents one month on study
b The volume per PK sample may change if required by the bioanalytical method, however a maximum total volume of approximately 200mL will not be exceeded
c PK samples not applicable for Part B

4.8 Adverse Events

The methods for collecting adverse events are described below.

4.8.1 Adverse Events

4.8.1.1 Definitions

The definitions of adverse events (AEs), serious adverse events (SAEs) and other significant adverse events (OAEs) are given below. It is of the utmost importance that all staff involved in the study be familiar with the content of this section. The principal investigator is responsible for ensuring this.

Adverse event

An adverse event is the development of an undesirable medical condition or the deterioration of a pre-existing medical condition following or during exposure to a pharmaceutical product, whether or not considered causally related to the product. An undesirable medical condition can be symptoms (e.g., nausea, chest pain), signs (e.g., tachycardia, enlarged liver) or the abnormal results of an investigation (e.g., laboratory findings, electrocardiogram). In clinical studies, an AE can include an undesirable medical condition occurring at any time, including run-in or washout periods, even if no study treatment has been administered.

Serious adverse event

A serious adverse event is an AE occurring during any study phase (i.e., run-in, treatment, washout, follow-up), and at any dose of the investigational product, comparator or placebo, that fulfils one or more of the following criteria:

- Results in death
- Is immediately life-threatening
- Requires in-patient hospitalization or prolongation of existing hospitalization
- Results in persistent or significant disability or incapacity
- Is a congenital abnormality or birth defect

- Is an important medical event that may jeopardize the patient or may require medical intervention to prevent one of the outcomes listed above?

The causality of SAEs (i.e., their relationship to study treatment) will be assessed by the investigator(s), who in completing the relevant case report form must answer "yes" or "no" to the question "Do you consider that there is a reasonable possibility that the event may have been caused by any of the following – study medication – other medication?". For further guidance on the definition of a SAE and a guide to the interpretation of the causality question, see Appendix B to the Clinical Pharmacology Study Protocol.

Note that SAEs that could be associated with any study procedure should also be reported. For such events the causal relationship is implied as "yes".

Other Significant Adverse Events (OAE)

OAEs will be identified by the Study Delivery Team Physician in consultation with the appropriate Global Drug Safety Physician during the evaluation of safety data for the Clinical Study Report. Significant adverse events of particular clinical importance, other than SAEs and those AEs leading to discontinuation of the patient from study treatment, will be classified as OAEs. Examples of these are marked hematological and other laboratory abnormalities, and certain events that lead to intervention (other than those already classified as serious), dose reduction or significant additional treatment. For each OAE, a narrative may be written and included in the Clinical Study Report.

4.8.1.2 Recording of adverse events

AEs/SAEs will be collected throughout the study, from informed consent until 30 days after study treatment is discontinued.

During the course of the study all AEs and SAEs should be proactively followed up for each patient; events should be followed up to resolution, unless the event is considered by the investigator to be unlikely to resolve due to the underlying disease, or the patient is lost to follow up. Every effort should be made to obtain a resolution for all events, even if the events continue after discontinuation/study completion.

Any SAE or non-serious adverse event considered related to study treatment by the investigator, that is ongoing when the patient completes or discontinues from the study or occurs in the 30 day follow-up period, must be followed up to resolution, unless the event is considered by the investigator to be unlikely to resolve, or the patient is lost to follow-up.

AstraZeneca reserves the right to ask for further information/clarification on any adverse event that may be considered of interest.

At each visit the method of detecting AEs and SAEs in this study will be by:

- Information volunteered by the patient or patient's caregiver.

- Open-ended and non-leading verbal questioning of the patient at every visit such as the following: 'Have you had any health problems since the previous visit / last questioning?' – in the local or native language.
- Observation by the investigational team, other care providers or relatives.

If a patient withdraws from treatment for reasons other than disease progression and therefore continues to have tumor assessments, drug or procedure related SAEs must be captured until the patient is considered to have progressive disease, and therefore will have no further tumor assessments.

AEs will be graded according to the National Cancer Institute Common Terminology Criteria for AEs (NCI CTCAE Version 3, June 2003).

The following variables will be collected for each AE:

- AE description
- Onset date and time
- Resolution date and time
- Changes in CTCAE grade
- Action taken
- Treatments patient received for AE
- Outcome
- Causality (yes or no)
- Whether event constitutes an SAE

AEs will be coded using MedDRA (Medical dictionary for regulatory activities).

It is important to distinguish between serious and severe AEs. Severity is a measure of intensity, whereas seriousness is defined by the criteria in Section 4.8.1.1. An AE of severe intensity need not necessarily be considered serious. For example, nausea that persists for several hours may be considered severe nausea, but not a SAE. On the other hand, a stroke that results in only a limited degree of disability may be considered a mild stroke but would be a SAE.

Only AEs which caused the patient to permanently stop taking study treatment should be reported on the AE CRF as a permanent discontinuation of study treatment. The reason given for withdrawal/ permanent discontinuation of study treatment would be given as 'adverse event'.

After study completion, if an investigator learns of any SAEs, including death, at any time after a patient has completed the study and he/she considers there is a reasonable possibility that the event is related to AZD6244, the investigator should notify AstraZeneca Drug Safety.

Overdose

Doses of study treatment in excess of that specified in the clinical study protocol are considered to be an overdose.

Should an overdose (accidental or deliberate) occur, it must be reported in accordance with the procedures described in Section 8.3, Procedures in case of overdose, regardless of whether the overdose was associated with any symptom or not. All symptoms associated with the overdose should be reported as AEs.

Pregnancy

Should a pregnancy occur, patient should stop AZD6244 therapy immediately. Pregnancy must be reported in accordance with the procedures described in Section 8.4 Procedures in case of pregnancy. Pregnancy in itself is not regarded as an AE unless there is a suspicion that an investigational product may have interfered with the effectiveness of a contraceptive medication.

Abnormal laboratory findings/vital signs

Protocol mandated laboratory/vital sign abnormalities would not be reported as AEs unless any criterion for an SAE is fulfilled, the laboratory/vital sign abnormality causes the patient to discontinue treatment with the investigational product (IP) or if the investigator has a strong belief that it should be reported as an AE. All abnormalities from protocol-mandated laboratory/vital measurements will summarized in the Clinical Study Report in the ‘Laboratory measurements and variables’ section.

If an abnormal laboratory value/vital sign is associated with a diagnosis or clinical signs or symptoms, then the diagnosis, sign or symptom would be reported as an AE and the associated laboratory result/vital sign would be considered additional information to support the diagnosis. This applies to both protocol-mandated measurements and those that are measured outside of protocol requirements.

Disease progression

Disease progression can be considered as a worsening of a patient’s condition attributable to the disease for which the IP is being studied. It may be an increase in the severity of the disease or and increases in the symptoms of the disease. Expected progression of the disease under study and/or expected progression of signs and symptoms of the disease under study, unless more severe in intensity or more frequent than expected for the patient’s condition, should not be reported as an AE. The development of new, or progression of existing metastasis to the primary cancer under study should be considered as disease progression and not an AE. **Events which are unequivocally due to disease progression must not be reported as an AE/SAE.**

Lack of efficacy

Where there is deterioration in the condition for which the study treatment is being used, there may be uncertainty as to whether this is lack of efficacy or constitutes an AE. In such cases, unless the AstraZeneca or reporting physician considers that the study treatment contributed to the deterioration, or local regulations state to the contrary, the deterioration should be considered to be lack of efficacy and not an AE.

Handling of deaths

All deaths that occur during the study, or within the 30-day follow-up period after the administration of the last dose of study treatment, must be reported as follows:

- Death, which is clearly as a result of disease progression, should be communicated to the study monitor at the next monitoring visit and should be documented in the CRF, but should not be reported as a SAE

- Where death is not due (or not clearly due) to progression of disease under study the AE causing the death must be reported to the study monitor as an SAE within 24 hours. The report should contain a comment regarding the co-involvement of progression of disease, if appropriate, and should assign a single primary cause of death together with any contributory causes

Deaths with an unknown cause should always be reported as a SAE but every effort should be made to establish a cause of death. A post-mortem may be helpful in the assessment of the cause of death, and if performed a copy of the post-mortem results should be forwarded to AstraZeneca Drug Safety within the usual timeframes.

New cancers

The development of a new cancer should be regarded as an AE and will generally meet at least one of the serious criteria. New primary cancers are those that are not the primary reason for the administration of the study treatment, or metastasis there of, and have developed after the inclusion of the patient into the study. Symptoms of metastasis or the metastasis itself should not be reported as an AE/SAE if they are considered to be disease progression.

Cardiorespiratory adverse events

All cardiac or respiratory adverse events should be assessed with chest X-ray, triplicate ECGs and oxygen saturation measurement.

Any events possibly with a cardiac etiology (e.g., congestive cardiac failure) a measure of cardiac ejection fraction (EF) and blood sample for BNP or NT-proBNP should be taken at the time of the event. For patients who develop fluid accumulation conditions such as peripheral edema or ascites (or experience worsening of pre-existing condition), follow-up should also include measurement of clinical chemistries (including electrolytes and albumin) and urinalysis.

Any events possibly with a respiratory etiology (eg, hypoxia demonstrated by a clinically significant drop in O_2 saturations) should be followed up by full PFTs and HRCT.

For dyspnea events, please see diagnostic and treatment algorithm. Figure 2

Figure 2 Dyspnea Treatment Algorithm

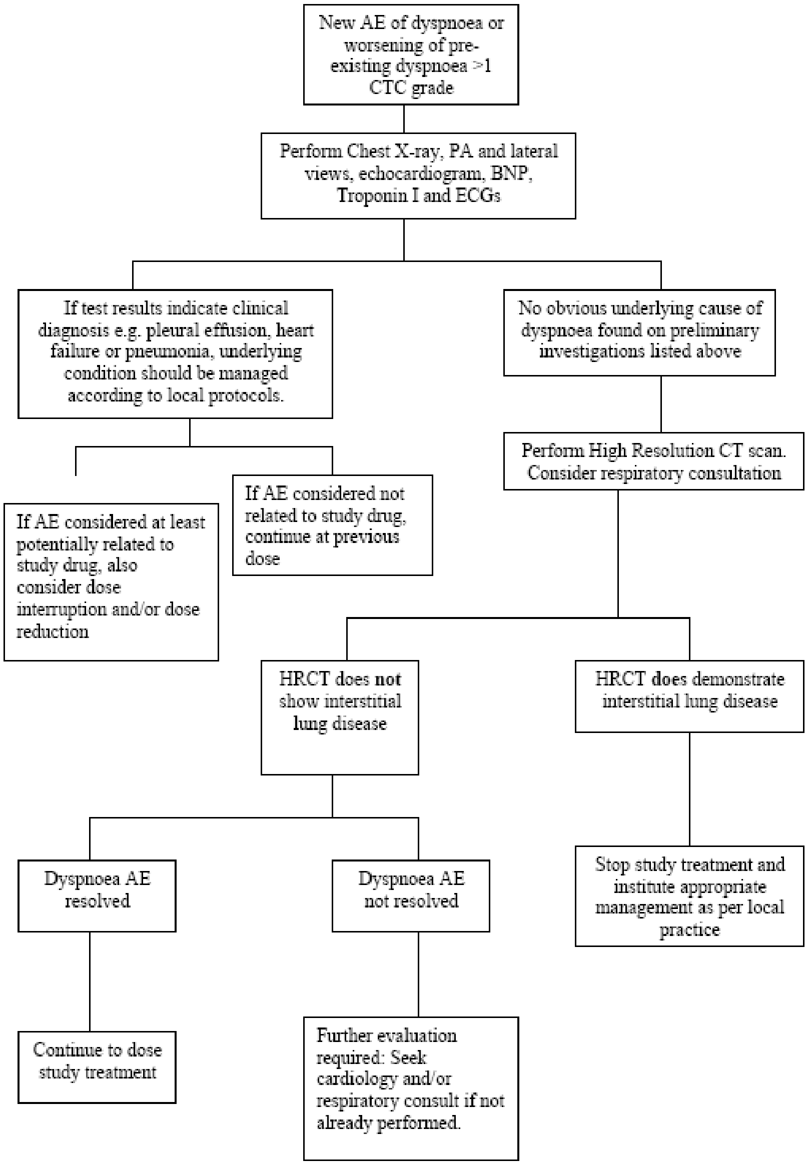

Figure 3 LVEF Algorithm

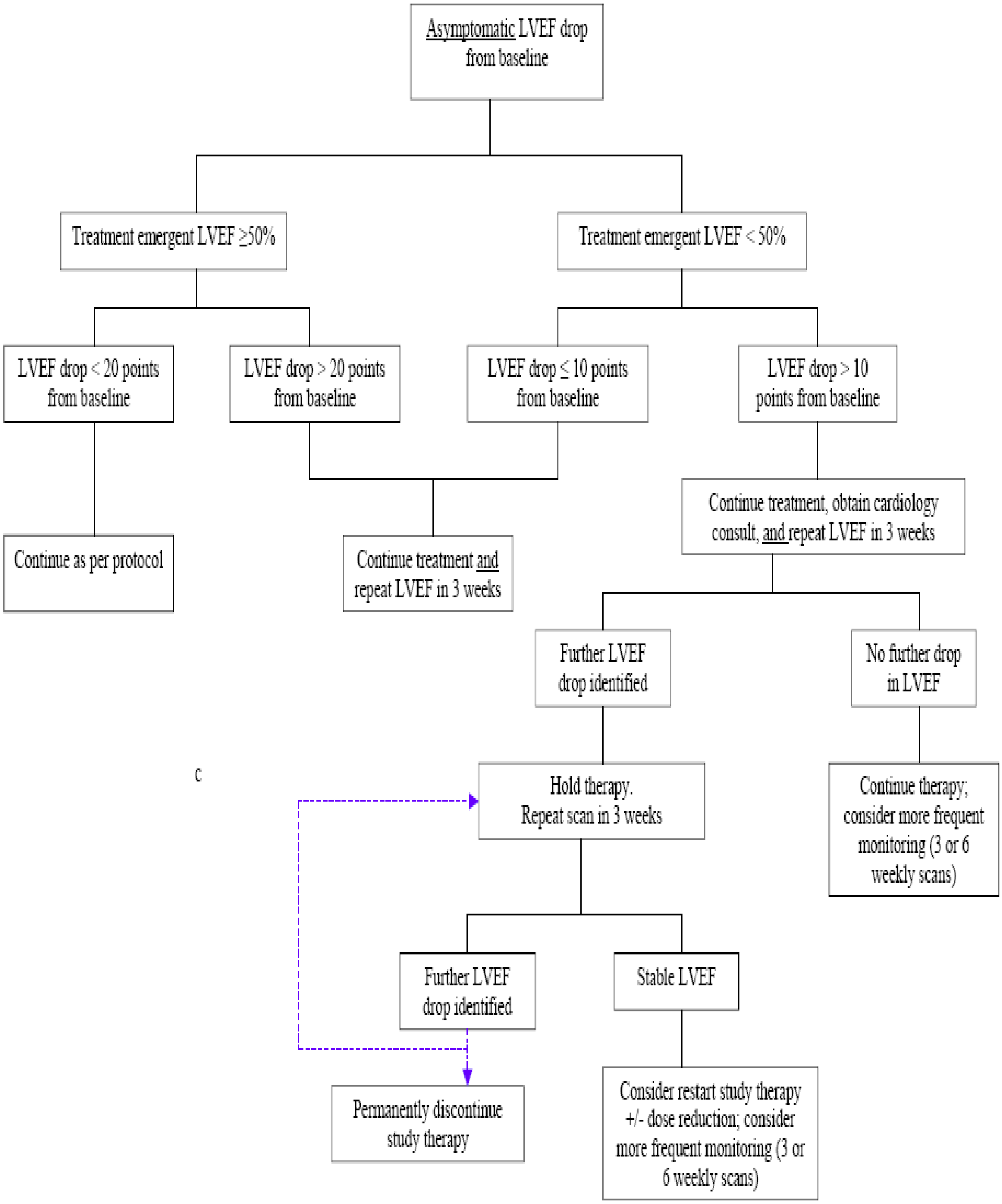

Asymptomatic decreases in ejection fraction

Please refer to Figure 3, Left ventricular ejection fraction (LVEF) algorithm. If the patient becomes symptomatic, refer for cardiology consult.

Visual Disturbances

All events of visual disturbance should be followed up with a complete ophthalmologic examination that includes visual acuity, visual fields, color vision, and slit-lamp examination **If a patient receiving AZD6244 experiences blurred vision (CTCAE grade ≥2) concurrent with neurological symptoms (e.g. seizure, altered mental status, headaches) suggestive of possible Reversible Posterior Leucoencephalopathy syndrome (RPLS), a MRI scan of the brain should be performed, if this is not possible a CT scan can be used. Scans should be repeated upon resolution of neurological symptoms or upon clinically significant neurological deterioration.**

Diarrhea

All SAEs of diarrhea should be followed up with a complete electrolyte assessment.

Proteinuria

For patients who develop proteinuria >2+ (or experience worsening of pre-existing condition), a repeat urinalysis should be performed. If this second test confirms the laboratory abnormality the full 24-hour urine collection should be performed to measure urinary protein excretion.

4.8.1.3 Reporting of serious adverse events

Investigators and other site personnel must inform appropriate AstraZeneca representatives of any SAE that occurs in the course of the study within 1 day (i.e., immediately but no later than the end of the next business day) of when he or she becomes aware of it

Follow-up information on SAEs must also be reported by the investigator within the same time frames.

If a non-serious AE becomes serious, this and other relevant follow-up information must also be provided to AstraZeneca within 1 day as described above. For a non-serious AE that become serious but which is not fatal or life-threatening a report should be received within 5 days.

The AstraZeneca representative will work with the investigator to compile all the necessary information and ensure that the appropriate AstraZeneca Drug Safety Department receives a report by day one for all fatal and life-threatening cases and by day five for all other SAEs.

All SAEs have to be reported, whether or not considered causally related to the investigational product or to the study procedure(s). All SAEs will be recorded in the case report form. The investigator and/or the sponsor are responsible for informing the Institutional Review Board and/or the Regulatory Authority of the SAE as per local requirements.

5. STUDY MANAGEMENT

5.1 Monitoring

5.1.1 Study monitoring

The monitoring of this study will be performed in accordance with the principles of Good Clinical Practice (GCP) as laid out in the International Conference on Harmonisation (ICH) document "Good Clinical Practice: Consolidated Guideline".

Before first patient into the study, a representative of AstraZeneca will visit the investigational study sites to:

- Determine the adequacy of the facilities
- Discuss with the investigator(s) (and other personnel involved with the study) their responsibilities with regard to protocol adherence, and the responsibilities of AstraZeneca or its representatives. This will be documented in a Clinical Study Agreement between AstraZeneca and the investigator

During the study, a monitor from AstraZeneca or company representing AstraZeneca will have regular contacts with the study site, including visits to:

- Provide information and support to the investigator(s)
- Confirm that facilities remain acceptable
- Confirm that the investigational team is adhering to the protocol, that data are being accurately recorded in the CRFs, and that investigational product accountability checks are being performed
- Perform source data verification

The monitor or another AstraZeneca representative will be available between visits if the investigator(s) or other staff at the center needs information and advice.

Monitoring will routinely be performed prior to the transfer of data to Data Management. For any change or amendment to data previously retrieved via the CRF, the monitor will assure that the Investigator(s) has reported to the Sponsor the change (or amendment), date, and reason for change in written form

5.1.2 Data verification

It is a prerequisite of this study that the study monitor has direct access to source data for data verification. This will be done by comparing data in the CRFs with those in the patient's medical notes (permission from the patient will be sought as part of the consent process), patient's medical records at the hospital/clinic or practice, and other records relevant to the

study. Such verification is an essential element of quality control, as it allows the rectification of transcription errors and omissions.

5.2 Audits and inspections

Authorized representatives of AstraZeneca, a regulatory authority, an Institutional Review Board may visit the center to perform audits or inspections, including source data verification. The purpose of an AstraZeneca audit or inspection is to systematically and independently examine all study-related activities and documents to determine whether these activities were conducted, and data were recorded, analyzed, and accurately reported according to the protocol, GCP guidelines of the ICH and any applicable regulatory requirements. The investigator should contact AstraZeneca immediately if contacted by a regulatory agency about an inspection at his or her center.

5.3 Training of staff

The principal investigator will maintain a record of all individuals involved in the study (medical, nursing and other staff). He or she will ensure that appropriate training relevant to the study is given to all of these staff, and that any new information of relevance to the performance of this study is forwarded to the staff involved.

5.4 Changes to the protocol

Study procedures will not be changed without the mutual agreement of the principal investigator and AstraZeneca.

If it is necessary for the study protocol to be amended, the amendment and/or a new version of the study protocol must be notified to or approved by each Institutional Review Board, and in many countries also the local regulatory authority, before implementation. Local requirements must be followed.

If an administrative change is required, such a change must be notified to or approved by each Institutional Review Board according to local requirements.

If a protocol amendment requires a change to a particular center’s Informed Consent Form, then AstraZeneca and the center’s Institutional Review Board must be notified. Approval of the revised Master Informed Consent Form by AstraZeneca and by the Institutional Review Board is required before the revised form is used.

AstraZeneca will distribute amendments and new versions of the protocol to each principal investigator(s) who in turn is responsible for the distribution of these documents to his or her Institutional Review Board, and to the staff at his or her center. The distribution of these documents to the regulatory authority will be handled according to local practice.

5.5 Study agreements

The principal investigator at each center must comply with all the terms, conditions, and obligations of the study agreement for this study. In the event of any inconsistency between this protocol and the study agreement, this protocol shall prevail.

5.6 Study timetable and end of study

The study will not begin before execution of formal agreement between AstraZeneca and the clinical study site(s), and approval has been granted by the IRB and necessary regulatory authorities. The study began in December 2007 and is expected to be completed by March 2011.

The end of study is defined as 30 days after the last patient discontinues study treatment. However, final analysis will occur 6 months after the last patient recruited starts investigational product.

At this time point, the clinical study database will be closed to new data. Patients are however, permitted to receive any study treatment beyond the closure of the database if, in the opinion of the investigator, they are continuing to derive clinical benefit from study treatment, in the absence of significant toxicity. Patients who do continue to receive study treatment beyond the defined end of study will be followed up to according to the investigator site standard of care and investigator judgment. Investigators must continue to report all SAEs to the AstraZeneca Patient Safety department until 30 days after study treatment is discontinued, in accordance with Section 4.8.1.3. Any AEs that are unresolved at the patient's last AE assessment in the study are followed up by the investigator for as long as medically indicated, but without further recording in the paper CRF. AstraZeneca reserves the right to ask for further information/clarification on any AE that may be considered of interest.

5.7 Data management

5.7.1 Case report forms

Paper CRFs will be provided for the recording of data. The CRF will be in 2-part no carbon required paper (2-part NCR). The top sheet of the CRFs will be collected by the monitor and sent to AstraZeneca. The copy will be retained by the investigator. Data should be recorded legibly onto the forms in black or blue permanent ballpoint ink pen. If any data are not available, omissions will be indicated on the record forms. Corrections should be made legibly and initialed and dated by approved site personnel. Correction fluid or covering labels must not be used.

The monitor representing AstraZeneca will check data at the monitoring visits at the investigational site. The CRF must be signed by the Investigator or sub Investigator, who should be a currently licensed MD or DO and able to make medical assessment/decision to confirm that the CRF has been checked for accuracy and completeness; this signature should include review of all the latest test results and safety data.

Detailed instructions regarding CRF completion will be provided on the Instructions for the Investigator.

Data from the completed CRFs will either be independently double-data entered using an external system and then loaded onto AstraZeneca's clinical study database (AMOS) using the AHOY load program or data entry will be done directly onto AMOS via single data entry followed by proof reading."

Data queries arising from validation checks, such as missing, impossible or inconsistent recordings in the CRFs will be detailed on data query forms (DQFs) on 2- part NCR forms that will be forwarded to the investigator. After resolution by appropriate site personnel and source data verification by the monitor, the top white original signed copy is collected by the monitor and sent to AstraZeneca. The 2nd copy is retained by the investigator.

All data captured electronically will be transferred directly to the appropriate database. Validation and quality control of this is the responsibility of the provider.

The Medical Dictionary for Regulatory Activities (MedDRA) will be used to code all medical conditions and surgeries reported on the Medical and Surgical History, Adverse Events/Serious Adverse Events, Deaths and if applicable, all medical conditions and surgeries reported the Medical and Surgical History CRFs. The AstraZeneca Drug Dictionary (AZDD) will be used to code all concomitant medications and previous cancer therapy(s). The MedDRA and AZDD dictionaries used at the beginning of the study will be the most current version. As new versions of the dictionaries are released, version control will be implemented according to the study specific coding guidelines.

6. PHARMACOKINETIC, PHARMACODYNAMIC, SAFETY, BIOMARKER AND STATISTICAL METHODOLOGY

6.1 Pharmacokinetic / pharmacodynamic evaluation

6.1.1 Calculation or derivation of pharmacokinetic variables

Where data allow, the following PK parameters will be determined following administration of chemotherapy alone, following administration of AZD6244 alone and following administration of AZD6244 and chemotherapy together. Additional parameters may be determined if deemed appropriate.

- AZD6244: C_{max}, t_{max}, $AUC_{(0-12)}$
- N-desmethyl AZD6244 C_{max}, t_{max} , $AUC_{(0-12)}$
- Chemotherapies: C_{max}, t_{max}, $AUC_{(0-12)}$

The PK parameters will be derived using noncompartmental analysis. The maximum plasma concentrations (C_{max}) and the time to reach the maximum plasma concentrations (t_{max}) will be

determined by visual inspection of the plasma concentration-time profiles. The area under the plasma concentration-time curve from zero to 12 hours post dose, $AUC_{(0-12)}$, will be calculated by the linear trapezoidal rule. Where more than one maxima occurs, the reported value will be assigned to the first occurrence.

6.1.2 Calculation or derivation of pharmacodynamic variables

No pharmacodynamic parameters are planned for this study.

6.1.3 Calculation or derivation of pharmacokinetics/pharmacodynamics

No pharmacodynamic parameters are planned in the study. However, if there are adverse events or safety end points that appear to be drug related, the relationship between plasma AZD6244 and/or N-desmethyl AZD6244 concentrations/exposure (and/or chemotherapy if appropriate) and the safety data may be investigated using either graphical means or appropriate PK/PD software if warranted.

6.2 Safety evaluation

6.2.1 Calculation or derivation of safety variables

Not applicable.

6.3 Biomarker and Genetic markers as a co-variable

Not applicable.

6.4 Statistical methods and determination of sample size

6.4.1 Statistical evaluation

All statistical analyzes will be the responsibility of

6.4.2 Description of outcome variables in relation to objectives and hypotheses

The primary objectives of this study are:

To assess the safety and tolerability of twice daily oral doses of AZD6244 when administered in combination with the selected chemotherapies: docetaxel, dacarbazine, erlotinib, and temsirolimus. This objective will be assessed by AEs, ECGs, vital signs, hematology, clinical chemistry, urinalysis, physical examinations, MUGA scan/Echocardiogram, and ophthalmologic examination.

and

To determine the pharmacokinetics of AZD6244 and the chemotherapies when dosed together.

The secondary objective is to determine the highest tolerated dose of AZD6244 when administered in combination with the selected chemotherapies.

Where the data are available, the secondary PK objectives will be assessed by derivation of PK parameters for both AZD6244 (and N-desmethyl AZD6244) and the selected chemotherapies, when administered alone and in combination according to the list in Section 6.1.1.

An exploratory objective is to make a preliminary assessment of efficacy by measuring Objective Response Rate (ORR) when AZD6244 is given in combination with selected standard chemotherapies.

Part B only: the secondary objective of this study is to use larger cohorts of chemotherapy in combination with AZD6244 at the highest tolerated dose established in Part A, to identify the dose of AZD6244 that can be combined with standard chemotherapies.

6.4.3 Description of analyzes sets

Three populations, safety population, evaluable for dose escalation evaluation (Part A), and evaluable for PK analysis are defined in the table below:

Table 14 Description of analyzes sets

Population	Definition
Safety Population	This will include all patients who received a least one dose of study medication.[a]
Evaluable for Dose Escalation (Part A)	This is a subset of the safety population that includes all patients who received approximately 80% of the defined doses of AZD6244 in cycle 1, and completed at least 28 days of therapy from cycle 1/day 1, provided PK data, and have all safety evaluations performed or experienced a DLT.
Evaluable for PK analysis	This is a subset of the safety population that includes all patients who provide docetaxel, dacarbazine, erlotinib, temsirolimus , AZD6244 and/or N-desmethyl AZD6244 concentration-time data.

[a] If a patient discontinues study medication and starts another cancer treatment, all data after 30-day follow-up period for that patient will be excluded from the assessment of safety.

6.4.4 Method of statistical analysis

No formal statistical hypothesis testing will be performed on the data from this study.

6.4.5 Determination of sample size

Part A of the study is not formally powered but is designed to provide adequate tolerability, safety, and pharmacokinetic data, and to ensure at least 18 evaluable patients are recruited at the dose deemed to be the highest tolerated dose.

The expansion at the highest tolerated dose provides further safety data to give a better estimate of the true DLT rate.

For example if the highest tolerated dose cohort had only 6 patients then:

- If no DLTs are observed it can be concluded that the true DLT rate is less than 30% with 90% confidence

- If 1 DLT (16.7%) is observed it can be concluded that the true DLT rate is less than 50% with 90% confidence

However, with the additional 12 evaluable patients (18 evaluable patients in total):

- If no DLTs are observed, it can be concluded that the true DLT rate is less than 12% with 90% confidence

- If fewer than 3 DLTs are observed, it can be concluded that the true DLT rate is less than 27% with 90% confidence

6.5 Interim analyzes

No formal statistical interim analysis will be undertaken.

6.6 Data presentation

All safety, tolerability, and pharmacokinetic data relating to each treatment arm will be listed and summarized as detailed below. Part A and Part B data will be listed separately but summaries will contain data from both Parts. Figures will contain data from both Parts, with different plotting symbols or line types to distinguish Parts.

6.6.1 Safety Data

Appropriate summaries of all safety data will be produced as defined below and in the SAP.

AEs (both in terms of MedDRA preferred terms and CTCAE grade) will be listed individually by patient and treatment group. For patients who have a dose modification in Part A, all AEs (due to drug or otherwise) will be assigned to the initial dose group. For patients who have a dose modification in Part B, all AEs (due to drug or otherwise) will be assigned to the initial dose group in Part B. The number of patients experiencing each AE will be summarized by treatment group and severity.

Any AE occurring before treatment (i.e., before study Day 1) will be included in the data listings but will not be included in the summary tables of adverse events. In addition, a separate data listing of pre-treatment AEs will be produced.

Any AE occurring within 30 days of AZD6244 discontinuation will be included in the AE summaries. Any events in this period that occur after a patient has received further therapy for cancer (following discontinuation of AZD6244) will be flagged in the data listings. AEs

occurring more than 30 days after discontinuation of AZD6244 will be listed separately, but not included in the summaries.

ECGs, vital signs, hematology, clinical chemistry, urinalysis, physical examinations, MUGA scan/Echocardiogram, and ophthalmologic examination will be listed individually by patient and treatment group, and summarized by treatment group, as follows:

Hematology, clinical chemistry, vital signs and ECG data will be suitably summarized by treatment group for all patients by:

- summary statistics of observed values: mean, standard deviation, minimum, maximum, n
- summary statistics of change from baseline values: mean, standard deviation, minimum, maximum, n
- Individual patient listings: including highlighting of hematology and clinical chemistry values outside the local laboratory reference ranges

Qualitative assessments will be summarized for all patients using the number of patients with results of negative, trace or positive. Graphical presentations of safety data will be presented as is deemed appropriate.

6.6.2 PK data

The concentration-time data of AZD6244 and the chemotherapies, along with the derived pharmacokinetic variables, will be listed for each patient per treatment and dosing day and summarized appropriately.

6.6.3 Tumor response data

Summaries of ORR by initial treatment will be produced, for all patients from the safety population who have a baseline RECIST assessment and at least one follow-up RECIST assessment. Changes in target tumor measurement length will be presented graphically.

6.6.4 Pharmacogenetic data

In the case of genotypic data, only the date the patient gave consent to participation in the genetic component of the study and the date the blood sample was taken from the patient will be recorded in the paper CRF and database. Further details are given in Appendix D.

6.7 Reporting of Genotypic Results (pharmacogenetics)

Refer to Appendix D

6.8 Data monitoring committee

Not applicable

6.8.1 Study Safety Review Committee

A study SRC will meet during Part A following each dose step (i.e., following at least 28 days after start of therapy from Cycle 1/Day 1 for each dose level). The SRC comprising of the Investigators, the Medical Science Director, the Study Team Physician, the Pharmacokineticist and the Global Drug Safety Physician (or nominated deputy in each case) will assess the available safety and PK data. The committee must include a minimum of 3 physicians at least 1 of who must be an investigator, but may also include other team members, if appropriate (e.g., statistician, etc). The SRC will remain in place for Part B.

Further details of the SRC can be found in the Safety Review Committee Guidelines. SM - update with agreed wording.

7. ETHICS

7.1 Ethics review

The final study protocol, including the final version of the Informed Consent Form, must be approved or given a favorable opinion in writing by an Institutional Review Board as appropriate. The investigator must submit written approval to AstraZeneca before he or she can enroll any patient into the study.

The Principal Investigator is responsible for informing the Institutional Review Board of any amendment to the protocol in accordance with local requirements. In addition, the Institutional Review Board must approve all advertising used to recruit patients for the study. The protocol must be re-approved by the Institutional Review Board annually, as local regulations require.

The Principal Investigator is also responsible for providing the IRB with reports of any serious and unexpected adverse drug reactions from any other study conducted with the investigational product. AstraZeneca will provide this information to the Principal Investigator.

Progress reports and notifications of serious and unexpected adverse drug reactions will be provided to the Institutional Review Board according to local regulations and guidelines.

Where there is a genetic component to the study, approval must be obtained for the genetic component of the study and the genetic informed consent process from the IRB/IEC. It must be clearly stated in the approval that the genetic component of the study is approved. The investigator must submit a written approval to AstraZeneca before any patient participates in the genetic component of the study.

7.2 Ethical conduct of the study

The study will be performed in accordance with the ethical principles that have their origin in the Declaration of Helsinki and are consistent with Good Clinical Practice, applicable regulatory requirements and the AstraZeneca policy on Bioethics.

7.3 Informed Consent

The principal investigator at each center will ensure that the patient is given full and adequate oral and written information about the nature, purpose, possible risk and benefit of the study. Patients must also be notified that they are free to discontinue from the study at any time. The patient should be given the opportunity to ask questions and allowed time to consider the information provided.

The patient's signed and dated informed consent must be obtained before conducting any procedure specifically for the study.

The principal investigator must store the original, signed Informed Consent Form. A copy of the Informed Consent Form must be given to the patient.

If modifications are made according to local requirements, the new version has to be approved by AstraZeneca.

The biomarker and genetic components of this study are optional and the patient may participate in other components of the study without participating in the genetic and/or biomarker component. To participate in the genetic and/or biomarker component(s) of the study, the patient must sign and date both the consent form for the non-genetic component(s) of the study and the genetic and/or biomarker components of the study. Copies of all signed and dated consent forms must be given to the patient and the originals filed at the study centre. The principal investigator(s) is responsible for ensuring that consent is given freely and that the patient understands that they may freely discontinue the genetic and/or biomarker component of the study at any time.

7.4 Patient data protection

The Master Informed Consent Form will incorporate (or, in some cases, be accompanied by a separate document incorporating) wording that complies with relevant data protection and privacy legislation. Pursuant to this wording, patients will authorize the collection, use and disclosure of their study data by the Investigator and by those persons who need that information for the purposes of the study.

The Master Informed Consent Form will explain that study data will be stored in a computer database, maintaining confidentiality in accordance with national data legislation. All data computer processed by AstraZeneca will be identified by Ecode/study code/ initials.

The Master Informed Consent Form will also explain that for data verification purposes, authorized representatives of AstraZeneca, a regulatory authority, an Institutional Review

Board may require direct access to parts of the hospital or practice records relevant to the study, including patients' medical history.

8. PROCEDURES IN CASE OF EMERGENCY, OVERDOSE OR PREGNANCY

8.1 AstraZeneca emergency contact procedure

In the case of a medical emergency, contact AstraZeneca personnel shown below.

Role in the study	Name	Address and Telephone number
Study Delivery Team Leader		Tel:
Study Delivery Team Physician		Tel:
Business hours emergency cover		8:00 AM – 7:00 PM EST<br>Tel:
After hours emergency cover		7:009 PM – 8:00 AM EST<br>Tel:

For Serious Adverse Event reporting send reports to:

•

8.2 Procedures in case of medical emergency

The principal investigator(s) is responsible for ensuring that procedures and expertise are available to cope with medical emergencies during the study. **A medical emergency usually constitutes an SAE and should be reported as such, see Section 4.8.1.3.**

8.3 Procedures in case of overdose

There is currently no known antidote to AZD6244. In the event of an overdose, symptomatic, supportive care should be given as required and all details should be recorded within the AE and Con Med CRFs.

- Use of study medication in doses in excess of that specified in the protocol should not be recorded in the CRF as an AE of 'Overdose' unless there are associated symptoms or signs.

- An Overdose with associated SAEs should be recorded as the SAE diagnosis/symptoms on the relevant AE forms in the CRF.

- An Overdose with associated non-serious AEs should be recorded as the AE diagnosis/symptoms on the relevant AE forms in the CRF. In addition, the Overdose should be reported on the separate AZ “Clinical Study Overdose Report Form.”

- An Overdose without associated symptoms should not be recorded as an AE in the CRF. The Overdose should be reported on the separate AZ “Clinical Study Overdose Report Form”.

8.4 Procedures in case of pregnancy

Pregnancy itself is not regarded as an adverse event unless there is a suspicion that the investigational product under study may have interfered with the effectiveness of a contraceptive medication. However, the outcome of all pregnancies (spontaneous miscarriage, elective termination, normal birth or congenital abnormality) must be followed up and documented even if the patient was discontinued from the study.

All reports of congenital abnormalities/birth defects are SAEs. Spontaneous miscarriages should also be reported and handled as SAEs. Elective abortions without complications should not be handled as AEs. All outcomes of pregnancy must be reported to AstraZeneca on the pregnancy outcomes report form.

9. REFERENCES

Kolch 2000
Kolch W. Meaningful relationships: the regulation of the Ras/Raf/MEK/ERK pathway by protein interactions. Biochem J 2000;351:289-305.

LoRusso et al 2005
Lorusso P, Krishnamurthi S, Rinehart JR, Nabell L, Croghan G, Varterasian M, et al. A phase 1-2 clinical study of a second generation oral MEK inhibitor, PD 0325901 in patients with advanced cancer. J Clin Oncol; 2005:23:3011.

Perez-Soler et al 2005
Pérez-Soler R, Delord J, Halpern A, et al. HER1/EGFR inhibitor-associated rash: future directions for management and investigation outcomes from the HER1/EGFR inhibitor rash management forum. The Oncologist 2005;10,:345-356.
